# Transcriptomic Events Involved in Melon Mature-Fruit Abscission Comprise the Sequential Induction of Cell-Wall Degrading Genes Coupled to a Stimulation of Endo and Exocytosis

**DOI:** 10.1371/journal.pone.0058363

**Published:** 2013-03-06

**Authors:** Jorge Corbacho, Félix Romojaro, Jean-Claude Pech, Alain Latché, Maria C. Gomez-Jimenez

**Affiliations:** 1 Department of Plant Physiology, University of Extremadura, Avda de Elvas s/n, Badajoz, Spain; 2 CEBAS-CSIC, Campus de Espinardo, Murcia, Spain; 3 UMR990 INRA/INP-ENSA Toulouse, Avenue de l'Agrobiopole, Castanet-Tolosan, France; Instituto de Biología Molecular y Celular de Plantas, Spain

## Abstract

**Background:**

Mature-fruit abscission (MFA) in fleshy-fruit is a genetically controlled process with mechanisms that, contrary to immature-fruit abscission, has not been fully characterized. Here, we use pyrosequencing to characterize the transcriptomes of melon abscission zone (AZ) at three stages during AZ-cell separation in order to understand MFA control at an early stage of AZ-activation.

**Principal Findings:**

The results show that by early induction of MFA, the melon AZ exhibits major gene induction, while by late induction of MFA, melon AZ shows major gene repression. Although some genes displayed similar regulation in both early and late induction of abscission, such as *EXT1-EXT4*, *EGase1*, *IAA2*, *ERF1*, *AP2D15*, *FLC*, *MADS2*, *ERAF17*, *SAP5* and *SCL13* genes, the majority had different expression patterns. This implies that time-specific events occur during MFA, and emphasizes the value of characterizing multiple time-specific abscission transcriptomes. Analysis of gene-expression from these AZs reveal that a sequential induction of cell-wall-degrading genes is associated with the upregulation of genes involved in endo and exocytosis, and a shift in plant-hormone metabolism and signaling genes during MFA. This is accompanied by transcriptional activity of small-GTPases and synthaxins together with tubulins, dynamins, V-type ATPases and kinesin-like proteins potentially involved in MFA signaling. Early events are potentially controlled by down-regulation of MADS-box, AP2/ERF and Aux/IAA transcription-factors, and up-regulation of homeobox, zinc finger, bZIP, and WRKY transcription-factors, while late events may be controlled by up-regulation of MYB transcription-factors.

**Significance:**

Overall, the data provide a comprehensive view on MFA in fleshy-fruit, identifying candidate genes and pathways associated with early induction of MFA. Our comprehensive gene-expression profile will be very useful for elucidating gene regulatory networks of the MFA in fleshy-fruit.

## Introduction

Melon (*Cucumis melo* L.), an important crop worldwide and an annual diploid plant, has a high intra-specific genetic variation and a small genome size (454 Mb), which can be exploited to dissect biological processes of great technological importance, among them flavour development and textural changes that occur during fruit ripening [1], [2]. The amount of genomic information available for melon has been increasing recently. Efforts have been made to generate melon genetic maps [3]–[5], the construction of bacterial artificial chromosome (BAC) libraries [6], the development of oligo-based microarrays [7], [8], the production of TILLING and EcoTILLING platforms [9], and the development of a collection of near isogenic lines (NILs) [10]. Several large expressed sequence tag (EST) datasets have recently been generated in melon, including approximately 350,000 ESTs generated [11], using the 454 pyrosequencing technologies, and an additional 127,000 ESTs generated using the traditional Sanger sequencing approach [12], Recentely, the genome of melon has been sequenced under the Spanish Genomics Initiative MELONOMICS [13].

Melon has a great potential for becoming a model for understanding important traits in fruiting crops [2]. The melon comprises climacteric and non-climacteric genotypes. The melon-fruit ripening of several cultivated genotypes and wild ecotypes is climacteric and often associated with mature-fruit abscission (MFA) [2]. Typical climacteric phenotypes with high ethylene production, such as *Cucumis melo* var cantalupensis, have a fast ripening rate and short shelf-life. In cantaloupe as in other climacteric fruit, exogenous ethylene can prematurely induce fruit abscission, ethylene production, and ripening. Cantaloupe Charentais melons (cv Védrantais) have been transformed with an antisense construct of an ACC oxidase cDNA driven by the 35S promoter [14]. A line of the antisense lines generated showed a reduction of ethylene production by more than 99.5% which resulted in strong effects on the ripening and MFA processes [15]. Thus, the climateric increase in ethylene production is responsible of both fruit ripening and induction of MFA. Melon genotypes without MFA or without ethylene burst also exist and are, therefore, non-climacteric, as *C. melo* var inodorus, unable to produce autocatalytic ethylene, generally have a slow ripening rate associated with a long shelf-life or as Songwhan Charmi PI 161375 (*C. melo* var *chinensis*) [2]. By studying the segregation of the activation of the abscission zone (AZ) and ethylene production on a population of recombinant cantaloupe Charentais × PI 161375 inbred lines, we have previously demonstrated that both the MFA and climacteric characters were controlled by two duplicated independent loci (Al-3 and Al-4) and that the intensity of ethylene production was controlled by at least four quantitative trait loci (QTLs) localized in other genomic regions [16]. The QTLs associated with ethylene production and respiration rate in this work were not located at the same position with the Al loci described, and none of the Al loci matched with known genes of the ethylene biosynthetic or signaling pathways [16]. Taken together, these data suggest that both the MFA as well as climacteric characters are under complex regulation.

In this study, we seek to elucidate the molecular bases of MFA in melon, as a model organism for the study of fleshy fruit abscission and ripening [2], [16]. In fleshy fruit abscission, ethylene signaling and biosynthesis have been investigated in immature-fruit such as peach [17], [18], persimmon [19], and apple [20], as well as in MFA such as in apple [21] and in olive [22]. Recent global transcriptome studies have provided information about immature-fruit abscission in apple [23]. However, the global transcriptome analysis during MFA has not been studied in any species having fleshy fruit. Here, we present a comprehensive study of gene-expression profiles in melon fruit-AZ at crucial stages during AZ-cell separation to gain a complete picture of the genes and metabolic processes involved in the MFA, with particular emphasis on MFA control at a stage of AZ-activation. To this end, a pyrosequencing analysis was performed for the fruit-AZs in melon. Assembly of roughly 134 Mb of transcript sequences yielded more than 9,000 significant proteins. Hierarchical clustering of the 4,801 differentially expressed genes, indicated a transcriptional cascade in which relatively larger numbers of genes were early-induced during MFA, and few genes were late-induced transiently in melon AZ. Specific candidate genes that have not previously been reported to be associated with abscission, and metabolic pathways were identified to be involved in early and late induction of MFA. The results reveal the distinctiveness of the early transcriptome in comparison with that of the late transcriptome during MFA in melon.

## Results and Discussion

### Identification of Fruit-AZ Genes by Melon Pyrosequencing and Gene Ontology Groups in Early and Late Induction of MFA

**Figure 1:**
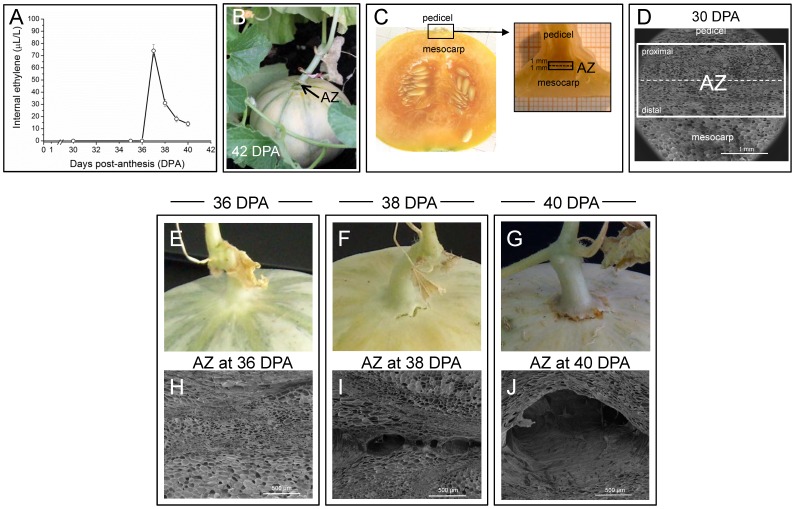
Ethylene production and anatomical observation of the fruit AZ of Charentais melon (*C. melo* var. cantalupensis Naud, ‘Védrantais’). (A) Internal ethylene concentration in melon fruit of Védrantais from 30–40 days post-anthesis (DPA). (B) Védrantais melon fruit at the time abscission is complete (42 DPA). Appearance of the mature-fruit AZ, located between the pedicel and fruit, after fruit abscission at 42 DPA. Arrows point to the AZ of Védrantais melon fruit. (C) Photographs of a longitudinal section of Védrantais fruit illustrating the position of the tissue sample of the AZ (black box) for RNA extraction and anatomical changes. Fruit AZs were manually dissected with a razor blade and separated by cutting approximately 1 mm on each side of the abscission fracture plane. The broken line indicates the position of the abscission fracture plane. (D) Scanning electron micrograph (SEM) of a longitudinal section illustrating the AZ of Védrantais fruit at 30 DPA, showing that the tissue samples used in this study (white box) were strongly enriched in mature fruit AZ cells. The broken line indicates the position of the abscission fracture plane. (E, F, G) Macro-photographs at 36, 38, and 40 DPA showing the aspect of the fruit area where abscission takes place. The external evidence of separation is a crack between the pedicel and fruit, first evident at 38 DPA. (H, I, J) SEM of the tissue sample of the AZ of Védrantais fruit at 36 (fruit-AZ pre-cell separation), 38 DPA (fruit-AZ partial cell separation), and 40 DPA (almost complete fruit-AZ cell separation and cell collapse). Scale bars: 1 mm in Fig. 1D and 500 µm in Figs. 1H,1I, 1J.

**Figure 2:**
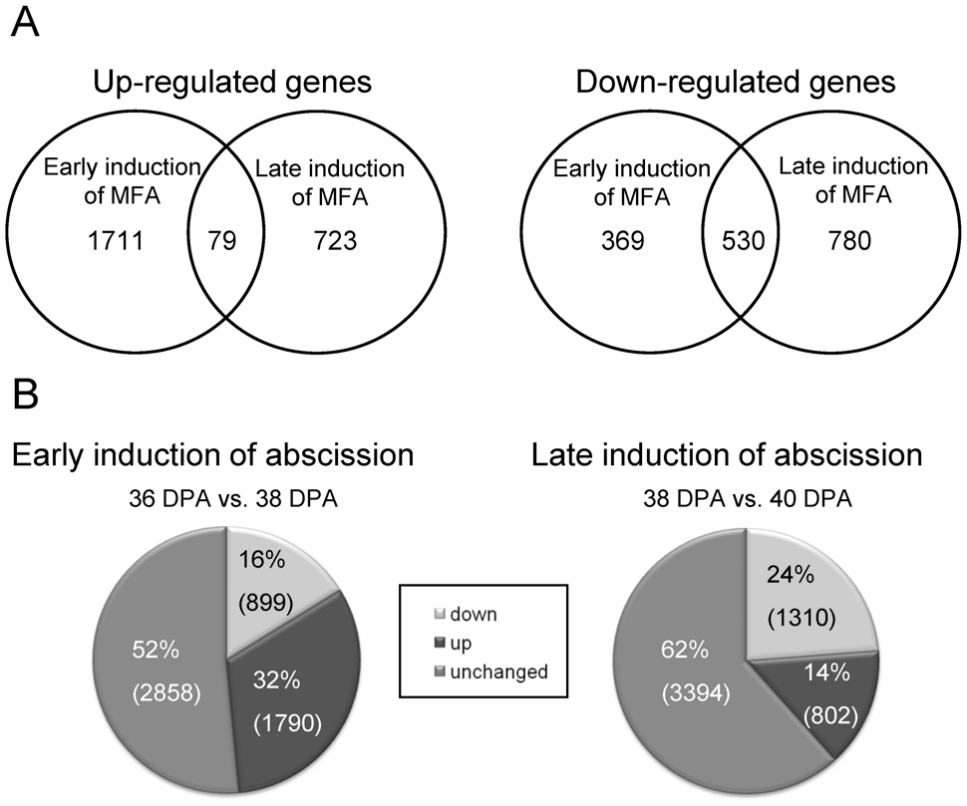
Melon-AZ genes during MFA. A, The differentially expressed genes in fruit-AZ during early [comparison of fruit-AZ at 36 DPA (pre-cell separation) and at 38 DPA (partial cell separation)], and late [comparison of fruit-AZ at 38 DPA (partial cell separation) and at 40 DPA (almost complete cell separation)] induction of melon MFA. B, The percentages of up-regulated and down-regualted genes during early and late induction of melon MFA.

Anatomical changes during mature-fruit abscission (MFA) of cantaloupe fruit include cell separation and cell collapse [24]. The critical anatomical change is that of cell separation, the nature and magnitude of which determine the manner and extent to which the fruit and pedicel ultimately split apart [24]. In Védrantais melon fruits, as a cantaloupe fruit, the climacteric increase of ethylene production synchronizes the ripening and MFA processes [2], [16]. As reported previously [15], [16], Védrantais fruits displayed a characteristic peak of ethylene production (Fig. 1A) at 37 days post-anthesis (DPA), and they mature and ripen on the vine for approximately 42 DPA, at which time they abscise (Fig. 1B). MFA was studied in the pedicel-fruit AZ of Védrantais fruit (Fig. 1B, 1C). The AZ tissues were manually dissected from longitudinal sections of the samples with a razor blade into pieces until a maximum width of 1 mm on each side of the abscission fracture plane (Fig. 1C). The AZ was characterized in Védrantais melon fruit by scanning electron microscopy (SEM) at 30, 36, 38, and 40 DPA, comprising 30–35 cell layers (Fig. 1D, 1H, 1I, 1J). Fig. 1D shown that the tissue samples used in this study (white box) were strongly enriched in AZ cells. The first report in the literature on anatomical aspect of abscission in cantaloupe fruit referred to melon cv. Powdery Mildew Resistant no. 45 [24]. In this melon, structural modifications in AZ cell walls are accompanied by histochemical changes which culminate in the abscission of the fruit from the pedicel during the 10-day period prior to abscission (from 32–42 DPA); however, in Védrantais melon the histochemical changes including increases of lignin take place during the 6-day period prior to abscission (from 37–42 DPA) (data not shown) and, thus, the extent of this period depends on the cultivar. Hence, in Védrantais melon, the activation of the ZA and the onset of ripening process take place at 37 DPA, and the induction of MFA occurs during a period from 37–42 DPA. It is interesting to note that the external evidence of separation is a crack between the pedicel and fruit, first evident at 38 DPA (Fig. 1F). During this period of MFA induction, the cell separation followed by collapse is characteristic of cortical parenchyma cells of the AZ [24]. Initially, the AZ of Védrantais fruit at 38 DPA reveals a small separation cavity within the zone (Fig. 1I), and subsequently at 40 DPA shows a cell separation and extensive cell collapse (Fig. 1J). To identify differences in transcript abundance related to aspects of the induction of MFA, the AZ transcriptomes of melon fruit during cell separation were compared: pre-cell separation (36 DPA) and first external evident of cell separation or partial cell separation (38 DPA; early induction of abscission). Also, in a second experiment, the fruit AZ transcriptomes of melon at partial cell separation (38 DPA; early induction of abscission) and almost complete cell separation and cell collapse (40 DPA; late induction of abscission) were similarly compared (Fig. 1). These three samples (AZ at 36, 38, and 40 DPA) represent critical physiological and anatomical changes during the period prior to abscission at 42 DPA, at that time MFA is complete (Fig. 1). This emphasizes the value of characterizing multiple time-specific abscission transcriptomes. After preparation of the cDNA libraries and their pyrosequencing, a total of 134 Mb of sequences (483,704 good-quality sequence reads) were obtained for the three samples. These sequences were assembled into 14,162 contigs, and after gene modeling into 12,871 isotigs (2,792 for 36 DPA, 7,315 for 38 DPA, and 2,764 for 40 DPA, respectively) (Table 1, Figure S1), of which 10,513 were isotigs with functional annotation. About a third of the isotigs did not found similar protein sequences and therefore represent a source for new gene discovery. The detection of genes was based on BLASTX against the set of reference Uniprot proteins (See Material and Methods). The number of different genes detected considering the three samples as a whole was 5,953. Analysis of the levels of expression for the 10,513 annotated isotigs leaded to the identification of 4,801 differentially expressed genes (45%) in our experiments (P<0.01), which are hereafter referred to as group I, while the remaining 5,712 genes with either nondifferential representation or low read abundance are referred to as group II. Among the 4,801 differentially expressed genes (P<0.01), 2,689 genes were abscission-responsive in the first experiment (between the fruit AZ at 36 DPA and 38 DPA, early abscission induction), and 2,112 were abscission-responsive in the second experiment (between fruit AZ at 38 DPA and 40 DPA, late abscission induction). In the first experiment, 1,790 genes were up-regulated, and 899 were down-regulated in fruit AZ at 38 DPA in the experiment. In the second experiment, 802 genes were up-regulated, and 1,310 were down-regulated in fruit AZ at 40 DPA in the experiment (the UniProt IDs for abscission-responsive genes are listed in Tables S1,S2,S3,S4). A comparison of the genes which were abscission-responsive during early and late induction experiments indicated that 79 were up-regulated in both experiments, and that 530 were down-regulated in both experiments (Fig. 2A, Tables S5–S6), with the majority of the differentially expressed genes up-regulated (1,790 vs. 899 down-regulated genes) occurring during early induction of MFA (Fig. 2B), whereas the majority of the differentially expressed genes down-regulated (1,310 vs. 802 up-regulated genes) occurring during late induction of abscission in melon-fruit AZ (Fig. 2B).

**Table 1:** Results of the 454 sequencing runs.

Data	Fruit-AZ at 36 DPA	Fruit-AZ at 38 DPA	Fruit-AZ at 40 DPA
Raw reads	115,760	273,169	95,228
Raw nucleotides	35,885,600	87,414,080	29,520,680
Raw mean length	310	320	310
Clean and processed reads	115,643	272,930	95,131
Clean nucleotides	31,196184	77,108,803	25,853,453
Clean and processed mean length	270	282	272
Total number of contigs	3,067	8,154	2941
Average contig size	611	653	607
Total number of isotigs	2,792	7,315	2,764
Detected proteins	2,014	5,009	2,050
Proteins with GO annotations	1,387	3,558	1,486
Proteins with EC number annotation	272	767	352

For the analysis of which cellular processes are critical during MFA, transcripts were grouped by their expression signatures across the three samples. Hierarchical cluster analysis of group I genes enabled the identification of three major clusters, termed A, B, and C, which contained 795, 1,228 and 537 genes, respectively. These groups of genes were subsequently divided into three (A1, A2, A3), three (B1, B2 B3), and three (C1, C2, C3) subclusters, respectively (Figure S2). In general, transcripts that exhibited a transcription peak at 36, 38 or 40 DAP were grouped into cluster A, B, or C, respectively. The most abundant transcripts for each cluster are listed in Table S7. Noticeable is the fact that most of the differentially regulated genes (55%) in our experiments have no previously assigned function.

For overall view of the processes and functions altered during the early and late induction of MFA, classification of the differentially expressed genes was performed using the Gene Ontology (GO) database. GO accessions were further assigned to the differentially expressed genes based on their sequence similarities to known proteins in the UniProt database annotated with GO accessions as well as InterPro and Pfam domains they contain. Of the 10,513 annotated isotigs, 6,431 were assigned at least one GO term (Table 1). Several GO classifications were over-represented in genes that had increased or decreased transcript accumulation during early and/or late-induction of MFA. The GO terms ‘Metabolic process’, ‘Catalytic activity’, and ‘Membrane’ were most represented among the biological process (Figure S3), molecular function (Figure S4), and cellular component (Figure S5) categories, respectively. Most terms showed a higher number of up-regulated than down-regulated genes during early induction of MFA, indicating that the strong trend towards gene up-regulation was distributed among the different categories. The up-regulated group during early induction of MFA with the highest number of the differentially expressed genes was ‘Metabolic process’, ‘Oxidation reduction’, ‘Protein amino acid phosphorylation’, ‘Carbohydrate metabolic process’, ‘Transmembrane transport’, and ‘Regulation of transcription’ (Figure S3A).

### Characterization of Cell-wall-related Genes Associated with Early and Late Induction of MFA

**Figure 3:**
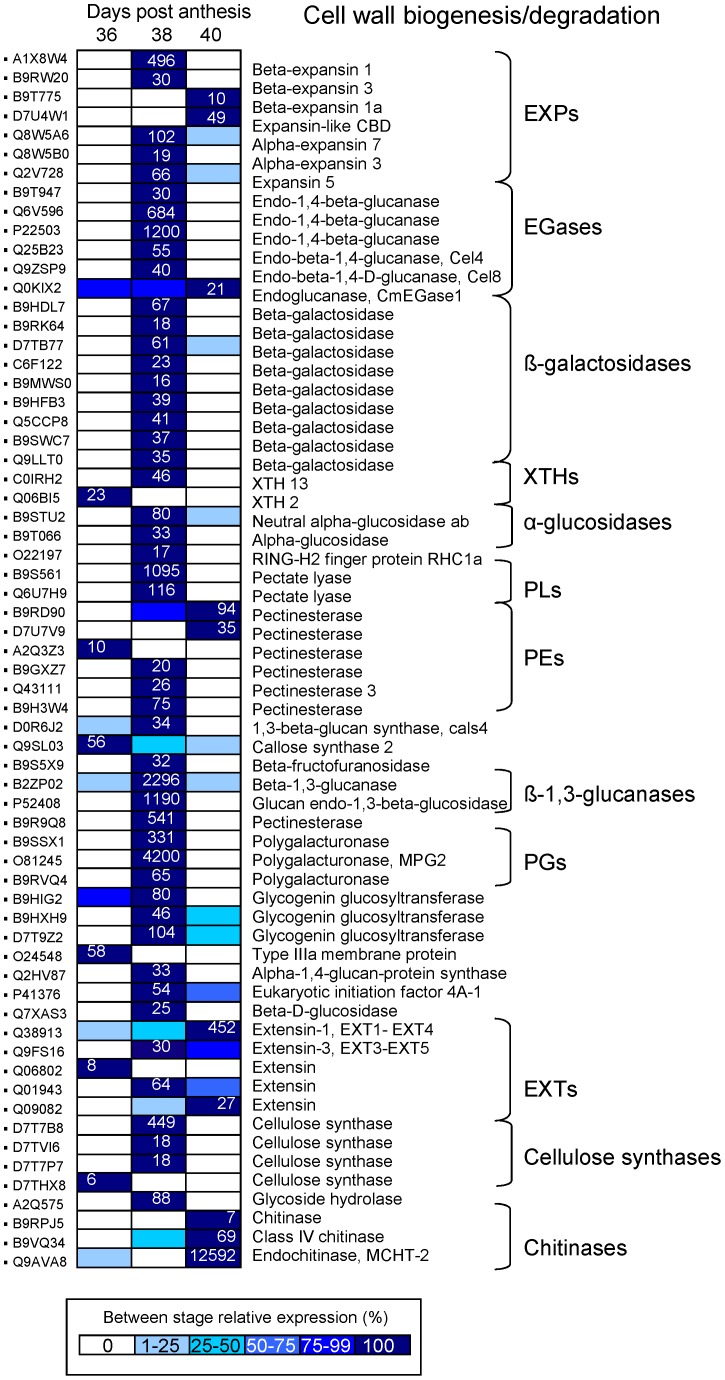
Expression profile of family genes encoding various cell-wall proteins during melon MFA. Sequences were selected after establishing a P<0.01. Gene-expression levels at 36, 38, and 40 DAP are indicated with colored bars. For the sample displaying maximal expression level, the normalized transcript abundance is expressed as the number of transcripts per total transcripts. For the other sample, expression level is indicated as percentages of the maximal normalized transcript abundance of the gene, as described in the color code from 0% (white) to 100% (dark blue). Additional information on the cell-wall-related genes is presented in Tables S8 and S9.

Previous investigations using microarrays and differential screening techniques have identified AZ-specific or AZ-enriched genes related to cell-wall rearrangements [23], [25], [26]. Analysis of our current data set confirms the abscission-induced accumulation of transcripts putatively involved in changing cell-wall composition and properties, including 79 differentially expressed genes that encode proteins with probable functions in cell-wall remodeling during melon MFA (Fig. 3; Tables S8, S9). Of cell-wall-related differentially expressed genes, 52 (three polygalacturonases (PGs), two pectinesterases (PEs), one β-fructofuranosidase, two α-expansins (α-EXPs), three β-expansins (β-EXPs), one chitinase, one β-1,3-glucanase, five endo-β-1,4-glucanases (EGases or Cels), one glucan endo-1,3-β-glucosidase, one xyloglucan endotransglucosylase/hydrolase (XTH), two pectate lyases (PLs), nine β-galactosidases, and other proteins) were up-regulated exclusively during early induction of MFA, and six (two PEs, one extensin (EXT), one chitinase, and two β-EXPs) were up-regulated exclusively during late induction of MFA, whereas only two genes were up-regulated during both the early and late induction of MFA. Thus, our approach provided results that corroborate, and expand on, previous experiments identifying pathways of cell-wall synthesis and breakdown as being induced during abscission of other plant organs such as immature fruits, floral, and leaf [23], [25], [27].

In cluster A, the most abundant transcripts encode XTH2 and callose synthase 2 (CALS2). As XTHs are involved in the modification of the load-bearing cell-wall components, *XTHs* were typically up-regulated during the abscission process in soybean leaf, arabidopsis stamen, citrus leaf, tomato flower, and rose petal [25], [27], [28], [29], [30]. Our results showed that 2 XTHs, i.e., *CmXTH2* and *CmXTH13*, are down- and up-regulated, respectively, during early induction of MFA, suggesting that the role of CmXTH2 in mature-fruit AZ could be related to the maintenance of the structural integrity of the cell-wall, and the decrease in *CmXTH2* expression during early induction of melon MFA may contribute to cell-wall loosening, which is regulated through different *XTH* genes, such as *CmXHT13*. On the other hand, *CmXTH13* expression is strongly down-regulated during late induction of MFA in melon-AZ, suggesting that CmXTH13 action may not be important for wall restructuring after AZ-cell separation. Expression of other melon *XTH* genes (*CmXTH1* and *CmXTH3*) has been previously shown to be ripening-associated, but is only partially ethylene-dependent, suggesting that they do not play a significant role in ethylene-dependent melon-fruit softening [31]. Previously, *XTH2* has been identified as XTH under brassinosteroid (BR) control, whereas *XTH13* has been previously identified as XTH under combined auxin and BR control during low-blue induced shade-avoidance responses of arabidopsis seedlings [32]. On the base of homology, we thus hypothesize that auxin and BR may regulate *CmXTH13* during melon MFA, by which they could regulate cell-wall organization in AZ. Thus, these results indicate that the two *XHT* genes have diversified their expression profile within the fruit-AZ in such a way as to take responsibility for particular roles in the cell-wall dynamics.

At 38 DPA, three different *PG* genes are induced in melon-AZ during early induction, but late-repressed during MFA. Our analysis showed that *MPG2* expression, and not *MPG1*, is up- and down-regulated in melon-AZ during early and late induction of MFA, respectively, suggesting that *MPG2* could play role in early loosening events influencing wall porosity. Genetic analysis has demonstrated that *ADPG1* and *ADPG2* are essential for silique dehiscence in arabidopsis [33]. In addition, ADPG2 contributes to floral organ abscission, while ADPG1 and ADPG2 genes contribute to anther dehiscence [33]. Our data also show that 2 PL, and 3 PE genes were strongly up-regulated during early induction of MFA, suggesting that these types of enzymes may also contribute to wall loosening associated with melon MFA, as previously indicated in stamen, leaf, and petal abscission [27], [29], [30]. Similarly, our results indicate that 5 EXP (*EXP5*, *β-EXP1*, *β-EXP3*, *α-EXP3*, *α-EXP7*) genes were up-regulated during early induction of abscission, as previously demonstrated in the abscission process [28], [34]. These results raise the possibility that several EXPs may act in cell enlargement during early induction of melon MFA. On the other hand, two cell-wall-related genes, *EXT1-EXT4* and *EGase1*, were found in our analysis to be up-regulated specifically in melon-AZ during both the early and late induction of MFA. The *EXT1-EXT4* gene is induced by wounding, and by a range of stimuli such as abscisic acid (ABA), jasmonic-acid (JA), and salicylic-acid (SA) [35].

Notably, in our analysis, a novel cell-wall-related gene, *EXORDIUM (EXO) like 2* (*EL2*), was found to be up-regulated in melon-AZ during early induction of MFA. This extracellular *EXO* gene was identified as a potential mediator of BR-promoted growth [36], and it is part of a gene family with eight members in arabidopsis. As EXO is presumably involved in a signaling process which coordinates BR-responses with environmental or developmental signals, it cannot be ruled out that EXO protein might be involved in cell enlargement associated with the early induction of melon MFA. Recently, other gene under control of BR, *XET-BR1*, has been found to be involved in tomato flower abscission [25]. Thus, here, we found strong up-regulation of *EXO* in melon-AZ, which could likely to act downstream of the BR signaling pathway in melon-AZ, and may mediate MFA via modifications of cell-wall properties and metabolism.

Profile cluster C includes transcripts accumulating in AZ sample at 40 DPA, such as one PE, two chitinases, two β-EXPs, and two EXTs, indicating that these types of enzymes may be required for complete cell separation of mature-fruit, and possibly for wall restructuring after AZ -cell separation. Accumulation of chitinases has been associated with plant defense [37], [27]. In the present work, AZ pyrosequencing analysis revealed that out of 4 genes encoding chitinases analyzed, expression of 3 chitinase genes are up-regulated in melon-AZ during MFA (1 early and 2 late induction), while expression of only one endochitinase (*MCHT-2*) is down-regulated and up-regulated during early and late induction of MFA, respectively, indicating that MCHT-2 act exclusively in late events throughout cell separation in melon. Thus, our study indicates that all members of the PG, PL, EGase, β-1,3-glucanase, β-glucosidase, and β-galactosidase families were strongly up-regulated exclusively during early induction of MFA, suggesting that these types of enzymes act synergistically in early loosening events to remodel the cell-wall, while expression of members of the PE, EXP, α-glucosidase, and EXT families proved to be up-regulated during early induction and remained steady or became highest throughout cell separation, implying that these types of enzymes have the potential for dual effects on loosening and cell separation.

### Vesicle Trafficking is Related to Early Induction of Melon MFA

**Figure 4:**
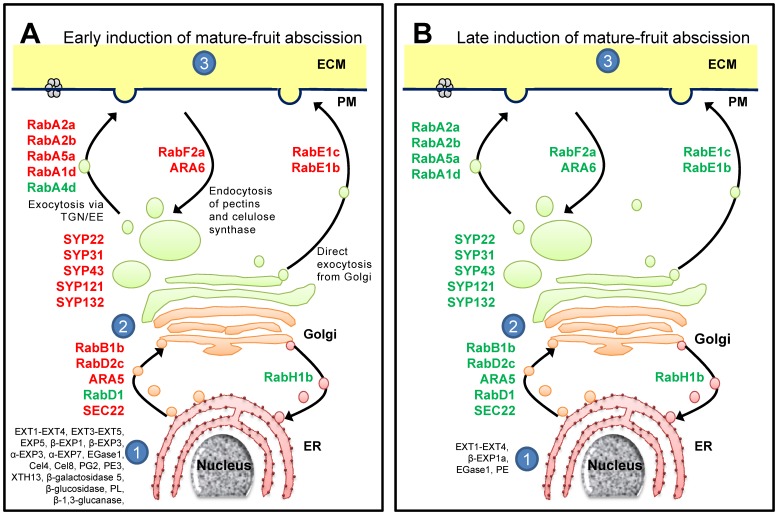
Simplified schematic representation of the trafficking pathways to and from the cell wall during early (A) and late (B) induction of melon MFA. The Rab-GTPases probably involved at each step are indicated in green (down-regulated) and red (up-regulated). 1, Synthesis of proteins in endoplasmic reticulum (ER): EXT, EXP, EGase, PG, PE, PL, XTH, β-glucosidase, β-galactosidase, and β-1,3-glucanase during early induction of MFA; EXT, EXP, EGase, and PE during late induction of MFA. 2, Synthesis of matrix polysaccharides and assembly of proteins in Golgi and TGN/EE (the *trans*-Golgi network and early endosomal compartments). 3, Modification of wall elements by secreted enzymes. Pathways to and from the vacuole have been omitted for simplicity. Additional information on the vesicle-trafficking-related genes is presented in Table S10. (ECM: equivalent to the “cell wall” or “apoplast”; PM: plasma membrane).

Gene expression up-regulated in melon-AZ indicates cell-wall rearrangements between 38 and 40 DPA. Changes in deposition of wall material require vesicle formation and transport, reflected by a large number of up-regulated genes at 38 DPA (Table S10). Kinesin motors, Rab-GTPases together with tubulins are potentially involved in vesicles and organelle transport [38]. In particular, Rab-GTPases have been shown to be important regulators of the endomembrane traffic, mediating communication between vacuole, plasma membrane, ER, Golgi, and cell-wall [39]. However, little is known about Rab-GTPases participating in abscission. Here, among Rab-GTPases identified in our analysis, 22 Rab-GTPases were up-regulated at 38 DAP in melon-AZ (Table S10), indicating that at least some members of Rab-GTPases play major roles in secretion and/or recycling of cell-wall components during early induction of melon MFA. 8 Rab11 (corresponding to the arabidopsis RabA clade), 3 Rab2 (corresponding to RabB), 2 Rab18 (corresponding to RabC), 3 Rab1 (corresponding to RabD), 2 Rab8 (corresponding to RabE), 2 Rab5 (corresponding to RabF), 2 Rab7 (corresponding to RabG) proteins, and one Rab6 (corresponding to RabH) protein from melon are expressed strongly in fruit-AZ at 38 DPA (Fig. 4). Thus, they are diferent classes of Rab-GTPase protein and would, therefore be expected to regulate either exocytosis from the trans-Golgi network, transport to the plasma membrane and the cell-wall, endocytosis or vacuolar trafficking during melon MFA (Fig. 4). This implies their involvement in mediating delivery to the apoplast of cell-wall-depolymerizing enzymes required for cell expansion and cell-wall loosening at 38 DPA in melon-AZ. Notably, the synthaxin SYP121 (t-SNARE family), which has been related to ABA-responsive secretion [40], is also up-regulated during early induction of MFA. On this basis, it might be asked whether this SYP121 protein plays an essential part together with some of the different up-regulated Rab-GTPases in melon AZ for the transport of proteins and membrane through the endomembrane system to their destination, and whether this transport plays a critical role in mediating plant-hormone signals during melon MFA. A gene that encodes an ADP-ribosylation factor GTPase-activating protein (ARF-GAP), NEVERSHED (NEV), has been proposed to regulate the movement of proteins essential for activating cell separation in arabidopsis [41]. Our results demonstrate that some members of ARFs were up-regulated during early induction of MFA (Table S10), strengthening the possibility that ARF GTPase-regulated endosome trafficking can play a role for abscission signaling. Of particular interest is also the set of two members of RAN GTPases family homologous to RAN3 [42]. The up-regulation of these genes in fruit-AZ at 38 or 40 DPA suggests that they may function in mediation of nucleocytoplasmic transport during abscission signaling. It bears noting that the overexpression of *TaRAN1* and *OsRAN2* renders arabidopsis hypersensitive to auxin and ABA, respectively [43], [44], and thus these two signaling pathways might be particularly sensitive to perturbing components of nucleocytoplasmic trafficking. Other up-regulated gene homologous to MIRO1, encodes a member of Rho GTPase family that have evolved to regulate mitochondrial trafficking [45]. Therefore, these results raise the possibility that several families of small-GTPases may act in vesicle trafficking during MFA, including Rabs, ARFs, RANs and Rhos. Other genes were noticeably present in fruit-AZ at 38 DPA and involved vesicle trafficking, encode V-type ATPases, and midasins, which were involved in trafficking from the trans-Golgi Network to the central vacuole. Thus, these data suggest that vesicular trafficking may regulate cell separation during early induction of melon MFA.

A model integrating the potential roles of membrane trafficking and receptor-like kinase (RLK) signaling during organ separation has been recently presented in arabidopsis floral abscission [46]. In arabidopsis, floral abscission is controlled by the competing activities of several leucine-rich repeat receptor-like protein kinases (LRR-RLKs) [46], [47], [48]. Here, expression analysis detects several LRR-RLKs and RLKs up-regulated and down-regulated at 38 or 40 DPA in melon-AZ (Table S11). These results potentially signify the involvement of several RLKs in regulating melon MFA. In addition, a subset of 181 genes encoding phosphoproteins was defined as being significantly changed in abundance during melon MFA (Table S11). Kinase motifs in abscission-responsive proteins matched to casein kinases, shaggy-related protein kinase, CBL-interacting protein kinase, CBL-interacting serine/threonine-protein kinase (CIPK), calcium-dependent protein kinase (CDPK, CPK), mitogen-activated protein (MAP) kinase (MPK), wall-associated kinase (WAK), and S-locus-like receptor protein kinase, indicating a possible critical function of these kinase classes in the regulation of cell separation during melon MFA. Expression of casein kinase genes was preferentially detected in laminar AZ cells in citrus leaves during ethylene-promoted abscission [27]. Other phosphoproteins that mediate cells communicate and perceive signals via cell-walls, such as MAPK, WAK, and leucine rich receptor kinase, are up-regulated at 38 DPA suggesting that they may have regulatory functions in macromolecular trafficking between cell-wall and membrane in fruit-AZ during early induction of melon MFA. A previous study has shown that some MPKK or MEK proteins, MKK4 and MKK5, are involved during the cell separation phase of the floral abscission process in arabidopsis, but are not required for the AZ formation [47].

On the other hand, our results also appear to reflect differences in AZ transport during early and late induction of MFA (Figure S6); for example, the early transport results mainly accomplished by the gene activity of boron and nitrate transporters and the late transport by phosphate transporter, whereas the transport of sodium and calcium are associated with both early and late induction of melon MFA.

### Characterization of Plant-hormone-related Genes Associated with Early and Late Induction of MFA

**Figure 5:**
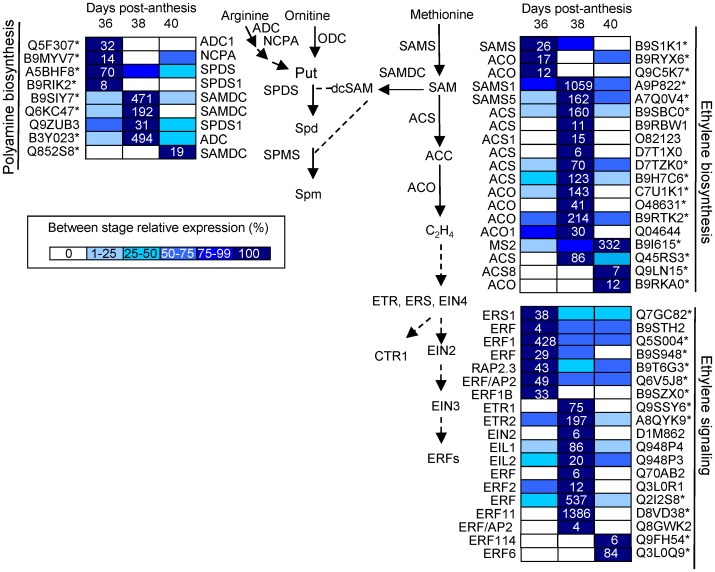
Expression profiling of genes related to ethylene biosynthesis and signaling, and polyamine biosynthesis as reconstructed from the 454 pyrosequencing transcriptome. UniProt IDs followed by asterisks indicate transcripts showing significant variations during abscission (P<0.01, group I). Gene-expression levels at 36, 38, and 40 DAP are indicated with colored bars. For the sample displaying maximal expression level, the normalized transcript abundance, expressed as the number of transcripts per total transcripts. For the other sample, expression level is indicated as percentages of the maximal normalized transcript abundance of the gene, as described in the color code from 0% (white) to 100% (dark blue). Additional information on the hormone-related genes is presented in Table S12.

**Figure 6:**
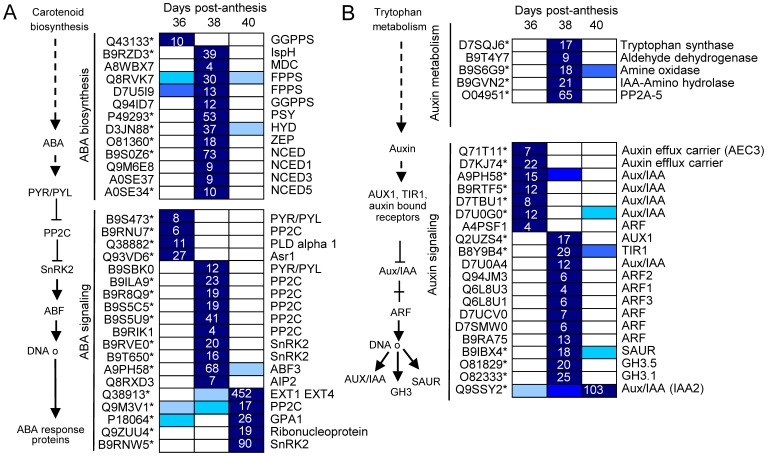
Expression profiling of genes related to ABA (A) and auxin (B) biosynthesis and signaling in fruit-AZ during melon MFA. UniProt IDs followed by asterisks indicate transcripts showing significant variations during abscission (P<0.01, group I). Relative expression is as in Figure 5. Additional information on the hormone-related genes is presented in Table S13.

**Figure 7:**
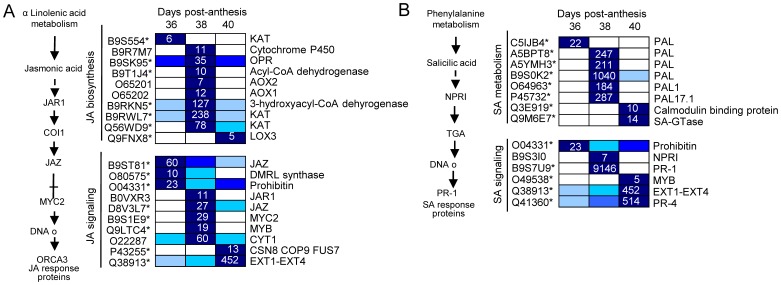
Expression profiling of genes related to JA (A) and SA (B) metabolism and signaling in fruit-AZ during melon MFA. UniProt IDs followed by asterisks indicate transcripts showing significant variations during abscission (P<0.01, group I). Relative expression is as in Figure 5. Additional information on the hormone-related genes is presented in Table S14.

**Figure 8:**
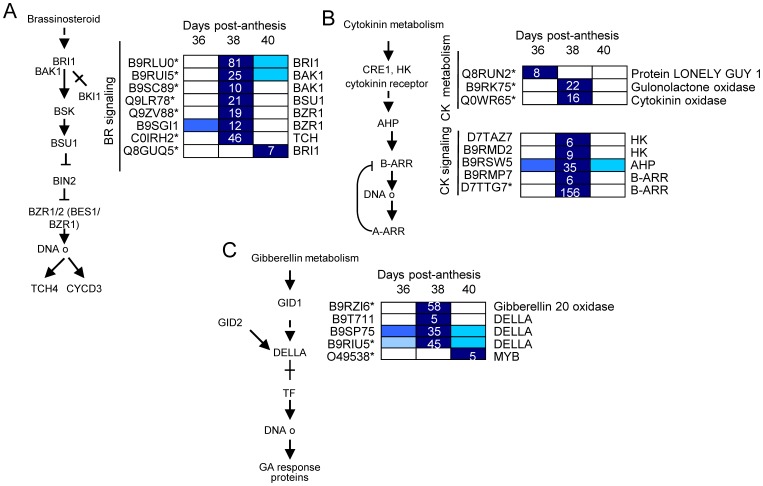
Expression profiling of genes related to BR (A), CK (B), and GA (C) biosynthesis and signaling in fruit-AZ during melon MFA. UniProt IDs followed by asterisks indicate transcripts showing significant variations during abscission (P<0.01, group I). Relative expression is as in Figure 5. Additional information on the hormone-related genes is presented in Table S14.

Several plant-hormone pathways, such as of ethylene, auxin, ABA, and JA are involved in abscission processes [26]. In our study, among the 116 differentially expressed genes (P<0.01, group I) related to plant-hormone metabolism and signaling (Tables S12,S13,S14), those related to ethylene (28 genes) and ABA (23 genes) were the most represented, followed by those related to auxin (16 genes), JA (15 genes), and SA (12 genes). Few genes related to polyamine (8 genes), BR (7 genes), cytokinine (CK, 4 genes), or gibberellin (GA, 3 genes) were found. Expression patterns within these pathways were then analyzed by hierarchical clustering, and these pathways are shown in Figs. 5,6,7,8. Differential expression is indicated by a color scale that reflects the coefficient of variation. The resulting colored pathway figures indicate putative nodes of transcriptional regulation across the profiles of the three samples.

Both the early and late induction of MFA in melon-AZ are apparently characterized by an active ethylene as well as ABA biosynthesis and signaling, although expression of far fewer ethylene- or ABA-responsive genes was affected by late induction compared with early induction of MFA. In particular, two different S-adenosylmethionine synthase (*SAMS*) genes (*SAMS1*, *SAMS5*), four different 1-aminocyclopropane-1 carboxylic acid (ACC) synthase (*ACS*) genes (Figure S7), three different ACC oxidase (*ACO*) genes (Figure S8), one ETR1 gene, one ETR2 gene, and two diferent ERF genes (*ERF*, *ERF11*) were up-regulated during early induction of abscission (group I, cluster B), while one methionine synthase 2 gene, one *ACS* gene, two *ACO* gene, and two *ERF* genes (*ERF 6*, *ERF114*) were up-regulated during late induction of abscission (group I, cluster C) (Fig. 5). Thus, it seems that ETR1, ETR2, and ERF11 are involved in early induction of MFA, while ERF6 and ERF114 are involved in late induction of melon MFA.

Our pyrosequencing-based approach enabled the identification of 24 sequences that contained at minimum the AP2 domain, and were retained for further phylogenetic analysis (Figure S9). Based on its expression pattern, which was high in the AZ at partial cell separation, *ERF11* can be considered as a good candidate for encoding an ERF involved in MFA regulation. By contrast, other genes related to ethylene signaling, such as *EIL2* (EIN3*-like, EIL*), have recently been found to be involved in olive MFA [22], while *ETR4* and *CTR1* have been demonstrated to participate in the late stages of tomato flower abscission [23]. In this study, the up-regulation of genes encoding ethylene biosynthesis and signaling components prior to the complete cell separaton is consistent with previous reports that apple-fruitlet abscission is preceded by increased ethylene biosynthesis and sensitivity [34]. The down-regulation of the genes related to ethylene biosynthesis and response in the melon-AZ following abscission (Cluster A) is also found (Fig. 5): one *SAM synthase*, two *ACO*, one *ERS1*, and five *ERF* genes (*ERF1, ERF1B, ERF, ERF/RAP2.3, ERF/AP2*). Most reported AZ ERFs have been shown to be induced with abscission, or ethylene treatment, including from citrus *ERF1* and *ERF2* [27], apple *ERF* [23], and tomato *ERF1b, ERF1c, ERF2*, and *ERF3* [25]. However, the decline in melon ERF1 and ERF1B is likely to be associated with early induction of melon MFA.

In addition, genes encoding key genes related to polyamine biosynthesis, including *ADC*, *NCPA*, *SAMDC*, and *SPDS*, showed temporal regulation across the samples studied (Fig. 5). The down-regulation of one *ADC1*, one *NCPA*, and two different *SPDS* genes at 38 DPA in melon-AZ agrees with results from apple during shading-induced and NAA-induced fruit abscission [34]. However, other PA-related genes, such as one *ADC* and three *SAMDC*, were up-regulated following melon MFA, in agreement with data of the ADC activity from olive during MFA [49], although in olive, a decline of *SAMDC* expression was found during MFA [50].

Melon-AZ undergoes a large increase simultaneously in ethylene- and ABA-related expression during MFA, suggesting regulatory functions and/or interactions for these two hormones in the melon-AZ. It has been proposed that ABA might sense nutrient stress and thus could be correlated with the ethylene-associated abscission activation in citrus fruitlets [51], and with shading-induced abscission of apple fruits [34]. In our study, of the 8 differentially expressed genes involved in ABA biosynthesis, six genes show increased transcript abundance during early induction of melon MFA, indicating that ABA levels probably rise at 38 DPA in AZ (Fig. 6A). Furthermore, this marked up-regulation of ABA biosynthetic genes is followed by a subsequent decline in transcripts. At same time, an ABC transporter of the G class transporting ABA [52], *ABCG25,* an ABA exporter through the plasma membrane, is up-regulated at 38 DPA, while *ABCG40*, an ABA importer, is up-regulated at 40 DPA (Figure S6), suggesting the active control of ABA transport between AZ-cells during melon MFA. The metabolism of ABA, therefore, clearly displayed a coordinated transcriptional activation at abscission in the melon-AZ.

The present analysis also found components of ABA signaling induced during early and/or late induction of MFA. Four *PP2C* (type 2C protein phosphatase), two *SnRK2* (subfamily 2 of SNF1-related kinases), and one *ABF3* (AREB/ABF subfamily transcriptional factor) were exclusively up-regulated during early induction, whereas only one *PP2C* was exclusively up-regulated during late induction of melon MFA (Fig. 6A). In addition, down-regulation of ABA-related genes was found following MFA. One *PYR/PYL* (ABA receptor) and one *PP2C* were down-regulated during early induction, whereas four *PP2C*, two *SnRK2* and one *ABF3* were down-regulated in melon AZ during late induction of abscission. These differences in ABA-related responses suggest that the ABA signaling is more active in the early induction that in late induction of melon MFA. Additionally, only one *SnRK2* was strongly up-regulated during both early and late induction of melon MFA, indicating that this SnRK2 may be commonly needed for full activation of AREB/ABF transcription factors (TFs) required for complete cell separation during melon MFA.

Given the importance of auxin-mediated processes in abscission [53], more research is needed to elucidate the mode of action of auxin signaling in the AZ. A divergence in auxin-related gene expression was noted for early vs. late induction of MFA. Only one auxin-related gene (*IAA2*) showed up-regulation during late induction, while 8 genes were induced during early induction of melon MFA (Fig. 6B). The auxin-amino acid hydrolase gene, which is involved in auxin homeostasis, was up-regulated and down-regulated during early and late induction of MFA, respectively. Auxin-amino acid hydrolase may provide for local concentrations of auxin within the AZ to promote cell enlargement prior to complete cell separation. This is consistent with other studies on abscission, in which genes encoding for protein homologs of this family were found to be up-regulated after flower removal [25] and NAA-induced apple fruit abscission [34]. However, transcript levels for the auxin conjugating enzymes, *GH3.5* and *GH3.1* were also found to be induced during early induction of melon MFA. Furtermore, these *GH3.5* and *GH3.1* genes show down-regulated expression in the melon AZ during late induction of MFA.

Several transcripts related to auxin transport and perception also displayed increases during early induction of melon MFA. Earlier reports indicated that the ethylene burst preceding abscission of apple fruitlets may be responsible for the lower transcript level of an auxin efflux carrier PIN1 in seed, leading to a reduced auxin export and the induction of apple fruitlets abscission [34]. In our study, transcript levels for two auxin efflux carriers (AEC3, AEC) lowered at the early induction of MFA, indicanting that the decline in expression may be related to a reduced rate of basipetal auxin transport in melon AZ, and could signal the beginning of abscission process, which may be associated with increases in expression of ethylene-related genes. However, a transcript encoding auxin influx carrier-like protein 3 (AUX1/AIC3) increased during early induction of melon MFA, suggesting a role regulating auxin influx and maintaining auxin sink-strength in this tissue in a similar manner to its arabidopsis and tomato orthologs, *AtLAX3* and *SlLAX3*, which have been shown to create cell-specific auxin sinks [54], [55]. In addition, one auxin receptor *TIR1* gene (TIR1/AFB, a nuclear receptor) was up-regulated during early induction of MFA. Our data indicate that there are numerous transcript responses to auxin. The three families of early auxin responsive genes, *Aux/IAA* (*IAA2*), *GH3* (*GH3.5* and *GH3.1*), and *SAUR*, which contain a binding motif to the ARF TF, were up-regulated during early induction of melon MFA. Only an Aux/IAA gene, *IAA2*, showed increased expression in melon AZ during both the early and late induction of abscission, suggesting that *IAA2* is likely to be associated with MFA, and may be required for complete cell separation during MFA in melon AZ. In contrast, *IAA1* and *IAA2* genes were down-regulated by deblading/decapitation in *Mirabilis jalapa,* demonstrating a correlation between acquisition of competence to respond to ethylene in both leaf and stem AZs, and a decline in the abundance of auxin regulatory gene transcripts [25]. Additionally, we detected numerous auxin-regulated genes belonging to the Aux/IAAs, which were down-regulated during early induction of abscission. In tomato flower AZ, the diminished expression of these *Aux/IAA* genes is as a result of auxin depletion following flower removal, and is neither AZ specific nor affected by ethylene [24]. In this study, the modification of auxin sensitivity by down-regulation of auxin response regulators, such as *Aux/IAAs*, can lead to MFA.

Early induction of melon MFA also appeared mainly to up-regulate expression of JA- and SA-related genes (Fig. 7). Five out of the 7 differentially expressed genes involved in JA biosynthesis, were up-regulated in melon AZ during early induction, as has been described in citrus leaf abscission [27] and arabidopsis stamen abscission [29]. Similarly, up-regulation of two *JAZ* genes and one *MYC2* gene occurs in melon-AZ during MFA, suggesting an increase of JA levels and sensitivity in mediating melon MFA. Also, several genes related to SA were up-regulated during MFA, such as *PAL*, *NPR1*, *PR-1* and *PR-4*. The gene-profiling data showed that *PR1* and *PR4* transcripts, which are markers for the SA response [56], were increased during early induction of melon MFA, whereas an *UDP-glucose:SA glucosyltransferase* transcript, which is involved in SA conjugation, increased during late induction of melon MFA.

Up-regulation of BR signaling was unexpected during melon MFA (Fig. 8A). Although BRs are essential growth regulators, involved in many physiological processes [57], their role in abscission regulation remains to be determined, because information regarding the genes controlling BR signaling in AZ during abscission is extremely limited. The present work provides the first report available showing up-regulation of the main components of BR signaling during abscission, with particular emphasis on early induction of MFA. Here, we show that a *BRI1* receptor kinase, 2 *BAK1* co-receptors, a *BSU1* phosphatase, and a *BES1/BZR1* TF (BES1/BZR1 homolog protein 4, BEH4 At1g78700) were exclusively up-regulated during early induction of melon MFA, while only a different receptor *BRI1* gene was exclusively up-regulated during late induction of MFA, suggesting that the up-regulation of receptor *BRI1* may be required for complete cell separation during MFA. Thus, the temporal distribution of *BRI1*, *BAK1*, *BSU1*, and *BEH4* expression indicates that their coordinated action regulates BR response in melon-AZ during MFA.

Recently, it has been demonstrated that PP2A (cytoplasmic protein phosphatase 2A) is responsible for dephosphorylating BZR1 and BZR2/BES1, thus increasing the active form of these TFs and promoting BR signaling [58]. It bears noting that we found the up-regulation of the expression of two *PP2A* genes (*PP2AA2DF1*/At3g25800, *PP2A5*/At1g69960), and one *BEH4-like* (BZR1) gene in melon-AZ at 38 DPA, suggesting that the up-regulation of *PP2A* gene activates BR-responsive gene expression in melon-AZ and MFA by dephosphorylating BEH4. In addition to BR, recent experiments indicate that PP2A also regulates ethylene biosynthesis by differentially regulating the turnover of ACS isoforms [59], which suggests that PP2A phosphatase may be involved in the abscission process. Thus, these data imply that ethylene biosynthesis and BR signaling converge at the transcriptional level to synergistically activate melon MFA.

In the case of GA, our data suggest that GA signaling is negatively regulated by GAI (DELLA protein) during melon MFA (Fig. 8), a finding consistent with data from apple during immature-fruit abscission [23] and during shading- and NAA-induced abscission [34]. However, the up-regulation of GA biosynthesis during melon MFA contrasts with data from apple immature-fruit abscission [23], suggesting that a low GA level in immature-fruit-ZA, and a high GA level in mature-fruit-ZA may be associated with abscission. Thus, our study indicates that besides the participation of ethylene, ABA, JA and auxin in controlling abscission events, other hormones, such as SA and BR, apparently participate in an intricate interaction web regulating the early induction of melon MFA. A representative KEGG map for plant-hormones signalings during melon MFA is given in Figure S10.

### Identifying Transcription-factors Critical for Early and Late Induction of MFA

**Figure 9:**
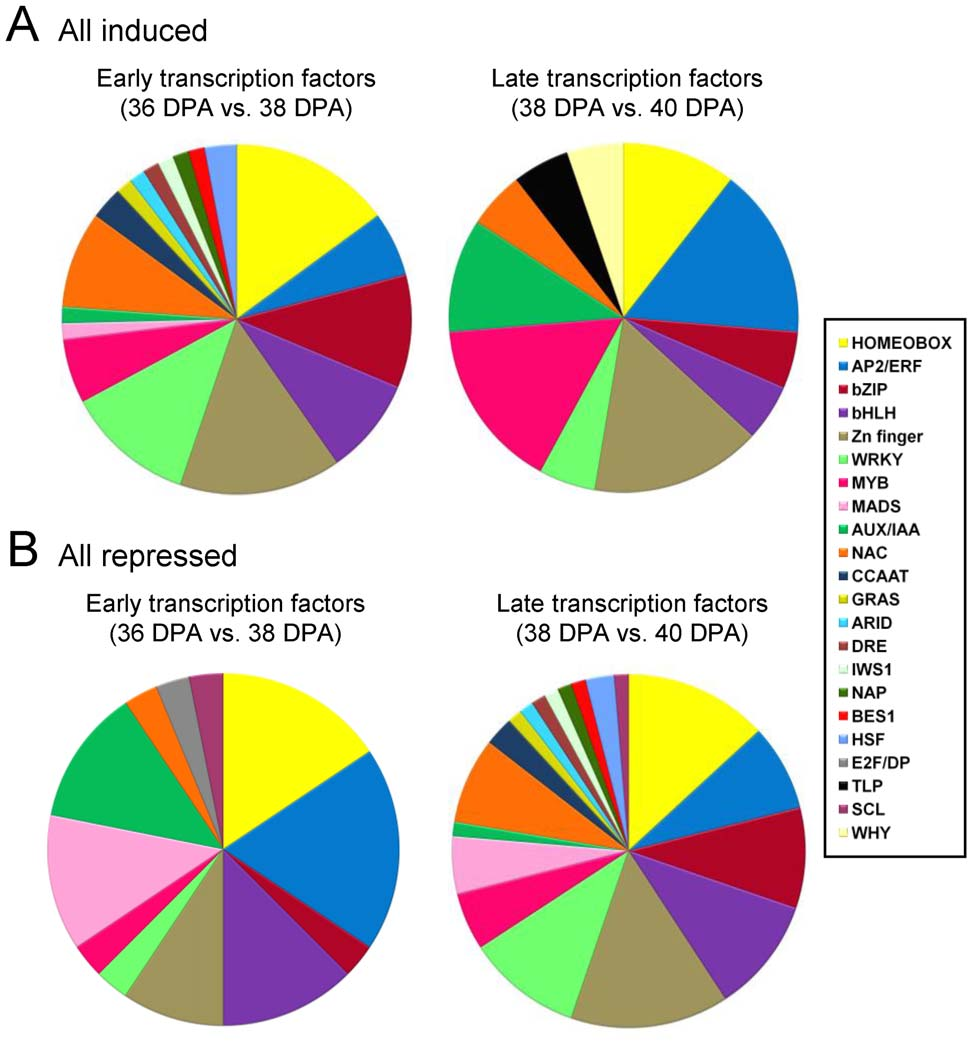
Melon TFs induced (A) or repressed (B) in fruit-AZ during early and late induction of melon MFA. Additional information on the TF-related genes is presented in Table S15.

Of 4,801 differentially expressed genes, 123 genes putatively encoding TFs of diverse families were differentially expressed in the fruit-AZ (P<0.01), most of them with an up-regulation pattern from 36 to 38 DPA (Table S15). Overall, there were 36 genes with peak read amounts within cluster A, 73 genes at 38 DAP within cluster B, and 15 genes at 40 DAP within cluster C. Within cluster A, with peak transcription amounts at 36 DAP, the most abundant TF the most abundant TFs was a NAC domain protein found within subcluster A3 (Table S15). Notably, there was one AP2/ERF protein (ERF1) that was abundant within the subcluster A2 (Table S15). Indeed, AP2/ERF proteins were the most represented class of proteins at 36 DAP, one within subcluster A1, four within subcluster A2, and one within subcluster A3, suggesting coordinated regulation of this class of TFs. MADS-box proteins were the second most represented class within the subcluster A2 (MADS2, ERAF17 and Flowering locus C/FLC). Our pyrosequencing-based approach identified 7 sequences that contained the conserved MADS domain, and that were retained for further phylogenetic analysis (Figure S11). In cluster B, all transcripts for TFs were found in subcluster B1 (Table S15), suggesting that tight transcriptional coordination occurs at the early induction of melon MFA. The classes that are well represented in cluster B included 10 Zinc finger (ZF) proteins and 10 homeobox domain proteins. Remarkably, only 15 TFs were found within cluster C, suggesting that restricted transcriptome regulation occurs at late induction of melon MFA (Table S15). In cluster C, 14 out of 15 genes were found within subgroup C1. The most abundant TFs were two ZF proteins, and one AP2/ERF protein (ERF6) found within subcluster C1 (Table S15). In addition, one auxin-induced repressor protein (IAA2) was included in subcluster C2. Finally, no genes for subcluster C3 were found within group I. Thus, most members of MADS box, and Aux/IAA families were found within cluster A, and were down-regulated genes during melon MFA, whereas most members of ZF, WRKY, basic lecine zipper (bZIP), and NAC families were found within cluster B or C, and were up-regulated genes during MFA (Fig. 9, Table S15).

When we considered all of the 2,209 induced and 2,592 repressed genes in our dataset, we obtained 94 up-regulated and 89 down-regulated TF genes during MFA in our two experiments: 67 TF genes (33%) were induced at 38 DPA, and repressed at 40 DPA (early up-regulated TFs), six (3%) were repressed at 38 DPA and induced at 40 DPA (late up-regulated TFs), one (0.5%) was induced at both 38 DPA and 40 DPA, and 13 (6.5%) were repressed at both 37 DPA and 39 DPA (Table S15). Therefore, only a few up- or down-regulated genes occurred in both early and late induction, thereby suggesting that time-specific events occur, and that the most genes are strongly up-regulated in fruit-AZ during early induction of melon MFA.

Among the early up-regulated TFs, HSF proteins, CCAAT-binding proteins, ARID protein, DRE protein, Nut2 protein, NAP protein, BES1/BZR1 (bHLH protein, BEH4), and IWS1 protein have not previously been ascribed functions in abscission (Table S15). The largest early-upregulated TF families were the ZF (10 genes), homeobox (10 genes), WRKY (8 genes), and bZIP (7 genes), impliying that TFs from these families could be involved in triggering the transcriptional cascade during MFA. However, within the set of 107 known or predicted TFs induced or repressed during late induction of melon MFA, we found that only 6 out of 20 genes exclusively induced at 40 DPA were repressed at 38 DPA. We believe that the 6 induced genes, one AP2/ERF (RAP2.3), one ZF (C2H2L24), one Aux/IAA, one NAC (NAC-A/B) and two homeobox proteins, are strong candidates for possible roles in regulating pathways that are activated specifically during late events of melon MFA.

Our analyses of TFs also revealed some similarities, as AP2/ERF, MYB, ZF, WRKY, and NAC proteins, with previous studies in immature-fruit abscission [23], [53], and provide insights into the regulatory processes that occur during MFA. In addition, this study reveals greater resolution of these events. Thus, for cluster A, enriched in the AP2/ERF, Aux/IAA, and MADS-box TF families, it can be seen that these TFs are abundant at 36 DPA in fruit-AZ, and decrease during early induction of melon MFA, but increase, decrease, or remain largely unchanged during late induction of MFA. By contrast, the transient cluster B is enriched in bZIP, homeobox, WRKY, and ZF proteins, while cluster C is enriched in the MYB family. Therefore, despite all clusters contain members from several TF families, there is a clear and significant difference in the proportion of families in each cluster, implying an important time-specific regulatory requirement for the expression of these TFs.

An equivalent analysis of the down-regulated TFs genes identified in our two experiments, gave a very different result. Whereas 78% (73 of 94) of the total up-regulated TFs showed increased expression during early induction of MFA, 70% (86 of 122) of the total down-regulated TFs showed decreased expression during late induction of MFA. These results appear to indicate that melon MFA causes the down-regulation of many TFs, but, in most cases, this down-regulation is not part of the endogenous program of early changes in gene-expression required for abscission. However, in our parallel analysis of down-regulated genes, most members of MADS box, and Aux/IAA families were found during early induction of MFA, suggesting that repression of this TF expression plays a role in early events during melon MFA. When we compared the down-regulated TF genes during early and late induction of abscission, we found the presence of 13 down-regulated TF, including one Aux/IAA protein, two bHLH proteins, three AP2/ERF proteins (ERF1, AP2D15, ERF), three MADS box proteins (FLC, MADS2, ERAF17) proteins, one ZF protein (SAP5), one SCL protein (SCL13) and one uncharacterized TF. All of these were common to both times in fruit-AZ during MFA, suggesting that there are common regulators of MFA between early and late induction of MFA. Thus, it seems likely that a battery of down-regulated TFs may be necessary to coordinate cell separation during melon MFA.

### Confirmation of Gene-expression Patterns Upregulated during Melon MFA

**Figure 10:**
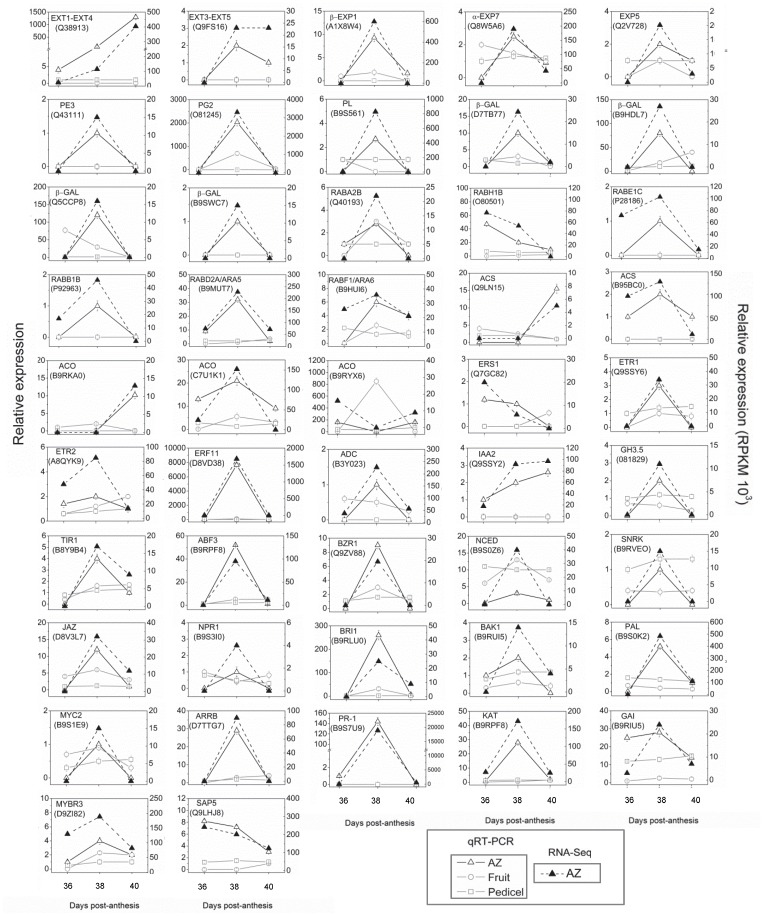
Confirmation of gene-expression patterns up or downregulated during melon MFA. qRT-PCR analysis of 47 selected genes in various melon tissues at 36, 38 and 40 DPA: AZ (-Δ-), pedicel (proximal non-AZ, -□-) and fruit mesocarp (distal non-AZ, -Ο-). Analysis of transcript levels of genes by quantitative RT-PCR. Genes and their primers are shown in Table S16. Relative expression values were normalized to the lowest expression value taken as 1. The data represent the mean values (±SEs) of duplicate experiments from three independent biological samples. Broken lines (–▴–) show expression profiling of genes in the melon AZ as reconstructed from the 454 pyrosequencing transcriptome. Broken line indicates the total read count in RPKMx1000 for each gene after normalization across the samples: AZ at 36, 38 and 40 DPA.

To verify the results of our pyro-sequencing analysis, we performed qRT-PCR analysis of the identified AZ-enriched genes during MFA induction (Fig. 10). The list of 47 selected genes and their primers are shown in Table S16. Among the abscission-associated transcripts, genes encoding key enzymes involved in cell-wall metabolism, such as *PE3* (Q43111), *β-EXP1* (A1X8W4), *α-EXP7* (Q8W5A6), *EXP5* (Q2V728), *PL* (B9S561), *PG2* (O81245) and *β-GALs* (D7TB77, B9HDL7, Q5CCP8, B9SWC7) were upregulated at 38 DPA and downregulated at 40 DPA in the AZ, which was identical to the RNA-seq results (Fig. 10). Also, we validated the increased expression of genes *EXT1-EXT4* (Q38913) and *EXT3-EXT5* (Q9FS16) at 38 DPA, as well as the increased expression of gene *EXT1-EXT4* at 40 DPA. Similarly, we confirmed the increased expression of genes encoding enzymes involved in vesicle trafficking, and phytohormone metabolism and signaling, such as *RabA2b* (Q40193), *RabB1b* (P92963), *RabE1c* (P28186), *RabD2a/ARA5* (B9MUT7), *RabF1/ARA6* (B9HUI6), one *ACS* (B95BC0), one *ACO* (C7U1K1), *ETR1* (Q9SSY6), *ETR2* (A8QYK9), *ERF11* (D8VD38), *ADC* (B3Y023), *IAA2* (Q9SSY2), *GH3.5* (081829), *TIR1* (B8Y9B4), *ABF3* (B9RPF8), *NCED* (B9S0Z6), *SnRK* (B9RVE0), *KAT* (B9RPF8), *JAZ* (D8V3L7), *MYC2* (B9S1E9), *NPR1* (B9S3I0), *PAL* (B9SOK2), *PR-1* (B9S7U9), *BRI1* (B9RLU0), *BAK1* (B9RUI5), *BZR1* (Q9ZV88), and *ARRB* (D7TTG7) at 38 DPA, as well as the downregulated genes *RabH1b* (O80501) and one *ACO* (B9RYX6) at 38 DPA in the AZ (Fig. 10). The expression level of one *ACS* (Q9LN15), one *ACO* (B9RKA0), and *IAA2* increased at 40 DPA, which was consistent with RNA-seq results. On the other hand, our results revealed downregulation of a ZF gene, *SAP5*, during MFA induction, which was identical to the RNA-seq results (Fig. 10). Thus, the qRT-PCR results were consistent with the pyro-sequencing data, indicating that the pyro-sequencing analysis results were effective.

In addition, we tested tissue specificity of these genes using qRT-PCR, which confirmed its enrichment in the AZ. We demonstrate that the expression pattern in the AZ of some genes performed by qRT-PCR is different from the pattern of expression in adjacent non-AZ tissues (pedicel or fruit mesocarp) (Fig. 10). In fact, most of the transcripts accumulated higher in the AZ, suggesting the involvement of these genes in abscission events.

In conclusion, this analysis provides novel information for the potential candidate genes and pathways associated with early induction of MFA in fleshy-fruit. At early induction of melon MFA, activated genes are related to cell-wall metabolism, endomembrane trafficking, protein phosphorylation, plant-hormone biosynthesis and signaling, and ion fluxes. Early events are potentially controlled by down-regulation of MADS-box, AP2/ERF and Aux/IAA TFs, and up-regulation of bZIP, homeobox, ZF, and WRKY TFs, while late events may be controlled mostly by up-regulation of MYB TFs during melon MFA induction. Among the early-induced TFs, HSF proteins, CCAAT-binding proteins, ARID protein, DRE protein, Nut2 protein, NAP protein, BES1/BZR1 (bHLH protein, BEH4), and IWS1 protein have not previously been ascribed functions in abscission. At present, little is known about potential AZ genes regulating MFA in fleshy-fruit. Therefore, our comprehensive gene expression profile will be very useful for elucidating gene regulatory networks of the MFA in fleshy-fruit.

## Materials and Methods

### Plant Material and RNA Isolation

Charentais melon (*C. melo* var. cantalupensis Naud, ‘Vedrantais’) flowers were tagged on the day of pollination, and the fruit-AZ samples were collected from melon fruits subsequently harvested at specified stages of MFA induction: 36 DPA, fruit-AZ pre-cell separation; 38 DPA, fruit-AZ partial cell separation; and 40 DPA, almost complete fruit-AZ cell separation and cell collapse (Fig. 1). Védrantais fruit abscission was observed at 42 DPA. The fruit-AZs, located between the pedicel and fruit (Fig. 1B), were collected from longitudinal sections by cutting 1 mm on the proximal and distal sides of the abscission fracture plane (Fig. 1C). Fig. 1D shows the tissue samples used in this study (white box). Fruit-AZ wings containing mesocarp or pedicel/calyx-like tissues were discarded. Thus, the possible contamination was reduced to a minimum level and, therefore, the data reported concern the AZ and not the fruit. Freshly excised AZ samples were immediately frozen in liquid nitrogen and stored at −80°C for RNA isolation. To examine the proximal and distal fracture planes of the fruit-AZ by scanning electron microscopy (SEM), following critical-point drying, tissues were mounted onto steel stubs, coated with gold-palladium, and observed using a LEO 1430 VP scanning electron microscope [49].

Total RNA was extracted from AZ tissues using Trizol (Invitrogen Life Technologies). RNA quality was gel verified and quantified spectrophotometrically (NanoDrop, ThermoScientific, http://www.thermofisher.com/). Messenger RNA was isolated twice with Dynabeads Oligo (dT)25 (Dynal Biotech ASA, Dynal Invitrogen, http://www.invitrogen.com) to minimize rRNA contamination. One microgram of mRNA per sample was used as a template for first-strand cDNA synthesis using SMART technology (Clontech Laboratories Inc, http://www.clontech.com/) to favour full-length synthesis. Double-stranded cDNA was made by 13 cycles of long-distance PCR. Complementary DNA was purified with QIAquick columns (Qiagen, http://www.qiagen.com/) to eliminate oligo dT and enzymes. The cDNA quality was verified with an Agilent 2100 Bioanalyzer (Nimblegen, http://www.nimblegen.com/).

### Library Preparation for Pyro-sequencing

Three micrograms of each cDNA sample were nebulized to produce fragments of a mean size between 400 and 800 bp. Preparation of cDNA fragment libraries and emulsion PCR conditions were performed as described in the Roche GS FLX manual. Pyro-sequencing was performed on a Roche Genome Sequencer FLX instrument (454 Life Science Roche Diagnostics, http://www.454.com/) at Lifesequencing S.L. (Valencia, Spain).

### Trimming and Assembly of Pyro-sequenced Reads

The quality of the reads was assessed with PERL scripts developed at Lifesequencing for trimming and validation of high-quality sequences. Adaptor sequences used for library preparation were entered in an adaptor-trimming database to the PERL Program. New SFF output files were generated with the sfftools (454 Life Science/Roche), keeping the largest starting trimpoint and the smallest ending trimpoint. Trimmed reads were assembled with NEWBLER version 2.3 (454 Life Science/Roche) with default parameters. Following quality control, when performing the assembly, some reads were removed due to short quality for the reads to be used.

### Annotation

We selected a wide set of reference proteins from taxonomically related organisms. In addition, we included all proteins form *eudicotyledons* with annotations for the terms: carbohydrate metabolic process, secondary metabolic process, cell-wall, cell-wall organization, and phytohormones, in order to have a complete reference protein representation for these specific aspects probably related with abscission process. The total number of reference proteins was 125,428. The inclusion of proteins from taxonomically distant organisms with rich functional annotations such as *Vitis vinifera* or *Ricinus communis,* allowed us to annotate new proteins that could be lost if we include proteins only from close organisms. To obtain a high quality annotation we chose a very restrictive level of similarity between the isotig and the annotator reference protein. The similarity required must be high to sufficiently support the inference of function from the reference protein. In this work, BLAST E value lower than 10^−20^ was required for function inference. It is important to note that the smaller the E value is, the higher similarity between sequences is, and thus, the greater the confidence of the function assignment is. The massive BLASTX of all isotigs against the 125,428 reference proteins was performed using a cloud computing environment (Amazon web services).

### Quantification of the Expression Levels

The reference proteins were proteins representative of UniRef90 clusters. This strategy fixed a minimum similarity distance between reference proteins and was the basis of our clustering of isotigs for obtaining unigenes and quantifying their expression levels. The name of each unigene was inferred from the name of the UniRef90 representative proteins that annotated each unigene. We quantified the expression for these unigenes, here defined as clusters of isotigs annotated by the same reference protein. The number of reads assigned to each isotig was calculated taking into account that the reads of each contig were counted only one time. Given that isotigs represent transcribed isoforms, it could be possible that different isotigs sharing some contigs were clustered within the same unigene. In those cases, the reads of each contig was counted only one time. The normalization of the absolute values of the number of reads was done based on [60]. We obtained the RPKM (Reads Per Kilobase of exon model per Million mapped reads). In this case we used the length of the reference protein in nucleotides since we were working without a reference genome and then without exon models. This normalization allows the comparison of the expression values between unigenes from the same or from different samples [60].

### Differential Expression Analysis

The method used for the analysis of differential expression in this work was edgeR [61], a Bioconductor package for differential expression analysis of digital gene-expression data able to account for biological variability. EdgeR models count data using on overdispersed Poisson model, and use an empirical Bayes procedure to moderate the degree of over-dispersion across genes. For the analysis of the differential expression with Edge R the input was a table of counts, with rows corresponding to genes/proteins and columns to samples. EdgeR models the data as negative binomial (NB) distributed, Y_gi_∼NB(M_i_p_gj_, *Ф*_g_) for gene g and sample i. Here M_i_ is the library size (total number of reads), *Ф*_g_ is the dispersion, and p_gj_ is the relative abundance of gene g in experimental group j to which sample i belongs. The NB distribution reduces to Poisson when *Ф*_g_  = 0. This is an especially appropriate method to be used in RNA-Seq projects [62], [63].

In this work an isotig was considered differentially expressed during abscission when at stage transition it exhibited highly significant difference in read abundance at P<0.01.

### GO Annotations

GO annotations [64] were obtained from Uniprot and inferred from the GO annotations of the proteins representative of each unigene. GO Terms coming from the 3 different GO ontologies (Biological process, Molecular function and Cellular component) were analyzed separately. We found the number of proteins annotated with each term. In the GOSlim analysis, every GO term was translated into a GO Term taken from a set of selected general GO Terms in order to provide a more general and homogeneous perspective of the GO Terms found in a sample. To perform the GOSlim analysis, we selected the GOSlim terms proposed by the European Institute of Bioinformatics (EBI) as GO Terms selected for studies in Plants. The GO-slim studies were developed using Bio4j (http://www.bio4j.com/), a graph database that integrates all Uniprot, GO, taxonomy, RefSeq and Enzyme database elements in nodes connected by edges that represent their relationships. We selected a subset of terms to gain a broad functional overview and, using bio4j at the back-end, we obtained the GO-slim results. At this selected granularity level we obtained the functional profile of GO-slim terms that allowed us to highlight general features.

Hierarchical clustering analysis was used to group contigs according to their transcription profile using the tool developed by [65, http://rana.lbl.gov/eisen/].

### Phylogenetic Analysis

Phylogenetic trees were constructed based on similarity searches performed with BLASTp programs with default parameters in protein-sequence databases provided by the National Center for Biotechnology Information server (http://www.ncbi.nlm.nih.gov). Amino-acid sequences were aligned with ClustalW (version 2.0.3) [66].

### Sequence Deposition

The complete set of 454 sequences will be deposited at GenBank upon publication. The dataset can also be obtained from the authors via FTP upon request.

### Quantitative RT-PCR

Total RNA (2 µg) was reverse-transcribed with random hexamers and Superscript III (Invitrogen), according to the manufacturer’s instructions. Purified cDNA (2 ng) was used as a template for qRTPCR. qRT-PCR assays were performed with gene-specific primers. Genes and their primers are shown in Table S16. The cDNA was amplified using SYBRGreen-PCR Master kit (Applied Biosystems, Foster City, CA, USA) containing an AmpliTaq Gold polymerase on an iCycler (BioRad Munich, Germany), following the protocol provided by the supplier. Samples were subjected to thermal cycling conditions of DNA polymerase activation at 94°C, 45 s at 55°C, 45 s at 72°C, and 45 s at 80 °C; a final elongation step of 7 min at 72°C was performed. The melting curve was designed to increase 0.5°C every 10 s from 62°C. The amplicon was analysed by electrophoresis and sequenced once for identity confirmation. qRT-PCR efficiency was estimated via a calibration dilution curve and slope calculation. Expression levels were determined as the number of cycles needed for the amplification to reach a threshold fixed in the exponential phase of the PCR (CT). The data were normalized for the quantity of melon *ubiquitin* gene. Duplicates from three biological replicates were used in two independent experiments.

## Supporting Information

Figure S1 Summary of different parameters during the sequencing and assembly data of the study of the melon AZ transcriptome at 36, 38, and 40 DPA to allow insight into the transcriptional events that underlie fruit-AZ function during MFA. A, Read length distribution. A total of 483,704 good-quality sequence reads (134,158,280 bp) were obtained from the 3 samples (fruit-AZ at 36, 38 and 40 DPA). B, Contig length distribution. A total of 14,162 contigs were obtained from the Newbler assembly of the 483704 redundant reads. The average contig length is around 500 bases. C, Contig read total distribution from fruit-AZ 454 sequencing data. The majority of the contigs consisted of less than 10 reads. D, Isotigs length distribution. 12,871 isotigs were obtained after Newbler gene modeling.
(TIFF)

Figure S2 Nine clusters representing expression signatures in the three stages of melon AZ (36, 38, and 40 DPA). Clusters A1, A2, and A3 contained the 524, 182, and 89 most abundant transcripts in the pre-cell separation sample only, respectively. Cluster B1 includes the 1,219 most abundant transcripts in the partial cell separation sample (38 DPA, early induction of abscission) exclusively. The smaller cluster B2 included the most abundant transcripts in both the early and late induction of abscission samples (38 and 40 DPA). The cluster B3 contained 7 transcripts more abundant in both the pre-cell and partial cell separation samples, and the transcripts with lower expression levels in almost complete cell separation sample (40 DPA, late induction of abscission). Cluster C1 included the 407 most abundant transcripts in the almost complete cell-separation sample (40 DPA, late induction of abscission) exclusively. Cluster C2 and C3 contained the 93 and 37 most abundant transcripts in the almost complete cell separation sample, respectively, but in C2 the transcript levels also rose in the partial cell-separation sample relative to the pre-cell separation sample, whereas in C3 levels fell in the partial cell-separation sample relative to the pre-cell separation sample.
(TIFF)

Figure S3 Enriched gene ontology (GO) terms during early (A) and late (B) induction of melon MFA for UniProt IDs under biological processes. Enrichment analysis included: 1,790 transcripts with increased transcript accumulation (orange bars), and 899 transcripts with decreased transcript accumulation (green bars) during early induction of MFA; and 802 transcripts with increased transcript accumulation (orange bars), and 1,310 transcripts with decreased transcript accumulation (green bars) during late induction of MFA.
(TIFF)

Figure S4 Enriched gene ontology (GO) terms during early (A) and late (B) induction of melon MFA for UniProt IDs under metabolic functions. Enrichment analysis included: 1,790 transcripts with increased transcript accumulation (orange bars), and 899 transcripts with decreased transcript accumulation (green bars) during early induction of MFA; and 802 transcripts with increased transcript accumulation (orange bars), and 1,310 transcripts with decreased transcript accumulation (green bars) during late induction of MFA.
(TIFF)

Figure S5 Enriched gene ontology (GO) terms during early (A) and late (B) induction of mature-fruit abscission in melon for UniProt IDs under cellular compartments. Enrichment analysis included: 1,790 transcripts with increased transcript accumulation (orange bars), and 899 transcripts with decreased transcript accumulation (green bars) during early induction of MFA; and 802 transcripts with increased transcript accumulation (orange bars), and 1,310 transcripts with decreased transcript accumulation (green bars) during late induction of MFA.
(TIFF)

Figure S6 Expression profile of family genes encoding various transport proteins during melon MFA. Sequences were selected after establishing a P<0.01 (group I). Relative expression is as in Figure 3.
(TIFF)

Figure S7 Phylogenetic analysis of melon ACS with other ACS genes. The sequences included in this alignment are from melon (Cucurbit Genomics Database; http://www.icugi.org/cgi-bin/ICuGI/EST/home.cgi?organism=melon), and arabidopsis (http://www.arabidopsis.org/). The ACS proteins studied from our work are enclosed in an open box. The UniProt IDs followed by asterisks indicate transcripts showing significant variations during abscission (group I). Relative expression is as in Figure 3.
(TIF)

Figure S8 Phylogenetic analysis of melon ACO with other ACO genes. The sequences included in this alignment are from melon (Cucurbit Genomics Database; http://www.icugi.org/cgi-bin/ICuGI/EST/home.cgi?organism=melon), and arabidopsis (http://www.arabidopsis.org/). The ACO proteins studied from our work are enclosed in an open box. The UniProt IDs followed by asterisks indicate transcripts showing significant variations during abscission (group I). Relative expression is as in Figure 3.
(TIF)

Figure S9 Phylogenetic analysis of melon ERF with other AP2/ERF genes. The sequences included in this alignment are from our work. Relative expression is as in Figure 3.
(TIFF)

Figure S10 Metabolic map of phytohormone signal transduction pathways in melon-fruit AZ. Relative expression is as in Figure 3.
(TIF)

Figure S11 Phylogenetic analysis of melon MADS box with other MADS genes. The sequences included in this alignment are from our work, and arabidopsis (http://www.arabidopsis.org/). The UniProt IDs followed by asterisks indicate transcripts showing significant variations during abscission (group I). Relative expression is as in Figure 3.
(TIFF)

Table S1 Genes up-regulated during early induction of MFA in the first experiment.
(XLS)

Table S2 Genes down-regulated during early induction of MFA in the first experiment.
(XLS)

Table S3 Genes up-regulated during late induction of MFA in the second experiment.
(XLS)

Table S4 Genes down-regulated during late induction of MFA in the second experiment.
(XLS)

Table S5 Genes up-regulated during MFA in both the first and second experiments (early and late induction of melon MFA).
(XLS)

Table S6 Genes down-regulated during MFA in both the first and second experiments (early and late induction of melon MFA).
(XLS)

Table S7 Most abundant transcripts in fruit-AZ during melon MFA. Sequences were selected after establishing a P<0.01.The table shows the total read count in RPKMx1000 for each gene after normalization across the 3 samples: (a) AZ pre-cell separation (36 DPA), (b) AZ partial cell separation (38 DPA, early induction of abscission), (c) almost complete cell separation (40 DPA, late induction of abscission).
(DOC)

Table S8 Cell-wall-related genes induced or repressed in fruit-AZ at 38 DPA relative to 36 DPA during early induction of melon MFA. Sequences were selected after establishing a P<0.01.The table shows the total read count in RPKMx1000 for each gene after normalization across the 3 samples: (a) AZ pre-cell separation (36 DPA), (b) AZ partial-cell separation (38 DPA), (c) almost complete-cell separation (40 DPA).
(DOC)

Table S9 Cell-wall-related genes induced or repressed in fruit-AZ at 40 DPA relative to 38 DPA during late induction of melon MFA. Sequences were selected after establishing a P<0.01.The table shows the total read count in RPKMx1000 for each gene after normalization across the 3 samples: (a) AZ pre-cell separation (36 DPA), (b) AZ partial-cell separation (38 DPA), (c) almost complete-cell separation (40 DPA).
(DOC)

Table S10 Vesicle-trafficking-related genes repressed or induced in fruit-AZ during melon MFA. Sequences were selected after establishing a P<0.01. The table shows the total read count in RPKMx1000 for each gene after normalization across the 3 samples: (a) AZ pre-cell separation (36 DPA), (b) AZ partial cell separation (38 DPA, early induction of abscission), (c) almost complete cell separation (40 DPA, late induction of abscission). The vesicle-trafficking-related genes showed in Figure 4 are indicated in bold.
(DOC)

Table S11 Protein-phosphorylation-associated genes induced or repressed in fruit-AZ during melon MFA. Sequences were selected after establishing a P<0.01.The table shows the total read count in RPKMx1000 for each gene after normalization across the 3 samples: (a) AZ pre-cell separation (36 DPA), (b) AZ partial-cell separation (38 DPA), (c) almost complete-cell separation (40 DPA).
(DOC)

Table S12 Ethylene- and polyamine-related genes induced or repressed in fruit-AZ during melon MFA. Sequences were selected after establishing a P<0.01.The table shows the total read count in RPKMx1000 for each gene after normalization across the 3 samples: (a) AZ pre-cell separation (36 DPA), (b) AZ partial-cell separation (38 DPA), (c) almost complete-cell separation (40 DPA).
(DOC)

Table S13 Auxin- and ABA-related genes induced or repressed in fruit-AZ during melon MFA. Sequences were selected after establishing a P<0.01.The table shows the total read count in RPKMx1000 for each gene after normalization across the 3 samples: (a) AZ pre-cell separation (36 DPA), (b) AZ partial-cell separation (38 DPA), (c) almost complete-cell separation (40 DPA).
(DOC)

Table S14 GA-, BR-, CK-, JA- and SA-related genes induced or repressed in fruit-AZ during melon MFA. Sequences were selected after establishing a P<0.01.The table shows the total read count in RPKMx1000 for each gene after normalization across the 3 samples: (a) AZ pre-cell separation (36 DPA), (b) AZ partial-cell separation (38 DPA), (c) almost complete-cell separation (40 DPA).
(DOC)

Table S15 Transcription factor genes induced or repressed in fruit-AZ during melon MFA. Sequences were selected after establishing a P<0.01.The table shows the total read count in RPKMx1000 for each gene after normalization across the 3 samples: (a) AZ pre-cell separation (36 DPA), (b) AZ partial-cell separation (38 DPA), (c) almost complete-cell separation (40 DPA).
(DOC)

Table S16 PCR-primers used in this study.
(DOC)
