# Supplementary figures and images for: Transcriptomic Events Involved in Melon Mature-Fruit Abscission Comprise the Sequential Induction of Cell-Wall Degrading Genes Coupled to a Stimulation of Endo and Exocytosis
Source: PLoS One. 2013 Mar 6;8(3):e58363. doi: 10.1371/journal.pone.0058363 (PMC3590154; doi:10.1371/journal.pone.0058363)

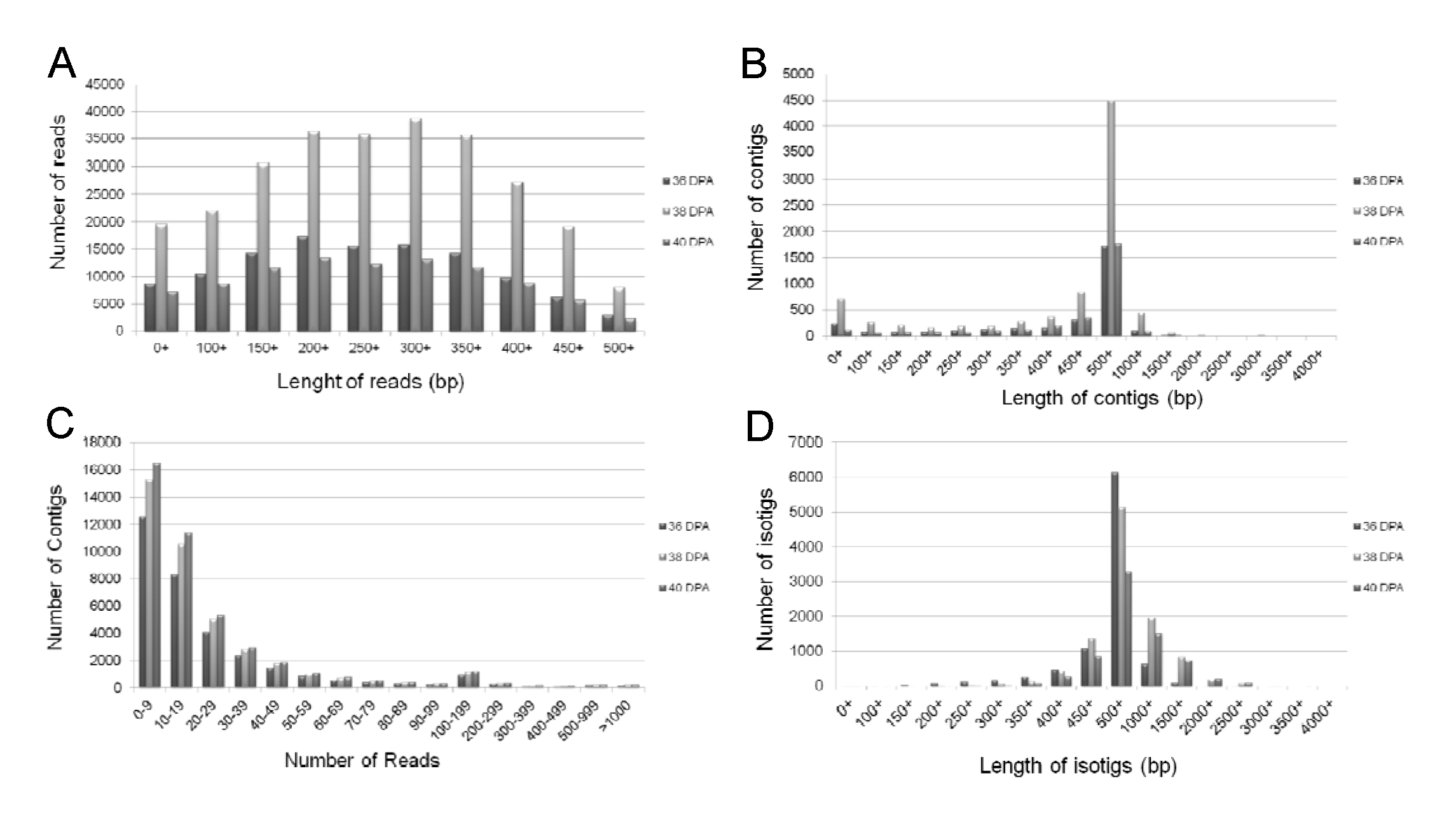

Supplement: Figure S1 — Summary of different parameters during the sequencing and assembly data of the study of the melon AZ transcriptome at 36, 38, and 40 DPA to allow insight into the transcriptional events that underlie fruit-AZ function during MFA. A, Read length distribution. A total of 483,704 good-quality sequence reads (134,158,280 bp) were obtained from the 3 samples (fruit-AZ at 36, 38 and 40 DPA). B, Contig length distribution. A total of 14,162 contigs were obtained from the Newbler assembly of the 483704 redundant reads. The average contig length is around 500 bases. C, Contig read total distribution from fruit-AZ 454 sequencing data. The majority of the contigs consisted of less than 10 reads. D, Isotigs length distribution. 12,871 isotigs were obtained after Newbler gene modeling. (TIFF) [file pone.0058363.s001.tiff]

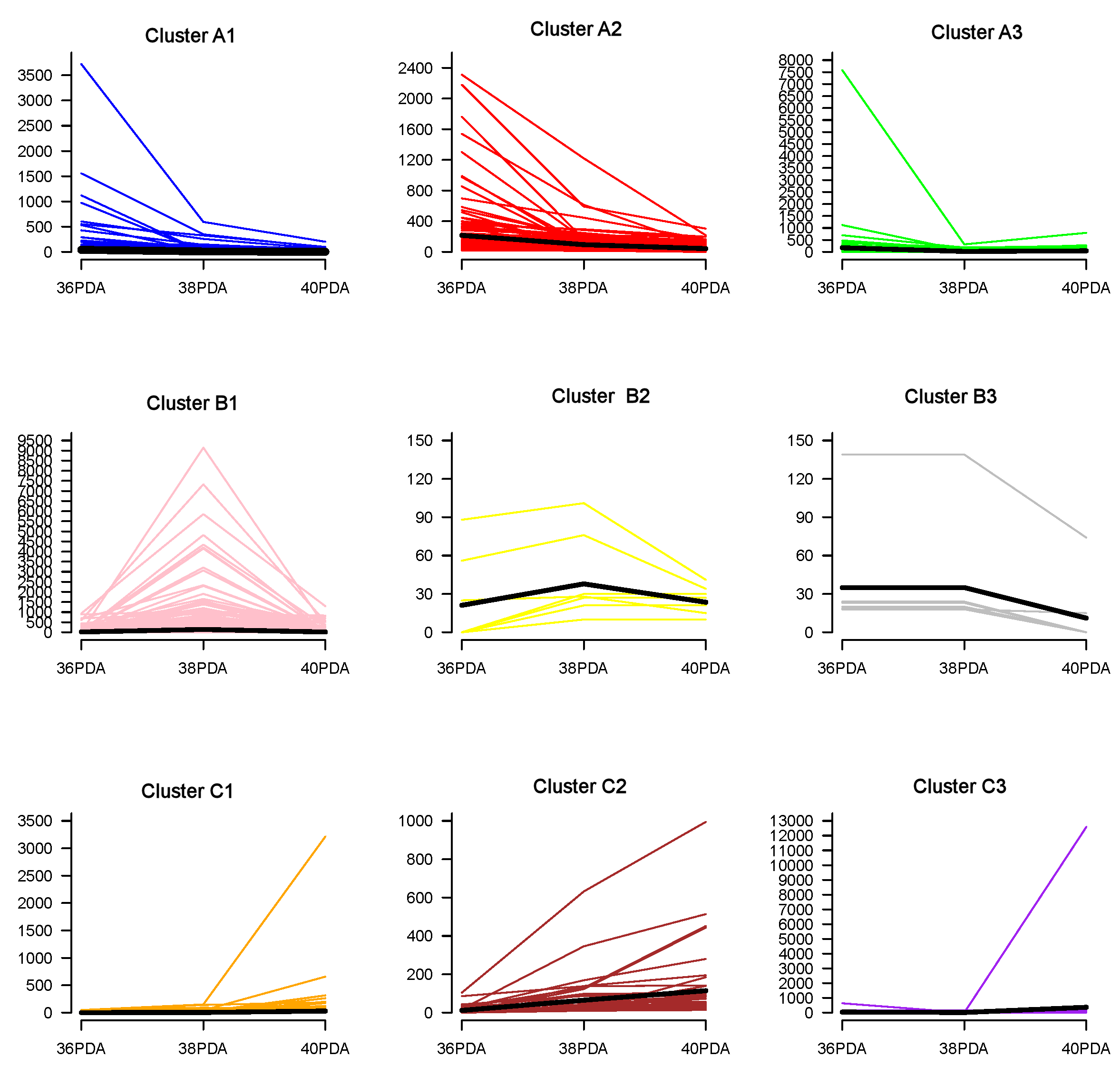

Supplement: Figure S2 — Nine clusters representing expression signatures in the three stages of melon AZ (36, 38, and 40 DPA). Clusters A1, A2, and A3 contained the 524, 182, and 89 most abundant transcripts in the pre-cell separation sample only, respectively. Cluster B1 includes the 1,219 most abundant transcripts in the partial cell separation sample (38 DPA, early induction of abscission) exclusively. The smaller cluster B2 included the most abundant transcripts in both the early and late induction of abscission samples (38 and 40 DPA). The cluster B3 contained 7 transcripts more abundant in both the pre-cell and partial cell separation samples, and the transcripts with lower expression levels in almost complete cell separation sample (40 DPA, late induction of abscission). Cluster C1 included the 407 most abundant transcripts in the almost complete cell-separation sample (40 DPA, late induction of abscission) exclusively. Cluster C2 and C3 contained the 93 and 37 most abundant transcripts in the almost complete cell separation sample, respectively, but in C2 the transcript levels also rose in the partial cell-separation sample relative to the pre-cell separation sample, whereas in C3 levels fell in the partial cell-separation sample relative to the pre-cell separation sample. (TIFF) [file pone.0058363.s002.tiff]

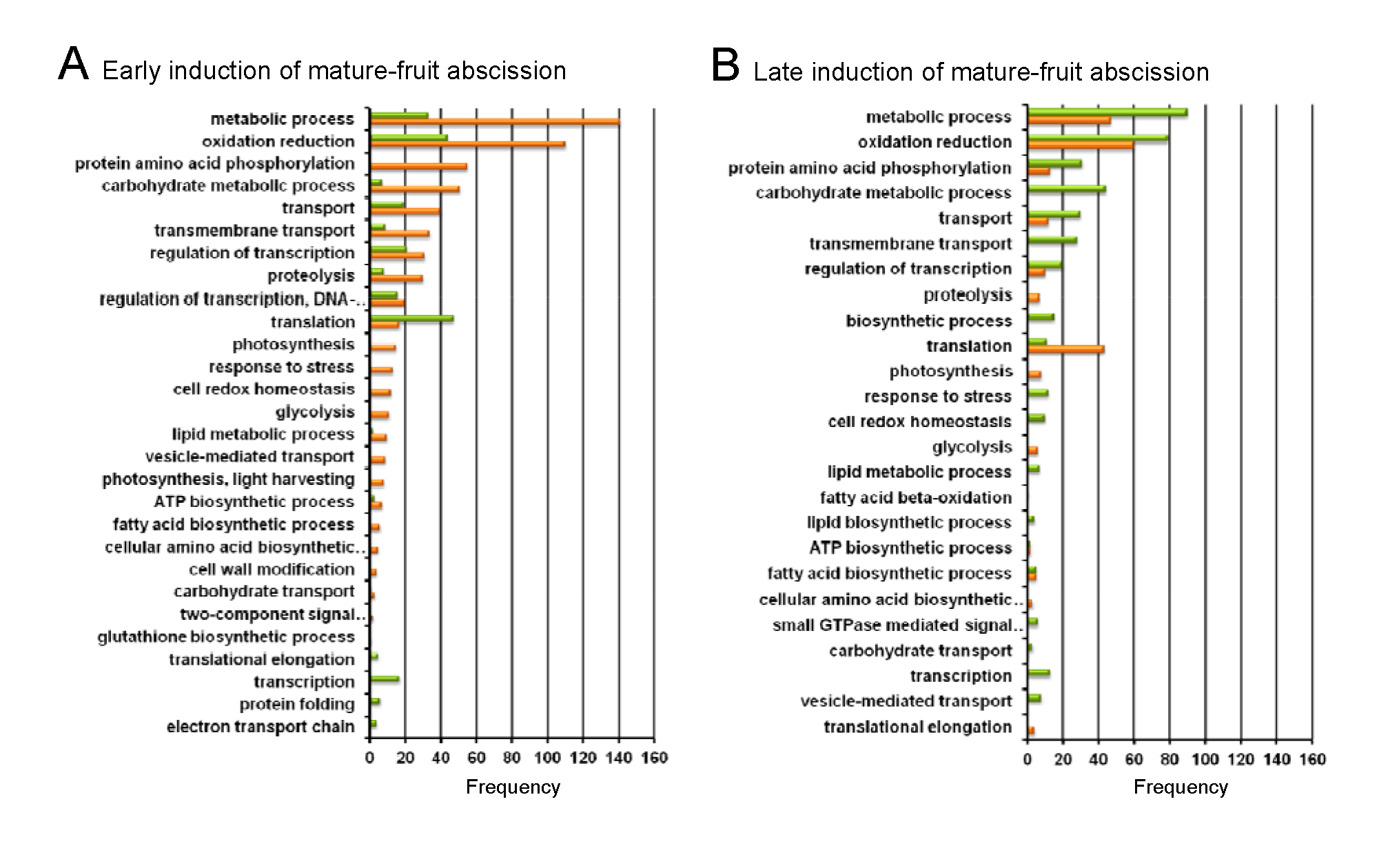

Supplement: Figure S3 — Enriched gene ontology (GO) terms during early (A) and late (B) induction of melon MFA for UniProt IDs under biological processes. Enrichment analysis included: 1,790 transcripts with increased transcript accumulation (orange bars), and 899 transcripts with decreased transcript accumulation (green bars) during early induction of MFA; and 802 transcripts with increased transcript accumulation (orange bars), and 1,310 transcripts with decreased transcript accumulation (green bars) during late induction of MFA. (TIFF) [file pone.0058363.s003.tiff]

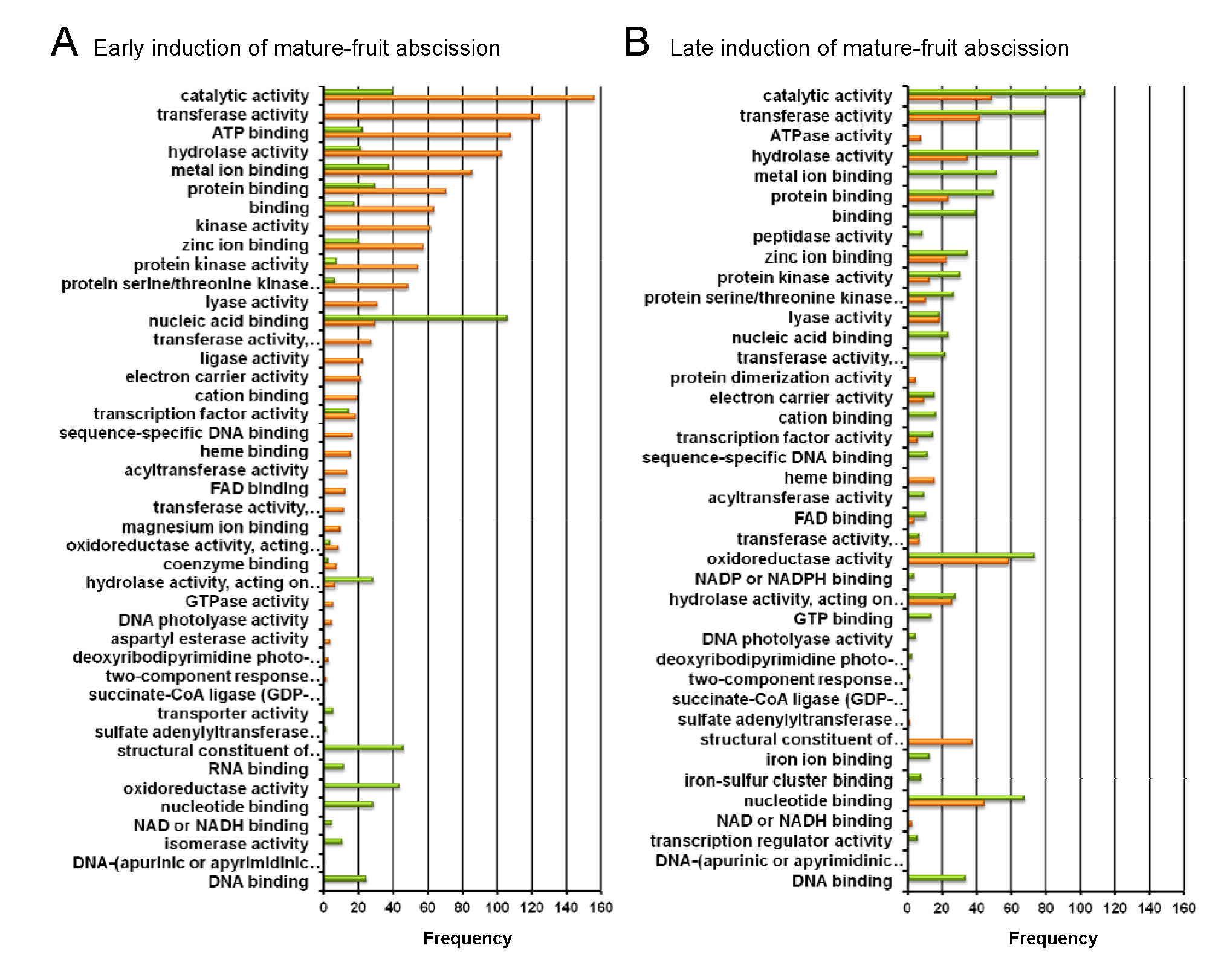

Supplement: Figure S4 — Enriched gene ontology (GO) terms during early (A) and late (B) induction of melon MFA for UniProt IDs under metabolic functions. Enrichment analysis included: 1,790 transcripts with increased transcript accumulation (orange bars), and 899 transcripts with decreased transcript accumulation (green bars) during early induction of MFA; and 802 transcripts with increased transcript accumulation (orange bars), and 1,310 transcripts with decreased transcript accumulation (green bars) during late induction of MFA. (TIFF) [file pone.0058363.s004.tiff]

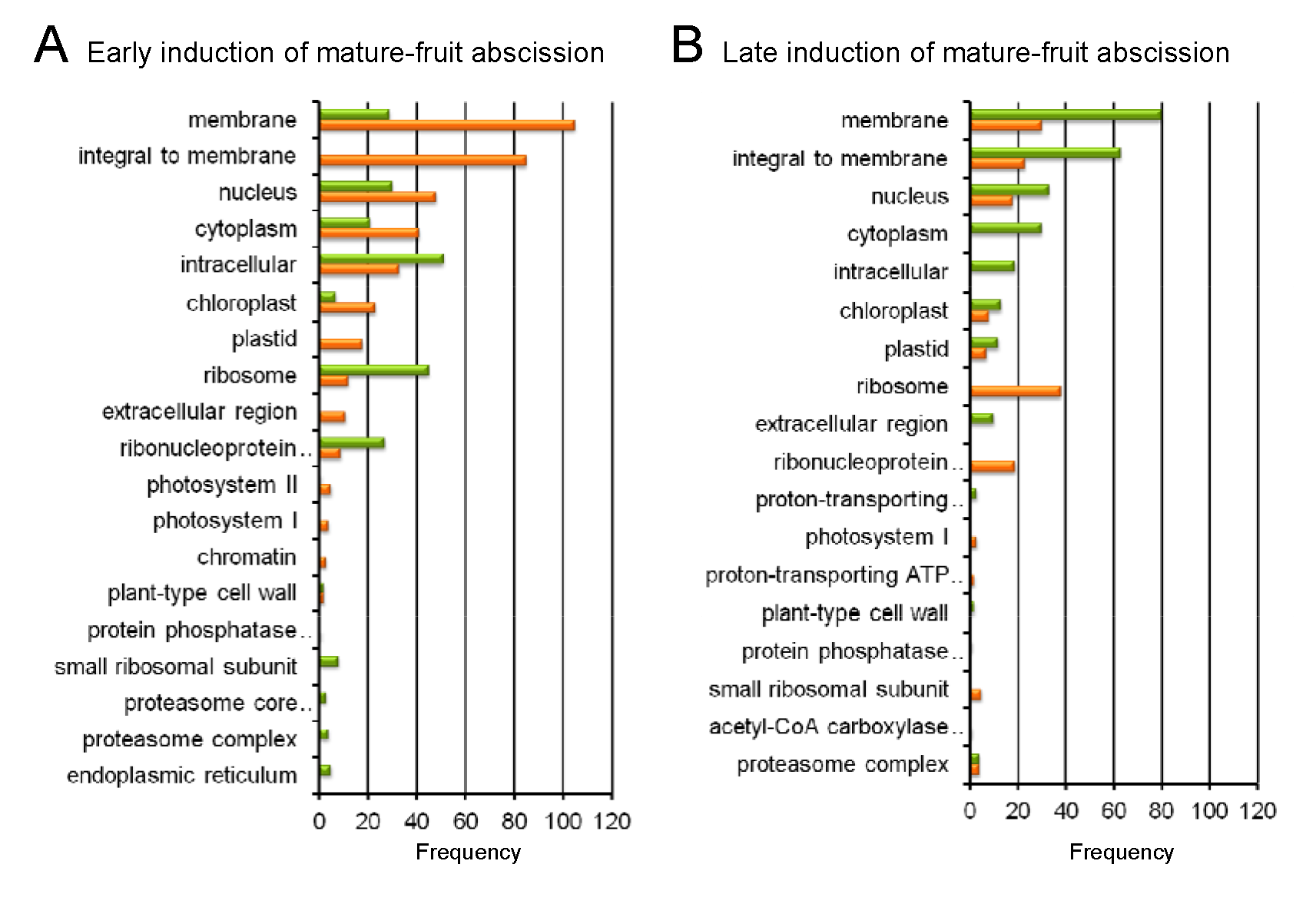

Supplement: Figure S5 — Enriched gene ontology (GO) terms during early (A) and late (B) induction of mature-fruit abscission in melon for UniProt IDs under cellular compartments. Enrichment analysis included: 1,790 transcripts with increased transcript accumulation (orange bars), and 899 transcripts with decreased transcript accumulation (green bars) during early induction of MFA; and 802 transcripts with increased transcript accumulation (orange bars), and 1,310 transcripts with decreased transcript accumulation (green bars) during late induction of MFA. (TIFF) [file pone.0058363.s005.tiff]

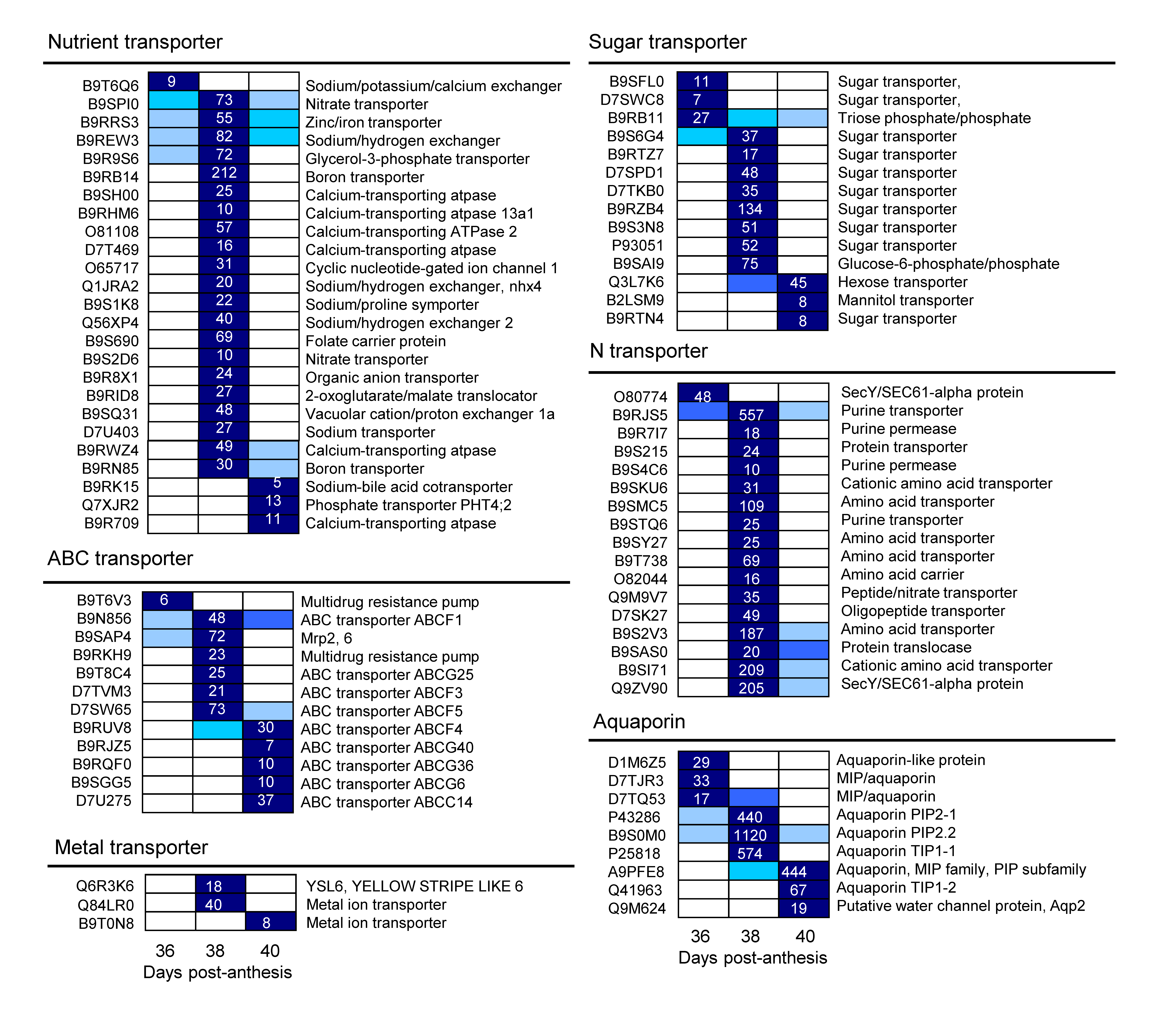

Supplement: Figure S6 — Expression profile of family genes encoding various transport proteins during melon MFA. Sequences were selected after establishing a P<0.01 (group I). Relative expression is as in Figure 3. (TIFF) [file pone.0058363.s006.tiff]

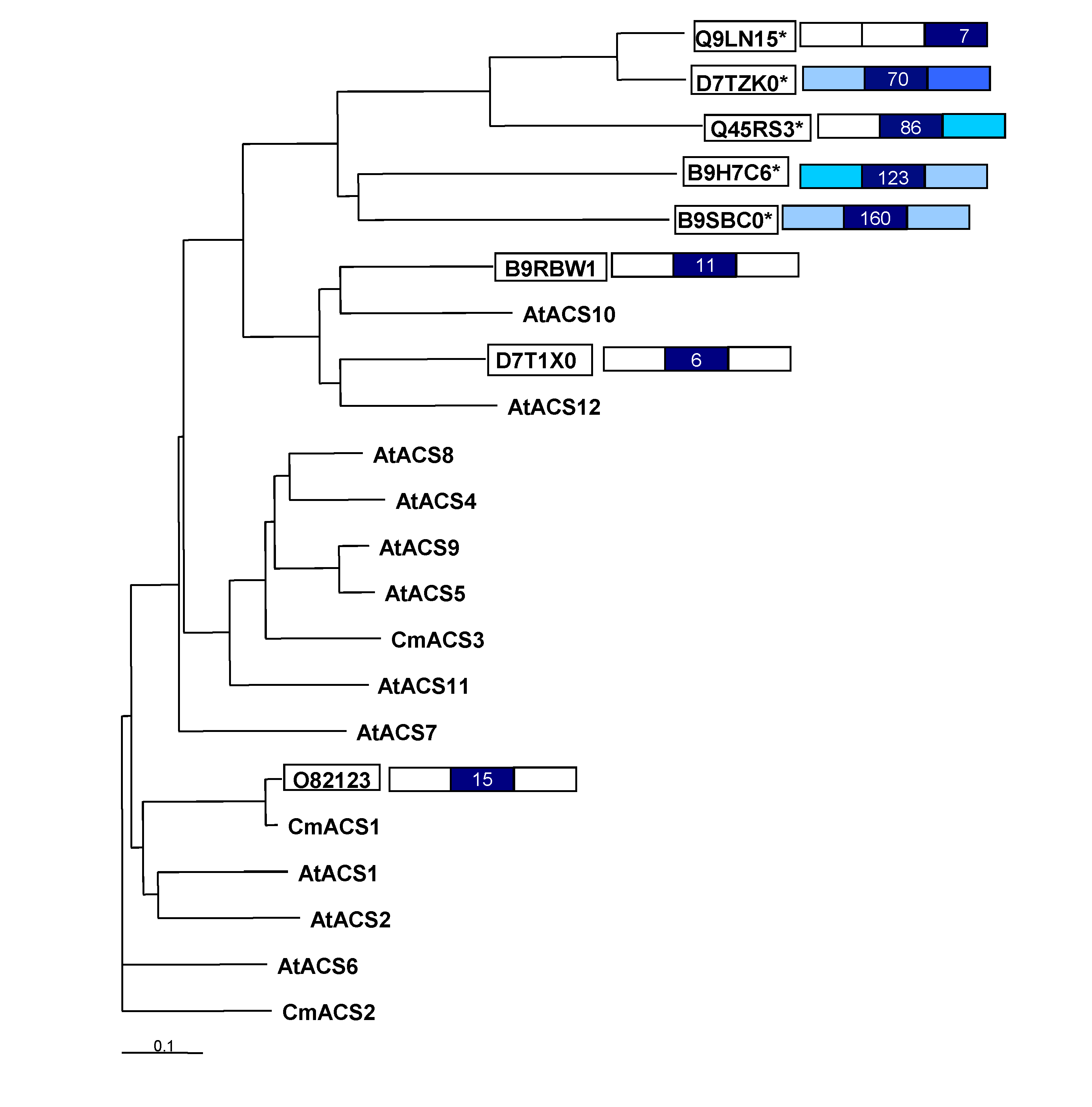

Supplement: Figure S7 — Phylogenetic analysis of melon ACS with other ACS genes. The sequences included in this alignment are from melon (Cucurbit Genomics Database; http://www.icugi.org/cgi-bin/ICuGI/EST/home.cgi?organism=melon), and arabidopsis (http://www.arabidopsis.org/). The ACS proteins studied from our work are enclosed in an open box. The UniProt IDs followed by asterisks indicate transcripts showing significant variations during abscission (group I). Relative expression is as in Figure 3. (TIF) [file pone.0058363.s007.tif]

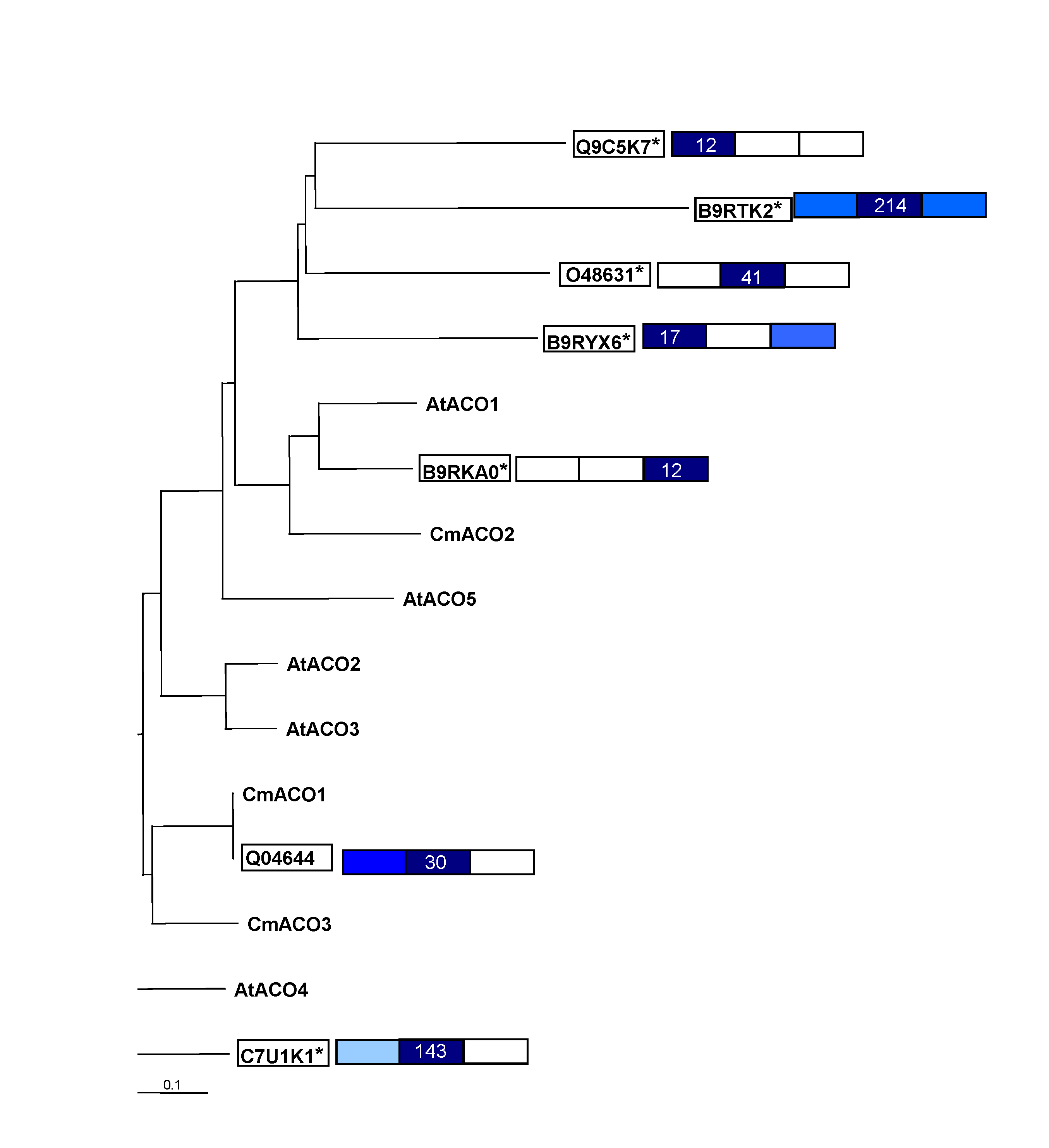

Supplement: Figure S8 — Phylogenetic analysis of melon ACO with other ACO genes. The sequences included in this alignment are from melon (Cucurbit Genomics Database; http://www.icugi.org/cgi-bin/ICuGI/EST/home.cgi?organism=melon), and arabidopsis (http://www.arabidopsis.org/). The ACO proteins studied from our work are enclosed in an open box. The UniProt IDs followed by asterisks indicate transcripts showing significant variations during abscission (group I). Relative expression is as in Figure 3. (TIF) [file pone.0058363.s008.tif]

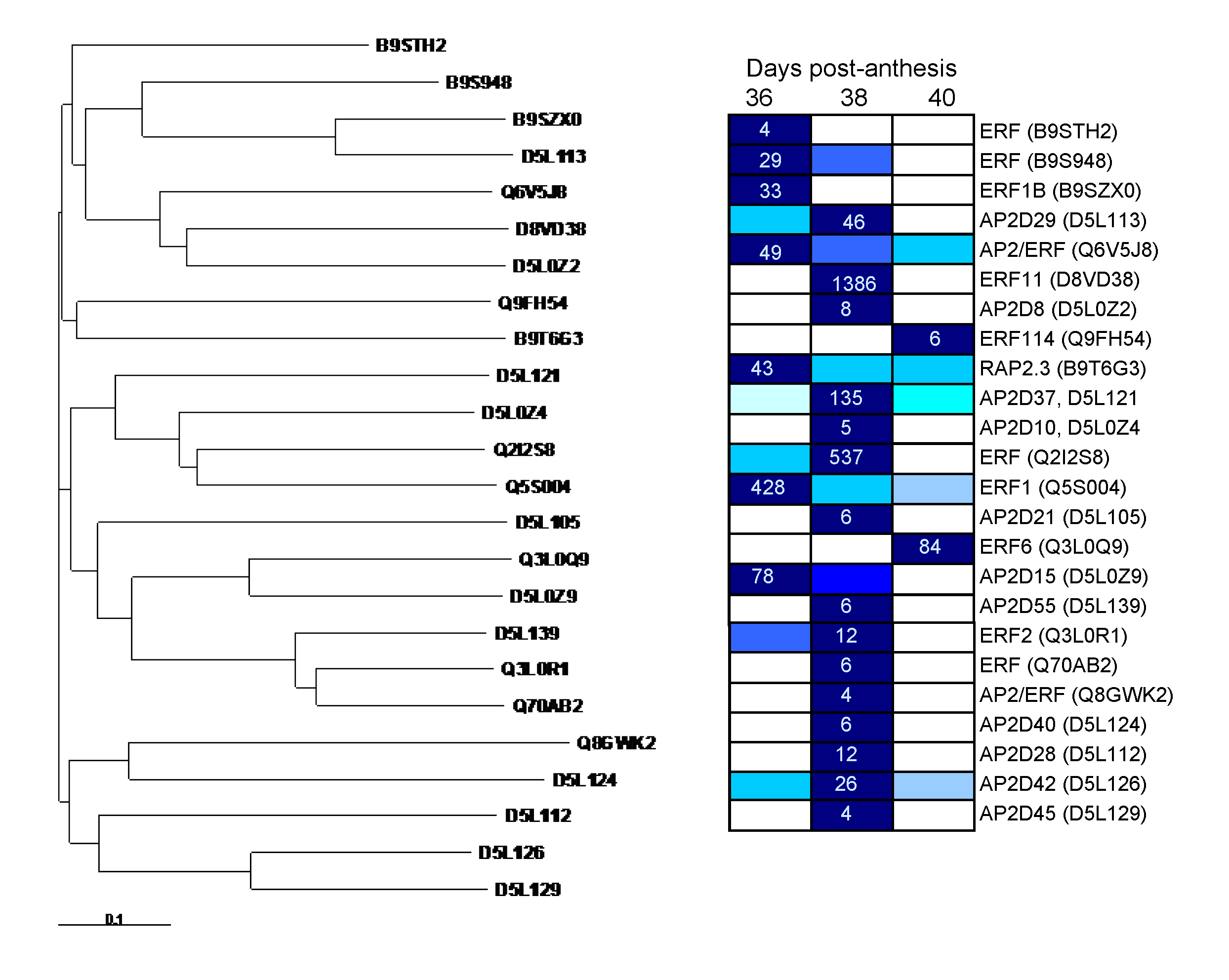

Supplement: Figure S9 — Phylogenetic analysis of melon ERF with other AP2/ERF genes. The sequences included in this alignment are from our work. Relative expression is as in Figure 3. (TIFF) [file pone.0058363.s009.tiff]

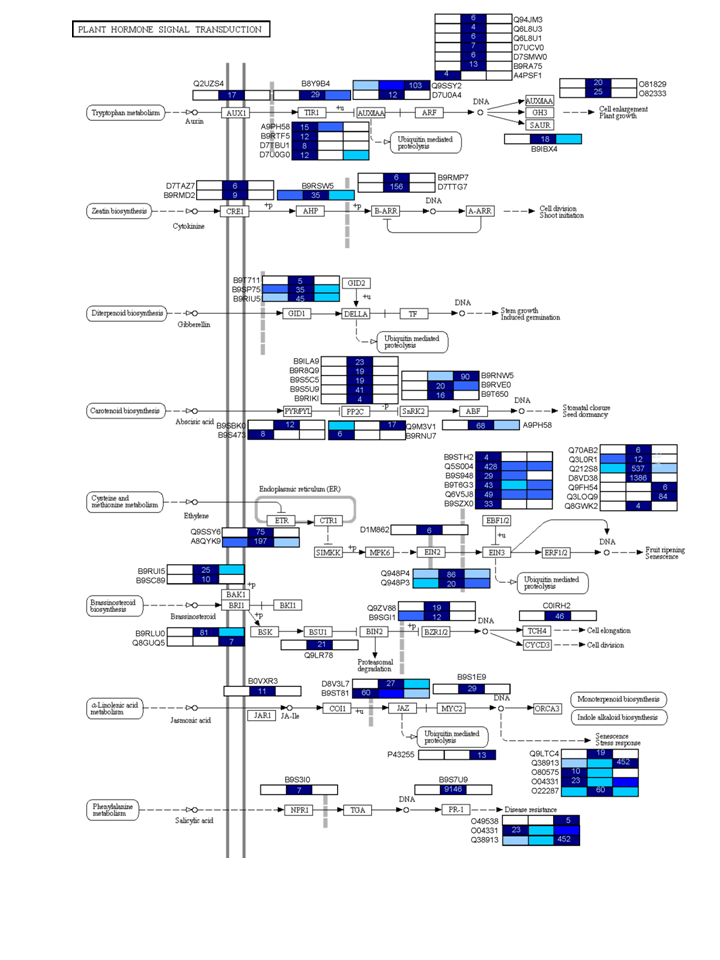

Supplement: Figure S10 — Metabolic map of phytohormone signal transduction pathways in melon-fruit AZ. Relative expression is as in Figure 3. (TIF) [file pone.0058363.s010.tif]

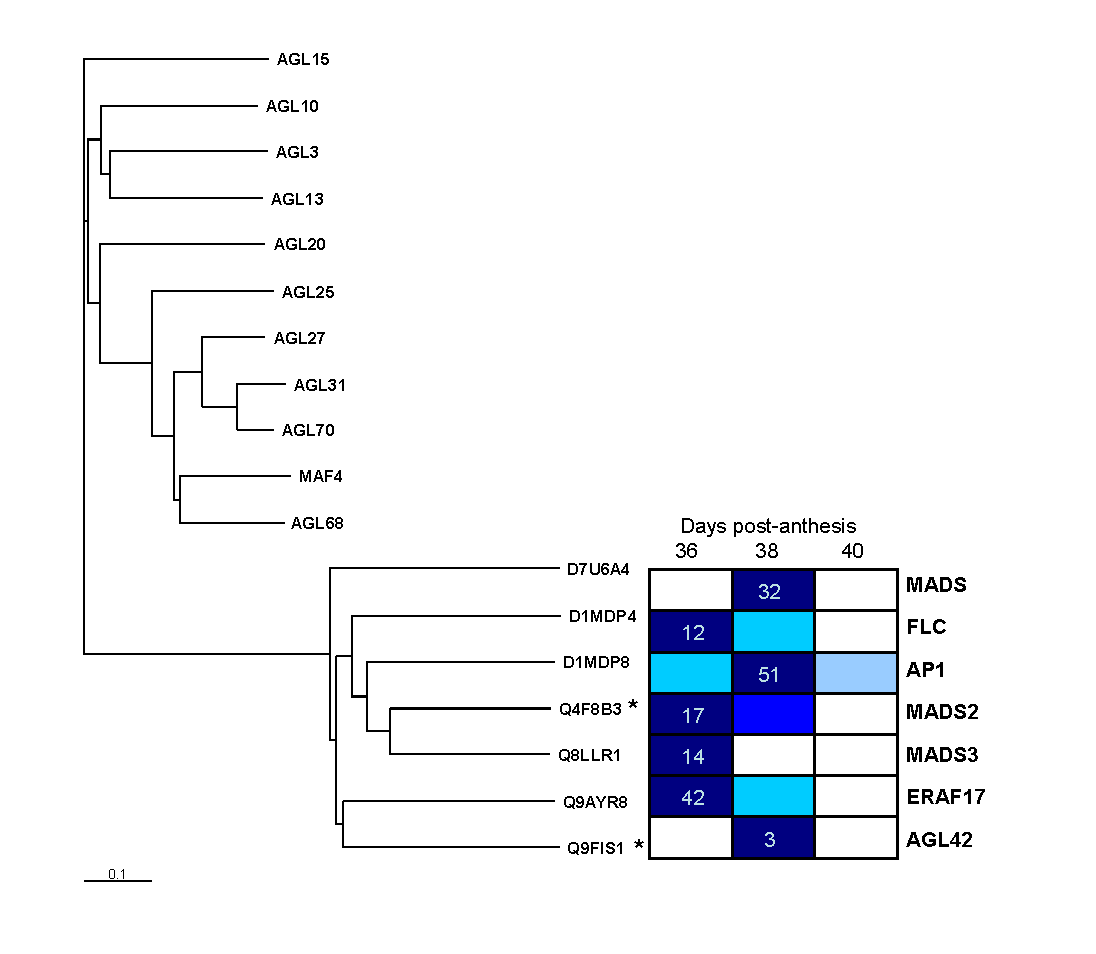

Supplement: Figure S11 — Phylogenetic analysis of melon MADS box with other MADS genes. The sequences included in this alignment are from our work, and arabidopsis (http://www.arabidopsis.org/). The UniProt IDs followed by asterisks indicate transcripts showing significant variations during abscission (group I). Relative expression is as in Figure 3. (TIFF) [file pone.0058363.s011.tiff]
