# Supplementary material for: Transcriptomic Events Involved in Melon Mature-Fruit Abscission Comprise the Sequential Induction of Cell-Wall Degrading Genes Coupled to a Stimulation of Endo and Exocytosis
Source: PLoS One. 2013 Mar 6;8(3):e58363. doi: 10.1371/journal.pone.0058363 (PMC3590154; doi:10.1371/journal.pone.0058363)
Supplement: Table S7 — Most abundant transcripts in fruit-AZ during melon MFA. Sequences were selected after establishing a P<0.01.The table shows the total read count in RPKMx1000 for each gene after normalization across the 3 samples: (a) AZ pre-cell separation (36 DPA), (b) AZ partial cell separation (38 DPA, early induction of abscission), (c) almost complete cell separation (40 DPA, late induction of abscission). (DOC) [file pone.0058363.s018.doc]

**Table S7** Most abundant transcripts in fruit-AZ during melon MFA. Sequences were selected after establishing a P<0.01. The table shows the total read count in RPKMx1000 for each gene after normalization across the 3 samples: (a) AZ pre-cell separation (36 DPA), (b) AZ partial cell separation (38 DPA, early induction of abscission), (c) almost complete cell separation (40 DPA, late induction of abscission).

| **Cluster** | **UniProt ID** | **36 DPA** | **38 DPA** | **40 DPA** | **Description** |
| --- | --- | --- | --- | --- | --- |
| A1 | B9ILN4 | 1229.79 | 0 | 0 | Uncharacterized protein, POPTRDRAFT_669317 = *Populus trichocarpa* |
|  | Q850K6 | 3140.05 | 0 | 0 | Non-specific lipid-transfer protein = *Vitis vinifera* |
|  | B9ILN4 | 1229.79 | 0 | 0 | Uncharacterized protein, POPTRDRAFT_669317 = *Populus trichocarpa* |
|  | D7U9D2 | 552.44 | 0 | 0 | Uncharacterized protein, VIT_00026088001 = *Vitis vinifera* |
|  | B9S715 | 312.03 | 0 | 0 | ATP-dependent peptidase, RCOM_1331450 = *Ricinus communis* |
|  | D7SZQ9 | 215.05 | 0 | 0 | Uncharacterized protein, VIT_00027946001 = *Vitis vinifera* |
|  | D7SIJ6 | 251.24 | 0 | 0 | Serine/threonine-protein phosphatase, VIT_00008096001= *Vitis vinifera* |
|  | P12333 | 137.32 | 0 | 0 | Chlorophyll a-b binding protein. chloroplastic (LHCII type I CAB) (LHCP) = *Spinacia oleracea* |
|  | A5YWM1 | 168.20 | 0 | 0 | Glutathione S-transferase (Glutathione transferase Tau1), GSTU1 = *Citrus sinensis* |
|  | A8D009 | 119.59 | 0 | 0 | Ferritin, Fer3 = *Pyrus pyrifolia* |
|  | A5AWP0 | 242.18 | 0 | 0 | Uncharacterized protein, VITISV_004511 = *Vitis vinifera* |
|  | B9RMM2 | 147.94 | 0 | 0 | Uncharacterized protein, RCOM_1081740 = *Ricinus communis* |
|  | Q56Z40 | 82.03 | 0 | 0 | RSH3, At1g54130 |
|  | D7SLU5 | 99.23 | 0 | 0 | Uncharacterized protein, VIT_00018215001 = *Vitis vinifera* |
|  | B9R7F5 | 112.57 | 0 | 0 | Uncharacterized protein, RCOM_1591260 = *Ricinus communis* |
|  | A5AYZ1 | 38.55 | 0 | 0 | Malic enzyme, VITISV_024155 = *Vitis vinifera* |
|  | P57106 | 74.29 | 0 | 0 | Malate dehydrogenase. cytoplasmic 2, At5g43330 MWF20.2 |
|  | P26569 | 89.13 | 0 | 0 | Histone H1.2, At2g30620 T06B20.3 |
|  | B9S552 | 117.44 | 0 | 0 | Uncharacterized protein, RCOM_1721330 = *Ricinus communis* |
|  | B9SWT3 | 54.18 | 0 | 0 | 3-isopropylmalate dehydrogenase = *Ricinus communis* |
| A2 | A9PAH1 | 2972.60 | 385.84 | 59.36 | Uncharacterized protein, POPTRDRAFT _830079 = *Populus trichocarpa* |
|  | B9R7X8 | 6497.89 | 2590.71 | 232.06 | Low-molecular-weight cysteine-rich protein LCR78. RCOM_1595110 = *Ricinus communis* |
|  | D7SQM1 | 3337.12 | 316.28 | 268.93 | Uncharacterized protein, VIT_00000754001 = *Vitis vinifera* |
|  | D7TD89 | 3705.62 | 216.45 | 142.85 | Uncharacterized protein, VIT_00030113001 = *Vitis vinifera* |
|  | D7TQG9 | 3844.79 | 1044.09 | 534.39 | Uncharacterized protein, VIT_00025626001 = *Vitis vinifera* |
|  | D7UB79 | 4033.14 | 2129.14 | 376.96 | Uncharacterized protein, VIT_00019304001 = *Vitis vinifera* |
|  | Q8LAD0 | 1275.81 | 137.25 | 67.97 | Pyridoxal biosynthesis protein PDX2 (Probable glutamine amidotransferase) (AtPDX2) (Protein EMBRYO DEFECTIVE 2407). PDX2 EMB2407 At5g60540 muf9.190 |
|  | D7T0X0 | 1426.99 | 90.90 | 63.36 | Uncharacterized protein, VIT_00036785001 = *Vitis vinifera* |
|  | A5C9I9 | 2504.27 | 777.77 | 653.84 | Uncharacterized protein, VITISV_021844 VIT_00009367001 = *Vitis vinifera* |
|  | Q8LEH3 | 488.44 | 112.21 | 0 | Cinnamoyl-CoA reductase-like protein = *Arabidopsis thaliana* |
|  | B9SZD6 | 284.18 | 50.56 | 4.27 | Anthocyanin 5-aromatic acyltransferase = *Ricinus communis* |
|  | P59232 | 1154.98 | 414.01 | 312.10 | Ubiquitin-40S ribosomal protein S27a-2, RPS27AB UBQ6 At2g47110 F14M4.6 |
|  | A5B3D4 | 361.65 | 18.51 | 0 | Uncharacterized protein, VITISV_028922 = *Vitis vinifera* |
|  | B9R8J3 | 1011.59 | 646.37 | 236.23 | Phosphoprotein ECPP44 = *Ricinus communis* |
|  | D7TK38 | 215.77 | 102.56 | 63.86 | Uncharacterized protein, VIT_00021436001 = *Vitis vinifera* |
|  | D7SMR3 | 865.24 | 340.42 | 271.86 | Uncharacterized protein, VIT_00036450001= *Vitis vinifera* |
|  | B9S940 | 815.04 | 500.00 | 235.77 | 28 kDa heat-and acid-stable phosphoprotein = *Ricinus communis* |
|  | Q93VF1 | 751.72 | 427.58 | 234.48 | At2g04520/T1O3.7 |
|  | A9PBT2 | 555.55 | 176.15 | 116.53 | Uncharacterized protein, POPTRDRAFT_548036 = *Populus trichocarpa* |
|  | A9PDV6 | 741.72 | 476.82 | 331.12 | Uncharacterized protein, POPTRDRAFT_666912 = *Populus trichocarpa* |
| A3 | D7UBL2 | 3090.90 | 126.72 | 253.44 | Uncharacterized protein, VIT_00010910001 = *Vitis vinifera* |
|  | P49690 | 18061.90 | 752.38 | 1897.61 | 60S ribosomal protein L23 (Protein EMBRYO DEFECTIVE 2171), RPL23A At1g04480 F19P19.5 |
|  | A5B6Y9 | 462.15 | 119.52 | 169.32 | Photosystem I P700 chlorophyll a apoprotein = *Vitis vinifera* |
|  | D7U796 | 248.21 | 0 | 10.43 | Uncharacterized protein, VIT_00013934001 = *Vitis vinifera* |
|  | D7UCZ6 | 792.07 | 178.21 | 458.74 | Uncharacterized protein, VIT_00012395001 = *Vitis vinifera* |
|  | D7T502 | 566.10 | 0 | 139.66 | Uncharacterized protein, VIT_00012876001 = *Vitis vinifera* |
|  | D7SN28 | 864.36 | 455.17 | 482.75 | Uncharacterized protein, VIT_00031366001 = *Vitis vinifera* |
|  | Q6L3T8 | 759.25 | 301.58 | 306.87 | 60S ribosomal protein L34, putative = *Solanum demissum* |
|  | Q08682 | 185.68 | 0 | 128.63 | 40S ribosomal protein Sa-1 (Laminin receptor homolog) (p40), RPSaA At1g72370 T10D10.16 |
|  | D7T9H6 | 246.28 | 91.29 | 94.47 | Uncharacterized protein, VIT_00012054001 = *Vitis vinifera* |
|  | B9SCC9 | 247.03 | 0 | 81.21 | Steroid binding protein = *Ricinus communis* |
|  | A5AKT0 | 117.41 | 19.70 | 111.89 | Uncharacterized protein, VITISV_021574 = *Vitis vinifera* |
|  | P59259 | 631.07 | 200.64 | 511.32 | Histone H4. At1g07660 F24B9.25 |
|  | Q8VZB9 | 194.44 | 0 | 114.19 | 60S ribosomal protein L10a-1, RPL10AA At1g08360 T27G7.6 |
|  | B9RKC5 | 393.51 | 134.25 | 145.83 | 60S ribosomal protein L28 = *Ricinus communis* |
|  | Q8GY66 | 614.45 | 184.73 | 377.51 | Uncharacterized protein, At4g10262 |
|  | O23940 | 210.40 | 0 | 28.36 | Acyl carrier protein = *Fragaria vesca* |
|  | B6VC54 | 312.78 | 123.28 | 143.83 | 60S ribosomal protein L27A = *Vernicia fordii* |
|  | B9RDU1 | 71.36 | 0 | 15.02 | Cysteine synthase = *Ricinus communis* |
|  | D7U1A2 | 124.29 | 0 | 52.73 | 40S ribosomal protein S12 = *Vitis vinifera* |
| B1 | A5BKB8 | 74.92 | 4173.87 | 278.57 | Uncharacterized protein, VITISV_001840 = *Vitis vinifera* |
|  | A5C4I3 | 802.91 | 9694.44 | 678.57 | Uncharacterized protein, VITISV_012740 = *Vitis vinifera* |
|  | B9RMD4 | 0 | 2175.88 | 0 | Uncharacterized protein, RCOM_1079860 = *Ricinus communis* |
|  | B9S7U9 | 37.26 | 18935.81 | 548.65 | STS14 protein = *Ricinus communis* |
|  | D7SHE0 | 0 | 2655.91 | 0 | Uncharacterized protein, VIT_00007582001 = *Vitis vinifera* |
|  | E0CU96 | 3.02 | 2083.44 | 2.59 | Uncharacterized protein, VIT_00023471001 = *Vitis vinifera* |
|  | O80432 | 0 | 6584.12 | 0 | Mitochondrial small heat shock protein = *Solanum lycopersicum* |
|  | O81245 | 0 | 3309.69 | 0 | Polygalacturonase,MPG2 = *Cucumis melo* |
|  | B2ZP02 | 30.91 | 2218.35 | 114.97 | Beta-1.3-glucanase = *Vitis vinifera* |
|  | D7TIT4 | 0 | 1210.85 | 0 | Uncharacterized protein, VIT_00033671001 = *Vitis vinifera* |
|  | B9SIQ2 | 0 | 2618.55 | 470.79 | Alpha-amylase/subtilisin inhibitor = *Ricinus communis* |
|  | Q0WLP3 | 0 | 2976.04 | 0 | Uncharacterized protein = *Arabidopsis thaliana* |
|  | B9T5B7 | 0 | 917.44 | 0 | Glycosyltransferase = *Ricinus communis* |
|  | Q40345 | 31.56 | 1466.51 | 15.39 | Isocitrate dehydrogenase [NADP]. chloroplastic (IDH) (EC 1.1.1.42) (IDP) (NADP(+)-specific ICDH) (Oxalosuccinate decarboxylase) = *Medicago sativa* |
|  | D8VD38 | 0 | 1680.00 | 0 | Ethylene response factor 11, ERF11 = *Actinidia deliciosa* |
|  | P22503 | 0 | 806.45 | 0 | Endoglucanase (Abscission cellulase) (Endo-1.4-beta-glucanase) = *Phaseolus vulgaris* |
|  | P52408 | 0 | 1133.33 | 0 | Glucan endo-1.3-beta-glucosidase. basic isoform ((1->3)-beta-glucan endohydrolase) ((1->3)-beta-glucanase) (Beta-1.3-endoglucanase) (PpGns1) = *Prunus persica* |
|  | B9S561 | 0 | 827.66 | 0 | Pectate lyase = Ricinus communis |
|  | Q8H159 | 680.52 | 4270.60 | 942.37 | Polyubiquitin 10 [Cleaved into: Ubiquitin], UBQ10 At4g05320 C17L7.240 |
|  | B9S0K2 | 0 | 485.52 | 111.11 | Phenylalanine ammonia-lyase = *Ricinus communis* |
| B2 | D7U403 | 0 | 12.67 | 12.67 | Uncharacterized protein, VIT_00022810001 = *Vitis vinifera* |
|  | B9T5V3 | 0 | 19.60 | 19.60 | Auxin-induced protein 5NG4 = *Ricinus communis* |
| B3 | B9RQ45 | 313.06 | 313.06 | 166.66 | Uncharacterized protein, RCOM_0954650 = *Ricinus communis* |
|  | A5AIY9 | 46.51 | 46.51 | 0 | Uncharacterized protein, VITISV_013093 = *Vitis vinifera* |
|  | B9SX19 | 38.38 | 38.38 | 0 | Transporter, RCOM_1259420 = *Ricinus communis* |
|  | D7SH16 | 47.28 | 47.28 | 0 | Uncharacterized protein, VIT_00008576001 = *Vitis vinifera* |
|  | E0CQ54 | 33.68 | 33.68 | 0 | Uncharacterized protein, VIT_00009900001 = *Vitis vinifera* |
|  | B9SXB7 | 20.90 | 20.90 | 17.42 | Prolyl 4-hydroxylase alpha subunit, RCOM_0782750 = *Ricinus communis* |
|  | D7U851 | 16.52 | 16.52 | 0 | Uncharacterized protein, VIT_00027465001 = *Vitis vinifera* |
| C1 | A5AWY8 | 0 | 0 | 79.01 | Uncharacterized protein, VITISV_008680 = *Vitis vinifera* |
|  | A5C6A5 | 0 | 0 | 70.55 | Uncharacterized protein, VITISV_042891 = *Vitis vinifera* |
|  | B9SEN5 | 0 | 0 | 814.81 | Early nodulin = *Ricinus communis* |
|  | A2ICD0 | 0 | 0 | 94.36 | Phenylalanine ammonia-lyase = *Vitis vinifera* |
|  | D7TIQ9 | 0 | 0 | 92.94 | Uncharacterized protein, VIT_00033642001 = *Vitis vinifera* |
|  | B9STU5 | 0 | 0 | 57.26 | Phenylalanine ammonia-lyase = *Ricinus communis* |
|  | D7TBY0 | 0 | 0 | 364.48 | Uncharacterized protein, VIT_00015498001 = *Vitis vinifera* |
|  | Q2V4A1 | 0 | 0 | 222.22 | Uncharacterized protein, At2g05440 |
|  | Q3L0Q9 | 0 | 0 | 106.87 | Ethylene-responsive element binding protein ERF6 = *Gossypium mexicanum* |
|  | B9S693 | 0 | 0 | 78.54 | Peroxidase 72 = *Ricinus communis* |
|  | Q93VI9 | 0 | 0 | 107.80 | Uncharacterized protein M4I22.200, M4I22.200 At4g27390 |
|  | Q41963 | 0 | 0 | 88.27 | Aquaporin TIP1-2 (Gamma-tonoplast intrinsic protein 2) (Gamma-TIP2) (Tonoplast intrinsic protein 1-2) (AtTIP1;2). TIP1-2 SITIP TIP2 At3g26520 MFE16.17 |
|  | D7T4J5 | 0 | 0 | 77.98 | Ferritin (Fragment) = *Vitis vinifera* |
|  | Q93ZH5 | 0 | 0 | 54.82 | At1g21080/T22I11_9 |
|  | D7U4W1 | 0 | 0 | 63.06 | Uncharacterized protein, VIT_00023919001 = *Vitis vinifera* |
|  | Q8GVF5 | 28.57 | 28.57 | 80.27 | D07. full insert sequence) (cDNA clone:006-308-B01. full insert sequence) OJ1340_C08.131 Os07g0639800 OsJ_25302 = *Oryza sativa* |
|  | B9S1P5 | 0 | 0 | 70.01 | Uncharacterized protein, RCOM_0867050 = *Ricinus communis* |
|  | C8CMH6 | 0 | 0 | 97.04 | Non-symbiotic haemoglobin = *Malus hupehensis* |
|  | A5C200 | 0 | 0 | 76.14 | Uncharacterized protein, VITISV_009604 = *Vitis vinifera* |
|  | B9DHJ1 | 0 | 0 | 38.56 | AT1G11910 protein |
|  | D7TAM5 | 129.03 | 129.03 | 243.72 | Uncharacterized protein, VIT_00010327001 = *Vitis vinifera* |
|  | A5BDB9 | 0 | 0 | 25.59 | Uncharacterized protein, VITISV_027249 = *Vitis vinifera* |
| C2 | B9SZV0 | 75.11 | 234.74 | 5032.86 | Histone h1/h5 = *Ricinus communis* |
|  | D7STB3 | 84.50 | 206.57 | 3089.20 | Uncharacterized protein, VIT_00029731001 = *Vitis vinifera* |
|  | O65773 | 106.66 | 648.20 | 1020.51 | Peroxidase, aprx = *Cucurbita pepo* |
|  | A9PFE8 | 0 | 142.85 | 515.67 | Aquaporin. MIP family, PIP subfamily (Putative uncharacterized protein) = *Populus trichocarpa* |
|  | B9R9G7 | 12.08 | 16.11 | 318.22 | Arginine/serine-rich splicing factor = *Ricinus communis* |
|  | B9SWM8 | 0 | 6.95 | 128.74 | UDP-glucuronosyltransferase = *Ricinus communis* |
|  | Q9LKX1 | 14.12 | 108.54 | 179.83 | Cinnamate 4-hydroxylase CYP73, C4H2 = *Citrus sinensis* |
|  | P25819 | 0 | 18.29 | 94.17 | Catalase-2, CAT2 CAT At4g35090 M4E13.140 |
|  | A7Y7M7 | 0 | 33.57 | 244.60 | Putative wound-induced protein = *Prunus dulcis* |
|  | C4P7Y8 | 0 | 217.52 | 305.16 | Calcineurin B-like protein 01 = *Vitis vinifera* |
|  | B9SCY3 | 25.64 | 102.56 | 284.38 | Uncharacterized protein, RCOM_1282260 = *Ricinus communis* |
|  | D7UAU0 | 63.57 | 242.26 | 324.74 | Uncharacterized protein, VIT_00014842001 = *Vitis vinifera* |
|  | B9RNW5 | 0 | 15.11 | 68.02 | CBL-interacting serine/threonine-protein kinase = *Ricinus communis* |
|  | B9T0K9 | 13.73 | 43.04 | 107.14 | Plasminogen activator inhibitor 1 RNA-binding protein = *Ricinus communis* |
|  | B9SFD0 | 0 | 3.86 | 31.50 | ATATH13 = *Ricinus communis* |
|  | D7TPA7 | 21.89 | 26.76 | 133.81 | Uncharacterized protein, VIT_00011077001 = *Vitis vinifera* |
|  | A5BTC0 | 100.87 | 140.35 | 333.33 | Uncharacterized protein, VITISV_039385 = *Vitis vinifera* |
|  | B9VQ34 | 15.93 | 31.86 | 84.55 | Class IV chitinase = *Pyrus pyrifolia* |
|  | B9RUV8 | 4.12 | 13.75 | 32.55 | ATP-dependent transporter = *Ricinus communis* |
|  | B9T4J2 | 0 | 35.02 | 82.48 | Casein kinase II. alpha chain = *Ricinus communis* |
| C3 | Q9AVA8 | 638.81 | 0 | 13496.24 | Endochitinase MCHT-2 = *Cucumis melo* |
|  | B9SWY1 | 64.79 | 17.02 | 144.97 | Pyruvate decarboxylase = *Ricinus communis* |
|  | D7TL61 | 13.45 | 0 | 78.72 | Uncharacterized protein, VIT_00029971001 = *Vitis vinifera* |
|  | D7TV58 | 36.50 | 33.33 | 238.09 | Uncharacterized protein, VIT_00019409001 = *Vitis vinifera* |
|  | B9MZ79 | 69.29 | 0 | 100.87 | Uncharacterized protein, POPTRDRAFT_827290 = *Populus trichocarpa* |
|  | D7TT82 | 14.15 | 9.43 | 57.38 | Uncharacterized protein, VIT_00000132001 = *Vitis vinifera* |
|  | B9R7W4 | 65.21 | 43.47 | 17.14 | Uncharacterized protein, RCOM_1594870= *Ricinus communis* |
|  | A5BVF4 | 11.75 | 0 | 66.23 | Uncharacterized protein, VITISV_027630 = *Vitis vinifera* |
|  | B9SI15 | 19.20 | 10.97 | 79.56 | Ubiquitin-protein ligase = *Ricinus communis* |
|  | A5AX15 | 156.66 | 136.66 | 290.00 | Ribosomal protein L36 = *Vitis vinifera* |
|  | Q03666 | 78.43 | 42.23 | 95.02 | Glutathione S-transferase (Auxin-induced protein PCNT107) = *Nicotiana tabacum* |
|  | B9SLQ3 | 96.38 | 38.15 | 108.43 | Glycine-rich RNA-binding protein = *Ricinus communis* |
|  | D7SJ03 | 27.43 | 0 | 39.63 | Uncharacterized protein, VIT_00008303001 = *Vitis vinifera* |
|  | C0LQ98 | 25.34 | 0 | 29.18 | Monodehydroascorbate reductase = *Malus domestica* |
|  | A9PA00 | 53.25 | 48.32 | 67.06 | Uncharacterized protein, POPTRDRAFT_728998 = *Populus trichocarpa* |
|  | Q1I1D9 | 22.63 | 20.93 | 32.25 | Pyruvate decarboxylase = *Citrus sinensis* |
|  | D7SIY8 | 24.96 | 14.68 | 52.86 | Uncharacterized protein, VIT_00008284001 = *Vitis vinifera* |
|  | A9P745 | 7.49 | 0 | 19.76 | Enolase, ENO1 = *Helianthus annuus* |
|  | B9RJ15 | 20.00 | 0 | 30.66 | J domain-containing protein spf31 = *Ricinus communis* |
|  | D7TRX1 | 46.46 | 38.38 | 68.68 | Uncharacterized protein, VIT_00007329001 = *Vitis vinifera* |
