# Supplementary material for: Transcriptomic Events Involved in Melon Mature-Fruit Abscission Comprise the Sequential Induction of Cell-Wall Degrading Genes Coupled to a Stimulation of Endo and Exocytosis
Source: PLoS One. 2013 Mar 6;8(3):e58363. doi: 10.1371/journal.pone.0058363 (PMC3590154; doi:10.1371/journal.pone.0058363)
Supplement: Table S9 — Cell-wall-related genes induced or repressed in fruit-AZ at 40 DPA relative to 38 DPA during late induction of melon MFA. Sequences were selected after establishing a P<0.01.The table shows the total read count in RPKMx1000 for each gene after normalization across the 3 samples: (a) AZ pre-cell separation (36 DPA), (b) AZ partial-cell separation (38 DPA), (c) almost complete-cell separation (40 DPA). (DOC) [file pone.0058363.s020.doc]

**Table S9** Cell-wall-related genes induced or repressed in fruit-AZ at 40 DPA relative to 38 DPA during late induction of melon MFA. Sequences were selected after establishing a P<0.01.The table shows the total read count in RPKMx1000 for each gene after normalization across the 3 samples: (a) AZ pre-cell separation (36 DPA), (b) AZ partial-cell separation (38 DPA), (c) almost complete-cell separation (40 DPA).

| **UniProt ID** | **36 DPA** | **38 DPA** | **40 DPA** | **Description** |
| --- | --- | --- | --- | --- |
| ***Induced transcripts*** |  |  |  |  |
| D7U7V9 | 0 | 0 | 45.04 | Pectinesterase = *Vitis vinifera* |
| B9RD90 | 0 | 60.31 | 60.95 | Pectinesterase = *Ricinus communis* |
| Q38913 | 25.02 | 115.28 | 403.93 | Extensin-1 (AtExt1) (AtExt4). EXT1 EXT4 At1g76930 F22K20.3 |
| Q09082 | 0 | 4.29 | 23.19 | Extensin (Class I), tegI = *Solanum lycopersicum* |
| B9RPJ5 | 0 | 0 | 7.26 | Chitinase = *Ricinus communis* |
| B9VQ34 | 15.93 | 31.86 | 84.55 | Class IV chitinase = *Pyrus pyrifolia* |
| Q41360 | 42.85 | 823.80 | 1223.80 | Pathogenesis-related protein PR-4 type (Fragment) = *Sambucus* |
| B9T775 | 0 | 0 | 12.87 | Beta-expansin 1a putative, Expansin-like CBD domain (1), Expansin-like EG45 domain (1) = *Ricinus communis* |
| D7U4W1 | 0 | 0 | 63.06 | Expansin-like CBD domain (1), Expansin-like EG45 domain (1) = *Vitis vinifera* |
| Q0KIX2 | 10.18 | 10.71 | 11.25 | Endoglucanase, CmEGase1 = *Cucumis melo* |
| Q9AVA8 | 683.81 | 0 | 13496.24 | Endochitinase, MCHT-2 = *Cucumis melo* |
| Q9FLC0 | 0 | 34.97 | 58.64 | Peroxidase 52 (Atperox P52) (EC 1.11.1.7) (ATP49). PER52 P52 At5g05340 K18I23.14 |
| ***Repressed transcripts*** |  |  |  |  |
| O81245 | 0 | 3309.69 | 0 | Polygalacturonase, MPG2 = *Cucumis melo* |
| B9RVQ4 | 0 | 48.57 | 0 | Polygalacturonase = *Ricinus communis* |
| B9SSX1 | 0 | 280.74 | 0 | Polygalacturonase = *Ricinus communis* |
| B9H3W4 | 0 | 79.11 | 0 | Pectinesterase = *Populus trichocarpa* |
| Q43111 | 0 | 14.91 | 0 | Pectinesterase 3 (PE 3) (Pectin methylesterase 3), MPE3 = *Phaseolus vulgaris* |
| B9R9Q8 | 0 | 875.40 | 0 | 21 kDa protein, enzyme inhibitor activity; pectinesterase activity = Ricinus communis |
| B9GXZ7 | 0 | 11.33 | 0 | Pectinesterase = *Populus trichocarpa* |
| B9S5X9 | 0 | 20.75 | 0 | Beta-fructofuranosidase. cell wall isozyme = *Ricinus communis* |
| Q01943 | 0 | 117.86 | 81.03 | Extensin (Class I) = *Solanum lycopersicum* |
| Q8W5B0 | 0 | 25.13 | 0 | Alpha-expansin 3 = *Cucumis sativus* |
| A1X8W4 | 0 | 599.03 | 0 | Beta expansin 1, EXPB1 = *Solanum tuberosum* |
| B9RW20 | 0 | 38.61 | 0 | Beta-expansin 3, putative = *Ricinus communis* |
| P54904 | 0 | 27.77 | 0 | Pyrroline-5-carboxylate reductase (P5C reductase) (P5CR) (Protein EMBRYO DEFECTIVE 2772). PROC1 EMB2772 At5g14800 T9L3_100 |
| Q9LVJ1 | 0 | 7.72 | 0 | Cucumisin-like serine protease; subtilisin-like protease, At3g14067 |
| P22503 | 0 | 806.45 | 0 | Endoglucanase (Abscission cellulase) (Endo-1.4-beta-glucanase) *= Phaseolus vulgaris* |
| B2ZP02 | 30.91 | 2218.35 | 114.97 | Beta-1.3-glucanase = *Vitis vinifera* |
| Q6V596 | 0 | 458.75 | 0 | Endo-1.4-beta-glucanase = *Malus domestica* |
| Q25B23 | 0 | 29.56 | 0 | Endo-beta-1.4-glucanase, eg4 = *Prunus persica* |
| D0R6J2 | 11.69 | 49.70 | 0 | 1.3-beta-glucan synthase (EC 2.4.1.34), cals4 = *Malus domestica* |
| P41376 | 0 | 43.68 | 28.31 | Eukaryotic initiation factor 4A-1 (eIF-4A-1) (ATP-dependent RNA helicase eIF4A-1) (DEAD-box ATP-dependent RNA helicase 4), TIF4A-1 RH4 At3g13920 |
| C0IRH2 | 0 | 46.60 | 0 | Xyloglucan endotransglucosylase/hydrolase 13 = *Actinidia deliciosa* |
| Q2HV87 | 0 | 31.70 | 0 | Alpha-1.4-glucan-protein synthase (UDP-forming) = *Medicago truncatula* |
| P52408 | 0 | 1133.33 | 0 | Glucan endo-1.3-beta-glucosidase. basic isoform (1->3)-beta-glucan endohydrolase) ((1->3)-beta-glucanase) (Beta-1.3-endoglucanase) (PpGns1) = *Prunus persica* |
| Q7XAS3 | 0 | 13.26 | 0 | Beta-D-glucosidase = *Gossypium hirsutum* |
| B9T066 | 0 | 11.82 | 0 | Alpha-glucosidase = *Ricinus communis* |
| B9T947 | 0 | 35.46 | 0 | Endo-1.4-beta-glucanase = *Ricinus communis* |
| Q9ZSP9 | 0 | 21.33 | 0 | Endo-beta-1.4-D-glucanase, Cel8 = *Solanum lycopersicum* |
| B9S561 | 0 | 827.66 | 0 | Pectate lyase = *Ricinus communis* |
| Q6U7H9 | 0 | 92.50 | 0 | Pectate lyase = *Malus domestica* |
| B9HDL7 | 0 | 26.65 | 0 | Beta-galactosidase = *Populus trichocarpa* |
| Q5CCP8 | 0 | 16.00 | 0 | Beta-galactosidase, PpGAL5 = *Pyrus pyrifolia* |
| B9HFB3 | 0 | 15.34 | 0 | Beta-galactosidase = *Populus trichocarpa* |
| B9SWC7 | 0 | 14.64 | 0 | Beta-galactosidase = *Ricinus communis* |
| C6F122 | 0 | 10.63 | 0 | Beta-galactosidase = *Glycine max* |
| D7TB77 | 0 | 24.40 | 2.00 | Beta-galactosidase = *Vitis vinifera* |
| B9RK64 | 0 | 7.14 | 0 | Beta-galactosidase = *Ricinus communis* |
| Q9LLT0 | 0 | 13.69 | 0 | Beta-galactosidase, TBG5 = *Solanum lycopersicum* |
| A2Q575 | 25.07 | 91.95 | 0 | Glycoside hydrolase. family 19 (uncharacterized protein) = *Medicago truncatula* |
| D7T7B8 | 0 | 182.07 | 0 | Whole genome shotgun sequence of line PN40024. scaffold_20.assembly12x (Fragment) = *Vitis vinifera* |
| Q9FE06 | 0 | 374.86 | 0 | AT5g64260/MSJ1_10 (Phi-1-like protein) (Putative phi-1 protein), At5g64260 |
| B9STK4 | 10.52 | 200.00 | 0 | Transferase = *Ricinus communis* |
| B9HIG2 | 58.17 | 73.86 | 0 | Predicted protein = *Populus trichocarpa* |
| D7T7P7 | 0 | 6.28 | 0 | Whole genome shotgun sequence of line PN40024. scaffold_39.assembly12x (Fragment) = *Vitis vinifera* |
| D7TVI6 | 3.59 | 8.09 | 0 | Whole genome shotgun sequence of line PN40024. scaffold_25.assembly12x (Fragment) = *Vitis vinifera* |
| Q8VYW6 | 0 | 74.59 | 0 | AT4g39640/T19P19_30 (At4g39640/T19P19_30) |
| D7TEC1 | 0 | 12.80 | 0 | Whole genome shotgun sequence of line PN40024. scaffold_59.assembly12x (Fragment) = *Vitis vinifera* |
| Q7M1K0 | 0 | 275.74 | 0 | Chitinase = *Lagenaria siceraria* |
