# Supplementary material for: Transcriptomic Events Involved in Melon Mature-Fruit Abscission Comprise the Sequential Induction of Cell-Wall Degrading Genes Coupled to a Stimulation of Endo and Exocytosis
Source: PLoS One. 2013 Mar 6;8(3):e58363. doi: 10.1371/journal.pone.0058363 (PMC3590154; doi:10.1371/journal.pone.0058363)
Supplement: Table S10 — Vesicle-trafficking-related genes repressed or induced in fruit-AZ during melon MFA. Sequences were selected after establishing a P<0.01. The table shows the total read count in RPKMx1000 for each gene after normalization across the 3 samples: (a) AZ pre-cell separation (36 DPA), (b) AZ partial cell separation (38 DPA, early induction of abscission), (c) almost complete cell separation (40 DPA, late induction of abscission). The vesicle-trafficking-related genes showed in Figure 4 are indicated in bold. (DOC) [file pone.0058363.s021.doc]

**Table S10.** Vesicle-trafficking-related genes repressed or induced in fruit-AZ during melon MFA. Sequences were selected after establishing a P<0.01. The table shows the total read count in RPKMx1000 for each gene after normalization across the 3 samples: (a) AZ pre-cell separation (36 DPA), (b) AZ partial cell separation (38 DPA, early induction of abscission), (c) almost complete cell separation (40 DPA, late induction of abscission). The vesicle-trafficking-related genes showed in Figure 3 are indicated in bold.

|  | **Uniprot ID** | **36 DPA** | **38 DPA** | **40 DPA** | **Putative ortholog** | **Gene description** |
| --- | --- | --- | --- | --- | --- | --- |
| Tubulin family |  |  |  |  |  |  |
|  | Q41973 | 43.85 | 0 | 0 | [At5g19780](http://www.arabidopsis.org/servlets/TairObject?type=locus&name=AT5G19780) | TUA5, tubulin alpha-5 |
|  | B9DHQ0 | 23.47 | 106.80 | 53.99 | [At5g19780](http://www.arabidopsis.org/servlets/TairObject?type=locus&name=AT5G19780) | TUA5, tubulin alpha-5 |
|  | B9S382 | 0 | 6.11 | 0 | At5g23860 | TUB8, tubulin beta 8 |
| Kinesin-like protein family |  |  |  |  |  |  |
|  | D7T6C8 | 0 | 13.90 | 0 | [At3g16630](http://www.arabidopsis.org/servlets/TairObject?type=locus&name=AT3G16630) | Kinesin-13A |
|  | B9RB32 | 0 | 7.82 | 0 | [AT1G27500](http://www.arabidopsis.org/servlets/TairObject?type=locus&name=AT1G27500) | Kinesin light chain |
|  | A5BHF6 | 0 | 5.39 | 0 | [AT3G16630](http://www.arabidopsis.org/servlets/TairObject?type=locus&name=AT3G16630) | Kinesin-13A |
|  | B9RYP4 | 3.30 | 3.30 | 6.06 | [At4g10840](http://www.arabidopsis.org/servlets/TairObject?type=locus&name=AT4G10840) | Kinesin light chain |
| Small GTPase superfamily, RAB family |  |  |  |  |  |  |
|  | Q9FGK5 | 0 | 13.57 | 0 | At5g47520 | **RabA/Rab11 group (RabA5a)** |
|  | Q40193 | 0 | 21.60 | 0 | At1g07410 | **RabA/Rab11 group (RabA2b)** |
|  | B9H2F2 | 0 | 21.40 | 0 | At4g18800 | **RabA/Rab11 group (RabA1d, Rab11B)** |
|  | A9PCE2 | 12.40 | 27.90 | 0 | At1g09630 | **RabA/Rab11 group (RabA2a, Rab11C)** |
|  | B9GU86 | 0 | 55.29 | 0 | At2g31680 | RabA/Rab11 group (RabA5d) |
|  | B9RHG6 | 7.47 | 0 | 0 | At3g12160 | **RabA/Rab11 group (RabA4d)** |
|  | A5AWW7 | 0 | 22.42 | 0 | At5g47960 | RabA/Rab11 group (RabA4c) |
|  | D7TLL7 | 0 | 8.29 | 0 | At1g07410 | **RabA/Rab11 group (RabA2b)** |
|  | A5B3S9 | 23.25 | 0 | 0 | At1g05810 | RabA/Rab11 group (RabA5e, ARA1) |
|  | A5BGY6 | 0 | 48.67 | 0 | At5g65270 | RabA/Rab11 group (RabA4a) |
|  | P92963 | 17.37 | 45.81 | 0 | At4g17170 | **RabB/Rab2 group (RabB1b, RabB1c, Rab2A, Rab2)** |
|  | Q38922 | 50.55 | 75.82 | 0 | At4g35860 | **RabB/Rab2 group (RabB1b, Rab2C)** |
|  | B9SGV6 | 0 | 0 | 12.19 | At4g17170 | **RabB/Rab2 group (RabB1c, RabB1b, Rab2A)** |
|  | Q9LP15 | 24.32 | 0 | 0 | [At1g43890](http://www.arabidopsis.org/servlets/TairObject?type=locus&name=AT1G43890) | RabC/Rab18 group (RabC1, Rab18, RabB18) |
|  | D7TIU1 | 0 | 12.63 | 0 | At5g03530 | RabC/Rab18 group (RabC2A, Rab18b) |
|  | E0CQK6 | 7.60 | 68.40 | 0 | At1g43890 | RabC/Rab18 group (RabC1, Rab18-1) |
|  | Q8W4S8 | 0 | 13.20 | 0 | At4g17530 | **RabD/Rab1 group (RabD2C, Rab1C)** |
|  | B9MUT7 | 75.53 | 228.24 | 72.24 | At1g02130 | **RabD/Rab1 group (RabD2a, ARA5)** |
|  | D7TGR5 | 29.70 | 21.45 | 0 | At3g11730 | **RabD/Rab1 group (RabD1)** |
|  | P28186 | 72.53 | 103.39 | 15.43 | At3g46060 | **RabE/Rab8 group( RabE1c, Rab8A, ARA3)** |
|  | B9RAT6 | 0 | 14.49 | 0 | At4g20360 | **RabE/Rab8 group (RABE1b, RAB8D)** |
|  | P31582 | 115.00 | 131.66 | 35.00 | At5g45130 | **RabF/Rab5 group ( RabF2a)** |
|  | B9HUI6 | 26.53 | 36.48 | 21.55 | At3g54840 | **RabF/Rab5 group ( RabF1, ARA6)** |
|  | Q43463 | 0 | 58.25 | 0 | At4g09720 | RabG/Rab7 group (RabG3a) |
|  | Q9XER8 | 0 | 28.98 | 0 | At1g52280 | RabG/Rab7 group (RabG3d) |
|  | Q9LFT9 | 17.71 | 27.37 | 0 | At5g10260 | RabH/Rab6 group ( RabH1e) |
|  | O80501 | 76.92 | 54.48 | 0 | At2g44610 | **RabH/Rab6 group (RabH1b, Rab6A)** |
|  | B9S6U7 | 15.72 | 9.67 | 0 | At3g06540 | REP, RAB escort protein |
|  | Q9LTF8 | 0 | 5.91 | 0 | At5g52580 | RabGAP/TBC domain-containing protein, RAB GTPase activator |
|  | B9RWU9 | 3.04 | 4.87 | 0 | [At3g49350](http://www.arabidopsis.org/servlets/TairObject?type=locus&name=AT3G49350) | Ypt/Rab-GAP domain of gyp1p superfamily protein, RAB GTPase activator |
|  | B9SA43 | 0 | 7.26 | 0 | [At4g27100](http://www.arabidopsis.org/servlets/TairObject?type=locus&name=AT4G27100) | Ypt/Rab-GAP domain of gyp1p superfamily protein, RAB GTPase activator |
|  | B9SP88 | 0 | 4.04 | 0 | [At4g29950](http://www.arabidopsis.org/servlets/TairObject?type=locus&name=AT4G29950) | Ypt/Rab-GAP domain of gyp1p superfamily protein, RAB GTPase activator |
|  | D7TC51 | 0 | 3.89 | 3.41 | [At5g52580](http://www.arabidopsis.org/servlets/TairObject?type=locus&name=AT5G52580) | Ypt/Rab-GAP domain of gyp1p superfamily protein, RAB GTPase activator |
|  | D7SMJ1 | 0 | 5.58 | 0 | [At3g02460](http://www.arabidopsis.org/servlets/TairObject?type=locus&name=AT3G02460) | Ypt/Rab-GAP domain of gyp1p superfamily protein, RAB GTPase activator |
|  | A9PC79 | 30.03 | 80.33 | 16.51 | At2g44100 | GDI1, guanosine nucleotide diphosphate dissociation inhibitor 1 |
|  | B9SL15 | 36.19 | 93.51 | 27.14 | At2g38360 | PRA1.B4, prenylated rab acceptor 1.B4 |
|  | B9SZ60 | 7.71 | 10.80 | 3.08 | At1g08770 | PRA1.E, prenylated rab acceptor 1.E |
| Small GTPase superfamily, ARF family |  |  |  |  |  |  |
|  | Q9ZPX1 | 0 | 18.01 | 0 | [At2g18390](http://www.arabidopsis.org/servlets/TairObject?type=locus&name=AT2G18390) | ARL2, HAL, TTN5 |
|  | B9R9D6 | 37.58 | 251.63 | 58.82 | At5g52210 | GB1, GTP-binding protein 1 |
|  | D7T663 | 341.23 | 873.61 | 176.93 | [At3g62290](http://www.arabidopsis.org/servlets/TairObject?type=locus&name=AT3G62290) | ARF1E, ADP-ribosylation factor A1E |
|  | O04834 | 72.53 | 103.62 | 58.72 | At4g02080 | SAR2, secretion-associated RAS super family 2 |
| Small GTPase superfamily, RAN family |  |  |  |  |  |  |
|  | D7SUQ2 | 83.06 | 106.01 | 22.95 | At5g55190 | RAN3 |
|  | D7TBW7 | 0 | 0 | 20.51 | [At5g55190](http://www.arabidopsis.org/servlets/TairObject?type=locus&name=AT5G55190) | RAN3 |
|  | D7U0V8 | 83.64 | 57.42 | 0 | [At5g55190](http://www.arabidopsis.org/servlets/TairObject?type=locus&name=AT5G55190) | RAN3 |
| Small GTPase superfamily, RHO GTPase family |  |  |  |  |  |  |
|  | B9RF54 | 0 | 13.72 | 0 | [At3g63150](http://www.arabidopsis.org/servlets/TairObject?type=locus&name=AT3G63150) | Calcium binding GTP-ASE, MIRO-related GTP-ASE 2, MIRO2 |
|  | B9R9W7 | 0 | 16.26 | 0 | At5g61530 | Rho GTPase activator |
| Syntaxin/t-SNARE  family |  |  |  |  |  |  |
|  | A5BYQ4 | 0 | 37.71 | 0 | [At5g46860](http://www.arabidopsis.org/servlets/TairObject?type=locus&name=AT5G46860) | **SYP22, VAM3, SGR3** |
|  | B9SDJ9 | 0 | 28.48 | 0 | [At3g11820](http://www.arabidopsis.org/servlets/TairObject?type=locus&name=AT3G11820) | **SYP121, SYR, PEN1** |
|  | B9SIA7 | 0 | 27.41 | 0 | [At5g08080](http://www.arabidopsis.org/servlets/TairObject?type=locus&name=AT5G08080) | **SYP132** |
|  | B9RFG2 | 0 | 9.28 | 0 | At3g05710 | **SYP43** |
|  | B9T2S1 | 0 | 59.36 | 0 | [At5g06320](http://www.arabidopsis.org/servlets/TairObject?type=locus&name=AT5G06320) | NDR1/HIN1-LIKE 3, NHL3 |
|  | D7T4S5 | 0 | 5.06 | 0 | [At5g05760](http://www.arabidopsis.org/servlets/TairObject?type=locus&name=AT5G05760) | **SYP31, SED5** |
|  | Q94AU2 | 0 | 19.87 | 0 | At1g11890 | **SNARE protein SEC22, SECRETION 22** |
| Exocyst componenet |  |  |  |  |  |  |
|  | B9RRM3 | 6.15 | 21.98 | 0 | [At3g10380](http://www.arabidopsis.org/servlets/TairObject?type=locus&name=AT3G10380) | SEC8, subunit of exocyst complex 8 |
| VSR (BP-80) family |  |  |  |  |  |  |
|  | P93026 | 0 | 5.88 | 0 | At3g52850 | VSR1 |
| SCAMP family |  |  |  |  |  |  |
|  | Q9SXA5 | 0 | 6.87 | 0 | At1g11180 | SCAMP5 |
|  |  |  |  |  |  |  |
| Dynamin family |  |  |  |  |  |  |
|  | B9SBU7 | 0 | 4.05 | 0 | [At1g10290](http://www.arabidopsis.org/servlets/TairObject?type=locus&name=AT1G10290) | ADL6, DRP2A |
|  | B9T3E4 | 3.18 | 8.36 | 0 | At4g33650 | ADL2, DRP3A |
| SecY/SEC61-alpha family |  |  |  |  |  |  |
|  | B9T4L2 | 0 | 2.99 | 0 | At2g18710 | SCY1, SECY HOMOLOG 1 |
|  | D7T5Q9 | 0 | 14.41 | 0 | [At1g29310](http://www.arabidopsis.org/servlets/TairObject?type=locus&name=AT1G29310) | SecY |
| G-alpha family |  |  |  |  |  |  |
|  | P18064 | 0 | 0 | 22.62 | At2g26300 | GP ALPHA 1, GPA1 |
| GTP-binding elongation factor family |  |  |  |  |  |  |
|  | A5BSV9 | 0 | 13.62 | 0 | [At1g04170](http://www.arabidopsis.org/servlets/TairObject?type=locus&name=AT1G04170) | EIF2 GAMMA, eukaryotic translation initiation factor 2 |
|  | B9I879 | 0 | 15.36 | 0 | [At1g18070](http://www.arabidopsis.org/servlets/TairObject?type=locus&name=AT1G18070) | Translation elongation factor EF1A/initiation factor |
|  | B9PAE3 | 11.49 | 0 | 0 | [At5g60390](http://www.arabidopsis.org/servlets/TairObject?type=locus&name=AT5G60390) | GTP binding Elongation factor Tu family protein |
|  | O23755 | 134.44 | 653.22 | 157.37 | [At1g56070](http://www.arabidopsis.org/servlets/TairObject?type=locus&name=AT1G56070) | LOS1, Ribosomal protein S5/Elongation factor G/III/V |
|  | B9GTD8 | 5.96 | 0 | 0 | [At4g02930](http://www.arabidopsis.org/servlets/TairObject?type=locus&name=AT4G02930) | GTP binding Elongation factor Tu family protein |
|  | Q56XT1 | 0 | 14.59 | 0 | At1g62750 | Elongation factor G |
|  | D7TDD3 | 0 | 14.13 | 0 | [At1g62750](http://www.arabidopsis.org/servlets/TairObject?type=locus&name=AT1G62750) | SCO1, Translation elongation |
|  | B9DHZ8 | 13.93 | 116.14 | 61.55 | At4g20360 | Translation elongation factor |
|  | D7SX17 | 0 | 6.26 | 0 | [At1g17220](http://www.arabidopsis.org/servlets/TairObject?type=locus&name=AT1G17220) | FUG1, Translation initiation factor 2, small GTP-binding |
| V-type ATPase family |  |  |  |  |  |  |
|  | Q9SZN1 | 15.05 | 48.59 | 0 | At4g38510 | VHA-B2 |
|  | O23654 | 4.28 | 12.84 | 2.67 | At1g78900 | VHA-A |
|  | Q9XGM1 | 16.60 | 66.41 | 12.77 | At3g58730 | VHA-D, VATD, VATPD |
|  | P59227 | 140.24 | 95.52 | 18.29 | At4g34720;At4g38920 At2g16510 | VHA-C1 AVA-P1 AVAP1; VHA-C3 AVA-P3 AVAP3; VHA-C5 AVA-P5 AVAP5 |
|  | Q9SZY7 | 77.77 | 103.70 | 24.07 | At4g32530 | VMA16 |
|  | Q9LJI5 | 0 | 94.01 | 0 | At3g28710 | VHA-D1 |
