# Supplementary material for: Transcriptomic Events Involved in Melon Mature-Fruit Abscission Comprise the Sequential Induction of Cell-Wall Degrading Genes Coupled to a Stimulation of Endo and Exocytosis
Source: PLoS One. 2013 Mar 6;8(3):e58363. doi: 10.1371/journal.pone.0058363 (PMC3590154; doi:10.1371/journal.pone.0058363)
Supplement: Table S11 — Protein-phosphorylation-associated genes induced or repressed in fruit-AZ during melon MFA. Sequences were selected after establishing a P<0.01.The table shows the total read count in RPKMx1000 for each gene after normalization across the 3 samples: (a) AZ pre-cell separation (36 DPA), (b) AZ partial-cell separation (38 DPA), (c) almost complete-cell separation (40 DPA). (DOC) [file pone.0058363.s022.doc]

**Table S11** Protein-phosphorylation-associated genes induced or repressed in fruit-AZ during melon MFA. Sequences were selected after establishing a P<0.01.The table shows the total read count in RPKMx1000 for each gene after normalization across the 3 samples: (a) AZ pre-cell separation (36 DPA), (b) AZ partial-cell separation (38 DPA), (c) almost complete-cell separation (40 DPA).

| **UniProt ID** | **36 DPA** | **38 DPA** | **40 DPA** | **Description** |
| --- | --- | --- | --- | --- |
| D7SK82 | 0 | 65.30 | 40.00 | uncharacterized protein = *Vitis vinifera* |
| P48578 | 13.84 | 167.19 | 8.51 | Serine/threonine-protein phosphatase PP2A-3 catalytic subunit, PP2A3 EP7 PP2A4 At3g58500 F14P22.90 |
| D7SYX3 | 0 | 45.85 | 0 | uncharacterized protein = *Vitis vinifera* |
| B9S5U9 | 0 | 25.49 | 0 | Protein phosphatase 2c = *Ricinus communis* |
| B9IA45 | 0 | 19.80 | 0 | uncharacterized protein = *Populus trichocarpa* |
| D7T4F2 | 17.62 | 0 | 0 | uncharacterized protein = *Vitis vinifera* |
| D7TEG6 | 26.96 | 17.15 | 0 | uncharacterized protein = *Vitis vinifera* |
| B9SPE2 | 0 | 13.93 | 0 | protein tyrosine/serine/threonine phosphatase = |
| B9ILA9 | 0 | 26.25 | 0 | uncharacterized protein = *Populus trichocarpa* |
| D7SV50 | 11.20 | 77.49 | 16.80 | uncharacterized protein = *Vitis vinifera* |
| Q9M3V1 | 6.45 | 0 | 0 | Protein phosphatase 2C (PP2C) = *Fagus sylvatica* |
| B9R8Q9 | 0 | 16.36 | 0 | Protein phosphatase 2c = *Ricinus communis* |
| B9S5C5 | 0 | 16.84 | 0 | Protein phosphatase 2c = *Ricinus communis* |
| D7U2P0 | 0 | 16.07 | 0 | uncharacterized protein = *Vitis vinifera* |
| B9RNU7 | 5.54 | 0 | 0 | Protein phosphatase 2c = *Ricinus communis* |
| B9SUG2 | 0 | 111.94 | 0 | Guanylate kinase = *Ricinus communis* |
| B9SVD1 | 0 | 93.87 | 0 | ATP binding protein = *Ricinus communis* |
| D7SHG7 | 2.29 | 110.34 | 64.36 | uncharacterized protein = *Vitis vinifera* |
| B9SFH0 | 0 | 75.82 | 0 | Protein kinase atmrk1= *Ricinus communis* |
| D7T237 | 0 | 50.07 | 0 | uncharacterized protein = *Vitis vinifera* |
| B9RII2 | 0 | 48.04 | 0 | ATP binding protein= *Ricinus communis* |
| B9RIE7 | 0 | 54.51 | 14.41 | Poly(P)/ATP NAD kinase = *Ricinus communis* |
| B9S2A6 | 23.32 | 0 | 0 | AMP-activated protein kinase, gamma regulatory subunit = *Ricinus communis* |
| B9SEU2 | 10.49 | 0 | 0 | ATP binding protein = *Ricinus communis* |
| D7T6M9 | 0 | 17.19 | 2.83 | uncharacterized protein = *Vitis vinifera* |
| B9RLU0 | 0 | 24.56 | 9.09 | Brassinosteroid LRR receptor kinase = *Ricinus communis* |
| Q7GC82 | 19.88 | 5.75 | 0 | Cm-ERS1 protein = *Cucumis melo* |
| B9SU88 | 27.43 | 0 | 0 | Receptor for activated protein kinase C = *Ricinus communis* |
| Q9SSY6 | 0 | 33.78 | 0 | Ethylene receptor 1, ETR1 = *Cucumis sativus* |
| D7U5N9 | 40.81 | 15.87 | 11.33 | uncharacterized protein = *Vitis vinifera* |
| B9SHG2 | 0 | 31.67 | 7.09 | Casein kinase = *Ricinus communis* |
| Q9FDV7 | 0 | 44.44 | 4.16 | Protein kinase (PK) |
| A0MNJ1 | 5.30 | 90.90 | 4.54 | CBL-interacting protein kinase 3, CIPK3 = *Populus trichocarpa* |
| B9SWC3 | 0 | 49.64 | 9.45 | Calcium/calmodulin-dependent protein kinase kinase = *Ricinus communis* |
| B9REV9 | 0 | 35.02 | 0 | Serine/threonine-protein kinase cdk9 = *Ricinus communis* |
| B9SPP7 | 0 | 29.36 | 0 | Protein ABC1 = *Ricinus communis* |
| B9T544 | 9.09 | 124.24 | 100.00 | Fructokinase = *Ricinus communis* |
| D7SZI3 | 0 | 35.91 | 0 | uncharacterized protein = *Vitis vinifera* |
| B9RLG3 | 0 | 28.33 | 3.09 | Inositol or phosphatidylinositol kinase = *Ricinus communis* |
| B9RWX6 | 0.9 | 21.29 | 0 | Fkbp-rapamycin associated protein = *Ricinus communis* |
| O65379 | 11.03 | 0 | 0 | F12F1.13 protein |
| B9S1K5 | 0 | 39.56 | 7.32 | Mak = *Ricinus communis* |
| B9S7L3 | 0 | 34.78 | 0 | Casein kinase = *Ricinus communis* |
| B9SJ54 | 0 | 32.47 | 0 | Big map kinase/bmk = *Ricinus communis* |
| O23249 | 233.71 | 218.39 | 126.43 | Cyclin-dependent kinases regulatory subunit 1 CKS1 At2g27960 T1E2.12 |
| B9S2T6 | 7.91 | 0 | 0 | ATP binding protein = *Ricinus communis* |
| D7SVW4 | 0 | 36.09 | 0 | uncharacterized protein = *Vitis vinifera* |
| D7U323 | 0 | 35.54 | 0 | uncharacterized protein = *Vitis vinifera* |
| D7SU25 | 0 | 20.64 | 0 | uncharacterized protein = *Vitis vinifera* |
| A9P7U5 | 0 | 26.56 | 9.48 | Pyruvate kinase = *Populus trichocarpa* |
| B9RA37 | 5.46 | 85.24 | 22.95 | Inositol hexaphosphate kinase = *Ricinus communis* |
| A9P984 | 0 | 39.10 | 21.50 | uncharacterized protein = *Populus trichocarpa* |
| D7TCE8 | 16.59 | 6.78 | 0 | uncharacterized protein = *Vitis vinifera* |
| C4P7V9 | 10.70 | 0 | 0 | CBL-interacting protein kinase 10, CIPK10 = *Vitis vinifera* |
| D7TQF1 | 0 | 28.74 | 0 | uncharacterized protein = *Vitis vinifera* |
| B6V3C0 | 0 | 25.43 | 0 | Hexokinase 1 = *Cucumis melo* |
| D7SWH1 | 0 | 26.33 | 0 | uncharacterized protein = *Vitis vinifera* |
| D7TX82 | 2.65 | 0 | 0 | uncharacterized protein = *Vitis vinifera* |
| B9HSF8 | 4.32 | 56.27 | 0 | uncharacterized protein = *Populus trichocarpa* |
| B9SRC8 | 0 | 19.13 | 0 | Big map kinase/bmk = *Ricinus communis* |
| Q5F2M7 | 0 | 19.92 | 0 | Pyruvate kinase = *Glycine max* |
| Q6RUF8 | 0 | 19.26 | 0 | Glycerol kinase = *Glycine max* |
| B9RVC9 | 0 | 28.15 | 0 | Homoserine kinase = *Ricinus communis* |
| B9T4J2 | 0 | 35.02 | 82.48 | Casein kinase II, alpha chain = *Ricinus communis* |
| D7TRL4 | 0 | 30.57 | 0 | uncharacterized protein = *Vitis vinifera* |
| Q9SA26 | 0 | 9.88 | 4.35 | F3O9.7 protein, At1g16270 F3O9.7 |
| D7TWH5 | 0 | 33.14 | 0 | uncharacterized protein = *Vitis vinifera* |
| P43289 | 0 | 28.52 | 12.22 | Shaggy-related protein kinase gamma, ASK3 At3g05840 F10A16.14 |
| Q06060 | 0 | 24.53 | 0 | Mitogen-activated protein kinase homolog D5 = *Pisum sativum* |
| D7T3N9 | 0 | 3.96 | 1.68 | uncharacterized protein = *Vitis vinifera* |
| D7KVL5 | 0 | 11.90 | 0 | Kinase family protein = *Arabidopsis lyrata* |
| B9S8J6 | 0 | 19.98 | 2.14 | Calcium-dependent protein kinase = *Ricinus communis* |
| P93774 | 0 | 33.69 | 0 | Shaggy-like kinase = *Ricinus communis* |
| D7SWB3 | 0 | 19.51 | 0 | uncharacterized protein = *Vitis vinifera* |
| Q9LRN0 | 0 | 10.92 | 6.10 | AT3g24190/MUJ8_17 |
| A5BJJ8 | 0 | 17.31 | 0 | CBL-interacting protein kinase 07, CIPK07 = *Vitis vinifera* |
| D7SRF4 | 0 | 27.02 | 0 | uncharacterized protein = *Vitis vinifera* |
| D7TD38 | 0 | 9.62 | 0 | uncharacterized protein = *Vitis vinifera* |
| D7TEC1 | 0 | 12.80 | 0 | uncharacterized protein = *Vitis vinifera* |
| A0MNJ4 | 0 | 18.98 | 9.87 | CBL-interacting protein kinase 7 = *Populus trichocarpa* |
| B9N4U7 | 0 | 22.95 | 0 | uncharacterized protein = *Populus trichocarpa* |
| B9RUI5 | 0 | 13.84 | 4.42 | BRASSINOSTEROID INSENSITIVE 1-associated receptor kinase 1 = *Ricinus communis* |
| B9SNC0 | 0 | 39.30 | 7.86 | Serine-threonine protein kinase = *Ricinus communis* |
| B9RBD2 | 5.37 | 49.05 | 7.39 | Protein-tyrosine kinase = *Ricinus communis* |
| B9S423 | 25.87 | 13.69 | 0 | Cyclin-dependent protein kinase = *Ricinus communis* |
| B9RKE5 | 4.74 | 0 | 0 | Phosphofructokinase = *Ricinus communis* |
| D7SJ26 | 3.08 | 0 | 0 | uncharacterized protein = *Vitis vinifera* |
| B9SDF7 | 0 | 9.04 | 0 | ATP binding protein = *Ricinus communis* |
| B9SFF2 | 0 | 9.03 | 2.35 | S-locus-specific glycoprotein S6 = *Ricinus communis* |
| P42066 | 3.48 | 32.33 | 4.97 | Phosphoenolpyruvate carboxykinase [ATP] (PEP carboxykinase) (PEPCK) = *Cucumis sativus* |
| A9PGT2 | 0 | 27.46 | 0 | Phosphoribulokinase = *Populus trichocarpa* |
| B9S8F8 | 0 | 7.97 | 0 | ATP binding protein = *Ricinus communis* |
| B9SR58 | 0 | 12.10 | 0 | Big map kinase/bmk = *Ricinus communis* |
| D7SK53 | 0 | 14.96 | 0 | uncharacterized protein = *Vitis vinifera* |
| Q9XF95 | 0 | 19.92 | 0 | Mitogen-activated protein kinase MAPK = *Prunus armeniaca* |
| D7TT82 | 14.15 | 9.43 | 57.38 | uncharacterized protein = *Vitis vinifera* |
| A5BWT7 | 2.08 | 26.64 | 0 | uncharacterized protein = *Vitis vinifera* |
| A8QYK9 | 48.67 | 85.61 | 13.90 | Ethylene receptor, ETR2 =*Cucumis melo* |
| A5BEQ2 | 3.98 | 0 | 0 | uncharacterized protein = *Vitis vinifera* |
| B9SUR2 | 4.65 | 0 | 0 | Mitogen activated protein kinase kinase kinase 3, mapkkk3, mekk3 = *Ricinus communis* |
| B9SZM1 | 7.93 | 0 | 0 | Protein kinase APK1A, chloroplast = *Ricinus communis* |
| Q42896 | 8.13 | 0 | 0 | Fructokinase-2 = *Solanum lycopersicum* |
| B9RNW5 | 0 | 15.11 | 68.02 | CBL-interacting serine/threonine-protein kinase = *Ricinus communis* |
| B9RVE0 | 0 | 15.54 | 10.10 | CBL-interacting serine/threonine-protein kinase = *Ricinus communis* |
| B9S2B9 | 0 | 13.46 | 0 | Protein kinase APK1B, chloroplast = *Ricinus communis* |
| B9SP66 | 0 | 16.22 | 0 | Serine/threonine-protein kinase PBS1= *Ricinus communis* |
| B9T2U0 | 0 | 12.50 | 0 | Calcium-dependent protein kinase = *Ricinus communis* |
| B9T5H7 | 0 | 15.50 | 0 | Serine/threonine protein kinase = *Ricinus communis* |
| D7KYV4 | 0 | 7.41 | 0 | Kinase family protein = *Arabidopsis lyrata* |
| D7T9M5 | 0 | 5.38 | 3.76 | uncharacterized protein = *Vitis vinifera* |
| D7TBN3 | 0 | 10.25 | 3.58 | uncharacterized protein = *Vitis vinifera* |
| D7TUS1 | 0 | 11.45 | 0 | uncharacterized protein = *Vitis vinifera* |
| A5C3G5 | 0 | 8.20 | 0 | uncharacterized protein = *Vitis vinifera* |
| B9SUI6 | 0 | 18.13 | 0 | Mevalonate kinase, putative (EC 2.7.1.36) |
| B9T2T2 | 0 | 7.28 | 0 | Serine/threonine-protein kinase PBS1 = *Ricinus communis* |
| Q9ZST3 | 0 | 12.36 | 0 | Pyrophosphate-dependent phosphofructokinase beta subunit = *Citrus paradisi* |
| D7U4C1 | 0 | 8.72 | 0 | uncharacterized protein = *Vitis vinifera* |
| D7U4E2 | 0 | 10.55 | 0 | uncharacterized protein = *Vitis vinifera* |
| B9STP9 | 40.00 | 178.09 | 40.00 | Serine-threonine kinase receptor-associated protein = *Ricinus communis* |
| B9S411 | 7.33 | 0 | 3.14 | WD-repeat protein = *Ricinus communis* |
| D7TM75 | 7.02 | 0 | 0 | uncharacterized protein = *Vitis vinifera* |
| Q56E62 | 15.76 | 0 | 0 | Nucleoside diphosphate kinase 1 = *Nicotiana tabacum* |
| Q9LKJ2 | 5.81 | 0 | 0 | Phosphoglycerate kinase = *Pisum sativum* |
| B9RTM1 | 7.63 | 57.67 | 16.11 | Protein kinase atmrk1 = *Ricinus communis* |
| A5C4V0 | 0 | 15.31 | 0 | uncharacterized protein = *Vitis vinifera* |
| A5CB35 | 0 | 15.39 | 0 | uncharacterized protein = *Vitis vinifera* |
| B9RCG7 | 0 | 14.64 | 0 | Big map kinase/bmk = *Ricinus communis* |
| C4P7W8 | 0 | 14.45 | 0 | CBL-interacting protein kinase 19 = *Vitis vinifera* |
| D7T8U3 | 0 | 3.80 | 0 | uncharacterized protein = *Vitis vinifera* |
| D7TNE7 | 0 | 11.78 | 0 | uncharacterized protein = *Vitis vinifera* |
| A5AW40 | 0 | 10.69 | 0 | uncharacterized protein = *Vitis vinifera* |
| A5AZP7 | 0 | 6.73 | 0 | uncharacterized protein = *Vitis vinifera* |
| B7FLG3 | 0 | 12.00 | 0 | Pyruvate kinase = *Medicago truncatula* |
| B9R7Y2 | 0 | 8.63 | 0 | Inositol-pentakisphosphate 2-kinase = *Ricinus communis* |
| B9RQY5 | 0 | 13.39 | 17.85 | Protein kinase APK1A, chloroplast = *Ricinus communis* |
| B9S8Q9 | 0 | 8.55 | 0 | Kinase = *Ricinus communis* |
| B9T3P6 | 0 | 16.34 | 0 | Protein kinase atn1 = *Ricinus communis* |
| B9RMT1 | 4.56 | 0 | 0 | F-box and wd40 domain protein = *Ricinus communis* |
| B9S652 | 2.95 | 0 | 0 | Serine/threonine protein kinase = *Ricinus communis* |
| Q9CAR7 | 6.99 | 0 | 0 | Hypersensitive-induced response protein 2, HIR2 At1g69840 T17F3.13 |
| Q9FM19 | 6.99 | 0 | 0 | Hypersensitive-induced response protein 1, HIR1 P31 At5g62740 MQB2.6 |
| Q9XGZ2 | 1.99 | 0 | 0 | Leucine-rich repeat receptor-like protein kinase, LRR-RLK At5g25930 AT5G25930 T1N24.22 |
| B9T650 | 0 | 12.46 | 0 | CBL-interacting serine/threonine-protein kinase = *Ricinus communis* |
| D7KMK3 | 0 | 5.84 | 0 | Kinase family protein = *Arabidopsis lyrata* |
| D7SIH7 | 0 | 13.16 | 0 | uncharacterized protein = *Vitis vinifera* |
| Q6QLL5 | 0 | 7.58 | 0 | WAK-like kinase = *Solanum lycopersicum* |
| B9SC81 | 9.31 | 5.58 | 0 | ATP binding protein = *Ricinus communis* |
| A5AHG4 | 0 | 10.61 | 0 | uncharacterized protein = *Vitis vinifera* |
| A5C8S4 | 0 | 4.86 | 0 | uncharacterized protein = *Vitis vinifera* |
| B9HDG3 | 0 | 6.49 | 0 | uncharacterized protein = *Populus trichocarpa* |
| B9REV1 | 0 | 4.36 | 0 | Nucleotide binding protein = *Ricinus communis* |
| B9RMJ0 | 0 | 13.77 | 27.54 | Adenylate kinase 1 = *Ricinus communis* |
| B9RYA6 | 0 | 8.63 | 0 | Serine-threonine protein kinase = *Ricinus communis* |
| B9SC89 | 0 | 6.54 | 0 | BRASSINOSTEROID INSENSITIVE 1-associated receptor kinase 1 = *Ricinus communis* |
| B9SGT0 | 0 | 4.37 | 0 | Sensory transduction histidine kinase = *Ricinus communis* |
| B9SQH2 | 0 | 3.17 | 1.26 | Phytosulfokine receptor = *Ricinus communis* |
| B9SXH2 | 0 | 5.60 | 0 | Kinase = *Ricinus communis* |
| B9T7T7 | 0 | 8.56 | 0 | Protein kinase APK1B = *Ricinus communis* |
| D7SHT8 | 0 | 9.55 | 0 | uncharacterized protein = *Vitis vinifera* |
| D7SLB5 | 0 | 2.43 | 0 | uncharacterized protein = *Vitis vinifera* |
| D7SXG2 | 0 | 6.48 | 0 | uncharacterized protein = *Vitis vinifera* |
| D7TZI8 | 0 | 4.70 | 0 | uncharacterized protein = *Vitis vinifera* |
| D7U816 | 0 | 7.57 | 0 | uncharacterized protein = *Vitis vinifera* |
| D7SNZ6 | 8.36 | 6.57 | 0 | uncharacterized protein = *Vitis vinifera* |
| Q9LLT5 | 0 | 0 | 7.54 | Receptor-like protein kinase, RLK = *Prunus dulcis* |
| B9HPN3 | 15.33 | 44.83 | 10.02 | Calcium-dependent protein kinase, CPK6, ATCDPK3, ATCPK6 , AT2G17290 |
| B9RL17 | 0 | 2.88 | 0 | Calcium-dependent protein kinase, CPK2, ATCPK2 , AT3G10660 |
| B9RNW5 | 0 | 15.11 | 68.02 | CBL-interacting serine/threonine-protein kinase, CIPK25, SnRK3.25, [AT5G25110](http://www.arabidopsis.org/servlets/TairObject?type=locus&name=AT5G25110) |
| B9RVE0 | 0 | 15.54 | 10.10 | CBL-interacting serine/threonine-protein kinase, CIPK11, PKS5, SIP4, SNRK3.22, [AT2G30360](http://www.arabidopsis.org/servlets/TairObject?type=locus&name=AT2G30360) |
| B9RVE7 | 0 | 5.10 | 0 | CBL-interacting serine/threonine-protein kinase, CIPK10, PKS2, SIP1, SNRK3.8m, [AT5G58380](http://www.arabidopsis.org/servlets/TairObject?type=locus&name=AT5G58380) |
| B9RW88 | 0 | 10.69 | 0 | Calcium-dependent protein kinase, CPK4, ATCPK4 , [AT4G09570](http://www.arabidopsis.org/servlets/TairObject?type=locus&name=AT4G09570) |
| B9RXL5 | 5.44 | 20.56 | 0 | Calcium-dependent protein kinase, CPK4, ATCPK4 , [AT4G09570](http://www.arabidopsis.org/servlets/TairObject?type=locus&name=AT4G09570) |
| B9S8J6 | 0 | 9.38 | 0 | Calcium-dependent protein kinase, CPK4, ATCPK4, AT4G09570 |
| B9S009 | 0 | 4.58 | 0 | Calcium-dependent protein kinase, CIPK1, SnRK3.16, [AT3G17510](http://www.arabidopsis.org/servlets/TairObject?type=locus&name=AT3G17510) |
| B9SLE6 | 0 | 9.38 | 0 | ATCDPK2, CPK11, ATCPK11, CDPK2, [AT1G35670](http://www.arabidopsis.org/servlets/TairObject?type=locus&name=AT1G35670) |
| B9SMX5 | 0 | 6.72 | 0 | CBL-interacting serine/threonine-protein kinase, CIPK8, SnRK3.13, PKS11, ATCIPK8, [AT4G24400](http://www.arabidopsis.org/servlets/TairObject?type=locus&name=AT4G24400) |
| B9SUF1 | 0 | 5.04 | 0 | Calcium-dependent protein kinase, CPK32, ATCPK32, CDPK32, [AT3G57530](http://www.arabidopsis.org/servlets/TairObject?type=locus&name=AT3G57530) |
| B9SWC3 | 0 | 49.64 | 9.45 | Calcium/calmodulin-dependent protein kinase kinase, GRIK2, ATSNAK1, [AT5G60550](http://www.arabidopsis.org/servlets/TairObject?type=locus&name=AT5G60550) |
| B9T2U0 | 0 | 12.50 | 0 | Calcium-dependent protein kinase, CPK33, [AT1G50700](http://www.arabidopsis.org/servlets/TairObject?type=locus&name=AT1G50700) |
| B9T650 | 0 | 12.46 | 0 | CBL-interacting serine/threonine-protein kinase, CIPK10, PKS2, SIP1, SNRK3.8, [AT5G58380](http://www.arabidopsis.org/servlets/TairObject?type=locus&name=AT5G58380) |
| Q7XJR9 | 0 | 2.91 | 0 | Calcium-dependent protein kinase, CPK16, [AT2G17890](http://www.arabidopsis.org/servlets/TairObject?type=locus&name=AT2G17890) |
