# Supplementary material for: Transcriptomic Events Involved in Melon Mature-Fruit Abscission Comprise the Sequential Induction of Cell-Wall Degrading Genes Coupled to a Stimulation of Endo and Exocytosis
Source: PLoS One. 2013 Mar 6;8(3):e58363. doi: 10.1371/journal.pone.0058363 (PMC3590154; doi:10.1371/journal.pone.0058363)
Supplement: Table S12 — Ethylene- and polyamine-related genes induced or repressed in fruit-AZ during melon MFA. Sequences were selected after establishing a P<0.01.The table shows the total read count in RPKMx1000 for each gene after normalization across the 3 samples: (a) AZ pre-cell separation (36 DPA), (b) AZ partial-cell separation (38 DPA), (c) almost complete-cell separation (40 DPA). (DOC) [file pone.0058363.s023.doc]

**Table S12** Ethylene- and polyamine-related genes induced or repressed in fruit-AZ during melon MFA. Sequences were selected after establishing a P<0.01.The table shows the total read count in RPKMx1000 for each gene after normalization across the 3 samples: (a) AZ pre-cell separation (36 DPA), (b) AZ partial-cell separation (38 DPA), (c) almost complete-cell separation (40 DPA).

| **UniProt ID** | **36 DPA** | **38 DPA** | **40 DPA** | **Description** |
| --- | --- | --- | --- | --- |
| ***Ethylene*** |  |  |  |  |
| B9S1K1 | 0 | 17.29 | 0 | S-adenosylmethionine-dependent methyltransferase = *Ricinus communis* |
| A7Q0V4 | 23.01 | 138.10 | 74.16 | S-adenosylmethionine synthase 5 (AdoMet synthase 5) (EC 2.5.1.6) (Methionine adenosyltransferase 5) =*Vitis vinifera* |
| B9I615 | 30.50 | 144.66 | 51.41 | Methionine synthase 2= *Populus trichocarpa* |
| A9P822 | 751.70 | 900.51 | 663.26 | S-adenosylmethionine synthase 1 (AdoMet synthase 1) (EC 2.5.1.6) (Methionine adenosyltransferase 1) (MAT 1) = *Populus trichocarpa* |
| B9SBC0 | 9.63 | 128.51 | 11.24 | 1-aminocyclopropane-1-carboxylate synthase. ACS = *Ricinus communis* |
| Q45RS3 | 0 | 59.22 | 19.28 | 1-aminocyclopropane-1-carboxylate synthase. ACS = *Vitis vinifera* |
| B9RBW1 | 0 | 6.59 | 0 | 1-aminocyclopropane-1-carboxylate synthase = *Ricinus communis* |
| O82123 | 0 | 10.14 | 0 | 1-aminocyclopropane-1-carboxylate synthase ACS1 = *Cucumis sativus* |
| D7T1X0 | 0 | 3.77 | 0 | 1-aminocyclopropane-1-carboxylate synthase. ACS = *Vitis vinifera* |
| Q9LN15 | 0 | 0 | 4.97 | 1-aminocyclopropane-1-carboxylate synthase ACS8 = *Arabidopsis thaliana.* T13M22.3 |
| D7TZK0 | 11.06 | 48.40 | 27.66 | 1-aminocyclopropane-1-carboxylate synthase = *Vitis vinifera* |
| B9H7C6 | 45.19 | 99.27 | 8.07 | 1-aminocyclopropane-1-carboxylate synthase = *Populus trichocarpa* |
| C7U1K1 | 24.41 | 151.80 | 0 | 1-aminocyclopropane-1-carboxylate oxidase = *Cucumis sativus* |
| O48631 | 0 | 39.27 | 0 | 1-aminocyclopropane-1-carboxylate oxidase = *Prunus armeniaca* |
| Q04644 | 26.20 | 31.44 | 0 | 1-aminocyclopropane-1-carboxylate oxidase 1 (ACC oxidase 1) (EC 1.14.17.4) (Ethylene-forming enzyme) (EFE) (PMEL1) = *Cucumis melo* |
| B9RTK2 | 236.66 | 356.66 | 196.66 | 1-aminocyclopropane-1-carboxylate oxidase . ACO = *Ricinus communis* |
| B9RYX6 | 16.37 | 0 | 8.67 | 1-aminocyclopropane-1-carboxylate oxidase = *Ricinus communis* |
| Q9C5K7 | 10.84 | 0 | 0 | 1-aminocyclopropane-1-carboxylate oxidase 2. ACO2. At1g06640 F12K11.27 F12K11.6 |
| B9RKA0 | 0 | 0 | 12.98 | 1-aminocyclopropane-1-carboxylate oxidase = *Ricinus communis* |
| Q9SSY6 | 0 | 33.78 | 0 | Ethylene receptor 1 (EC 2.7.13.3) (CS-ETR1) = *Cucumis sativus* |
| A8QYK9 | 48.67 | 85.61 | 13.90 | Ethylene receptor. ETR2 =*Cucumis melo* |
| D1M862 | 0 | 1.53 | 0 | Ethylene insensitive 2. EIN2 =*Prunus persica* |
| Q948P4 | 10.10 | 41.36 | 11.06 | CmEIL1 = *Cucumis melo* |
| Q948P3 | 3.25 | 10.84 | 7.04 | CmEIL2 = *Cucumis melo* |
| Q70AB2 | 0 | 5.29 | 0 | Ethylene transcription factor. ERF1 = *Fagus sylvatica* |
| Q3L0R1 | 6.83 | 10.25 | 0 | Ethylene-responsive element binding protein ERF2 = *Gossypium hirsutum* |
| Q2I2S8 | 230.88 | 821.10 | 62.69 | Ethylene-responsive element-binding protein. ERF-6 = *Medicago truncatula* |
| D8VD38 | 0 | 1680.00 | 0 | Ethylene response factor 11. ERF-11 = *Actinidia deliciosa* |
| Q8GWK2 | 0 | 3.11 | 0 | AP2-like ethylene-responsive transcription factor At2g41710 |
| Q9LTC4 | 0 | 22.22 | 0 | MYB transcription factor 15. At3g23250 |
| Q38950 | 4.54 | 31.80 | 0 | Serine/threonine-protein phosphatase 2A 65 kDa regulatory subunit A beta isoform (AtA beta) (PP2A. subunit A. beta isoform). PP2AA2 DF1 At3g25800 |
| P28186 | 72.53 | 103.39 | 15.43 | Ras-related protein ARA-3. ARA-3. At3g46060 F12M12_30 |
| Q6NPP4 | 1.90 | 6.03 | 4.21 | Calmodulin-binding transcription activator 2 (Ethylene-induced calmodulin-binding protein c) (AtER66) (EICBP.c) (Signal-responsive protein 4). CMTA2 SR4 At5g64220 MSJ1.6 |
| B9RYX6 | 16.37 | 0 | 8.67 | 1-aminocyclopropane-1-carboxylate oxidase. putative (EC 1.14.11.9) = *Ricinus communis* |
| Q9C5K7 | 10.84 | 0 | 0 | 1-aminocyclopropane-1-carboxylate oxidase homolog 2. At1g06640 F12K11.27 F12K11.6 |
| Q7GC82 | 19.88 | 5.75 | 5.75 | Cm-ERS1 protein (Ethylene receptor) *= Cucumis melo* |
| Q5S004 | 579.94 | 192.41 | 75.88 | Ethylene response factor 1. ERF1 =*Cucumis sativus* |
| B9S948 | 55.87 | 40.46 | 0 | Ethylene-responsive transcription factor ERF. putative = *Ricinus communis* |
| B9SZX0 | 49.10 | 0 | 0 | Ethylene-responsive transcription factor ERF1B. putative = *Ricinus communis* |
| B9STH2 | 8.83 | 0 | 0 | Ethylene-responsive transcription factor. putative = *Ricinus communis* |
| Q38882 | 4.52 | 0 | 0 | Phospholipase D alpha 1 (AtPLDalpha1) (PLD alpha 1) (EC 3.1.4.4) (Choline phosphatase 1) (PLDalpha) (Phosphatidylcholine-hydrolyzing phospholipase D 1). PLDALPHA1 PLD1 At3g15730 MSJ11.13 |
| Q6V5J8 | 79.28 | 45.30 | 22.65 | AP2 transcription factor/ethylene response element= *Brassica oleracea* |
| B9T6G3 | 55.34 | 15.44 | 27.02 | ERF/AP2 domain transcription factor RAP2.3. putative = *Ricinus communis* |
| Q9AYR8 | 68.96 | 19.70 | 0 | Putative MADS-box protein. ERAF17 =*Cucumis sativus* |
| B9STH2 | 8.83 | 0 | 0 | Ethylene-responsive transcription factor. putative = *Ricinus communis* |
| Q9FHW7 | 25.34 | 0 | 38.98 | SKP1-like protein 1B (SKP1-like 2) (UFO-binding protein 2). SKP1B ASK2 UIP2 At5g42190 MJC20.30 |
| ***Polyamine*** |  |  |  |  |
| B3Y023 | 38.16 | 227.12 | 57.01 | Arginine decarboxylase (EC 4.1.1.19) = *Prunus persica* |
| B9SIY7 | 35.28 | 437.32 | 51.99 | S-adenosylmethionine decarboxylase proenzyme (EC 4.1.1.50) = *Ricinus communis* |
| Q6KC47 | 18.33 | 207.11 | 0 | S-adenosylmethionine decarboxylase (Fragment) = *Prunus persica* |
| Q9ZUB3 | 21.95 | 30.93 | 8.98 | Spermidine synthase 1 (SPDSY 1) (EC 2.5.1.16) = SPDSYN1 At1g23820 F5O8.38 |
| Q5F307 | 15.08 | 0 | 0 | Arginine decarboxylase (EC 4.1.1.19). ADC1 = *Solanum lycopersicum* |
| B9MYV7 | 15.50 | 0 | 7.75 | N-carbamoylputrescine amidase = *Populus trichocarpa* |
| A5BHF8 | 74.54 | 68.15 | 21.29 | Spermidine/spermine synthase family = *Vitis vinifera* |
| B9RIK2 | 7.24 | 0 | 0 | Spermidine synthase 1 = *Ricinus communis* |
