# Supplementary material for: Transcriptomic Events Involved in Melon Mature-Fruit Abscission Comprise the Sequential Induction of Cell-Wall Degrading Genes Coupled to a Stimulation of Endo and Exocytosis
Source: PLoS One. 2013 Mar 6;8(3):e58363. doi: 10.1371/journal.pone.0058363 (PMC3590154; doi:10.1371/journal.pone.0058363)
Supplement: Table S13 — Auxin- and ABA-related genes induced or repressed in fruit-AZ during melon MFA. Sequences were selected after establishing a P<0.01.The table shows the total read count in RPKMx1000 for each gene after normalization across the 3 samples: (a) AZ pre-cell separation (36 DPA), (b) AZ partial-cell separation (38 DPA), (c) almost complete-cell separation (40 DPA). (DOC) [file pone.0058363.s024.doc]

**Table S13** Auxin- and ABA-related genes induced or repressed in fruit-AZ during melon MFA. Sequences were selected after establishing a P<0.01.The table shows the total read count in RPKMx1000 for each gene after normalization across the 3 samples: (a) AZ pre-cell separation (36 DPA), (b) AZ partial-cell separation (38 DPA), (c) almost complete-cell separation (40 DPA).

| **UniProt ID** | **36 DPA** | **38 DPA** | **40 DPA** | **Description** |
| --- | --- | --- | --- | --- |
| ***Auxin*** |  |  |  |  |
| D7SQJ6 | 0 | 20.75 | 0 | Tryptophan synthase = *Vitis vinifera* |
| B9T4Y7 | 0 | 2.85 | 0 | Aldehyde dehydrogenase = *Ricinus communis* |
| B9S6G9 | 0 | 12.21 | 6.78 | Amine oxidase = *Ricinus communis* |
| B9GVN2 | 0 | 15.98 | 0 | Iaa-amino acid hydrolase 11 = *Populus trichocarpa* |
| O04951 | 0 | 70.57 | 0 | Serine/threonine-protein phosphatase PP2A-5 catalytic subunit (EC 3.1.3.16) (Protein phosphatase 2A isoform 5). PP2A5 At1g69960 F20P5.30 T17F3.1. Regulation of auxin polar transport |
| Q2UZS4 | 0 | 12.08 | 0 | Auxin influx carrier-like protein 3. AUX1/ AIC3 = *Momordica charantia* |
| B8Y9B4 | 0 | 16.98 | 8.78 | Transport inhibitor response protein. TIR1. auxin receptor = *Poncirus trifoliate* |
| Q9SSY2 | 17.84 | 92.01 | 96.71 | Aux/IAA protein. CsIAA2 = *Cucumis sativus* |
| D7U0A4 | 0 | 10.98 | 0 | Aux/IAA protein = *Vitis vinífera* |
| Q94JM3 | 0 | 2.32 | 0 | Auxin response factor 2 (ARF1-binding protein) (ARF1-BP) (Protein MEGAINTEGUMENTA). ARF2 MNT At5g62000 MTG10.3 |
| Q6L8U3 | 0 | 1.23 | 0 | Auxin response factor 1. CsARF1 = *Cucumis sativus* |
| Q6L8U1 | 0 | 2.18 | 0 | Auxin response factor 3. CsARF3 = *Cucumis sativus* |
| D7UCV0 | 0 | 3.84 | 0 | Auxin response factor. ARF = *Vitis vinifera* |
| D7SMW0 | 0 | 3.11 | 0 | Auxin response factor. ARF = *Vitis vinifera* |
| B9RA75 | 0 | 5.13 | 0 | Auxin response factor. putative = *Ricinus communis* |
| B9IBX4 | 0 | 35.29 | 11.76 | SAUR family protein. SAUR21 = *Populus trichocarpa* |
| O81829 | 0 | 10.89 | 0 | Indole-3-acetic acid-amido synthetase GH3.5 (EC 6.3.2.-) (Auxin-responsive GH3-like protein 5) (AtGH3-5) . GH3.5 At4g27260 M4I22.70 |
| O82333 | 0 | 14.12 | 0 | indole-3-acetic acid-amido synthetase GH3.1 (EC 6.3.2.-) (Auxin-responsive GH3-like protein 1) (AtGH3-1). GH3.1 At2g14960 T26I20.12 |
| B9RUC8 | 0 | 18.88 | 0 | Auxin-induced protein 5NG4. putative = *Ricinus communis* |
| B9T181 | 6.92 | 59.74 | 5.19 | Auxin-induced in root cultures protein 12. putative = *Ricinus communis* |
| B9T5V3 | 0 | 19.60 | 19.60 | Auxin-induced protein 5NG4. putative = *Ricinus communis* |
| D1MWZ6 | 427.77 | 494.44 | 308.33 | Auxin-repressed protein. CitAuR = *Citrullus lanatus* |
| Q9LTC4 | 0 | 22.22 | 0 | MYB transcription factor. At3g23250 |
| Q71T11 | 3.68 | 0 | 0 | Auxin efflux carrier. AEC3 = *Momordica charantia* |
| D7KJ74 | 18.99 | 0 | 0 | Auxin efflux carrier family protein . ARALYDRAFT_313001 = Arabidopsis lyrata subsp. Lyrata |
| A9PH58 | 20.16 | 12.09 | 0 | Aux/IAA protein = *Populus trichocarpa* |
| B9RTF5 | 20.94 | 0 | 0 | Aux/IAA protein. IAA4= *Ricinus communis* |
| D7TBU1 | 12.34 | 0 | 0 | Aux/IAA protein = *Vitis vinifera* |
| D7U0G0 | 13.69 | 0 | 5.70 | Aux/IAA protein = *Vitis vinifera* |
| A4PSF1 | 1.99 | 0 | 0 | Auxin response factor ARF: Transcriptional factor B3 = *Medicago truncatula* |
| Q03666 | 78.43 | 42.23 | 95.02 | Glutathione S-transferase (EC 2.5.1.18) (Auxin-induced protein PCNT107) = *Nicotiana tabacum* |
| Q940X7 | 121.46 | 107.34 | 45.19 | RING-box protein 1a (At-Rbx1;1) (Protein RING of cullins 1) (RBX1-2) (RBX1a-At). Cul3-RING ubiquitin ligase complex ( SCF ubiquitin ligase complex). RBX1A ROC1 At5g20570 F7C8.160 |
| Q9FHW7 | 25.34 | 0 | 38.98 | SKP1-like protein 1B (SKP1-like 2) (UFO-binding protein 2). Ubl conjugation pathway. SKP1B ASK2 UIP2 At5g42190 MJC20.30 |
| O04331 | 27.67 | 10.83 | 21.66 | Prohibitin (Prohibitin 3). Atphb3 At5g40770/K1B16.2 At5g40770 |
| D7U0G0 | 13.69 | 0 | 5.70 | Aux/IAA protein = *Vitis vinifera* |
| ***ABA*** |  |  |  |  |
| B9RZD3 | 0 | 27.89 | 0 | 4-hydroxy-3-methylbut-2-enyl diphosphate reductase. putative (EC 1.17.1.2) . IspH = *Ricinus communis* |
| A8WBX7 | 0 | 3.15 | 0 | Mevalonate disphosphate decarboxylase. MDC = *Solanum lycopersicum* |
| Q8RVK7 | 10.72 | 29.23 | 6.82 | Farnesyl pyrophosphate synthase. FPPS = *Malus domestica* |
| D7U5I9 | 5.64 | 10.49 | 0 | Farnesyl-diphosphate farnesyltransferase = *Vitis vinifera* |
| Q94ID7 | 0 | 10.81 | 0 | Geranylgeranyl pyrophosphate synthase. chloroplastic (GGPP synthase) = *Hevea brasiliensis* |
| P49293 | 0 | 41.86 | 0 | Phytoene synthase. chloroplastic (EC 2.5.1.32) (MEL5). PSY = *Cucumis melo* |
| D3JN88 | 0 | 39.27 | 5.30 | Beta-carotene hydroxylase. CrtR-b2 = *Solanum phureja x Solanum tuberosum* |
| O81360 | 0 | 9.07 | 0 | Zeaxanthin epoxidase. chloroplastic (EC 1.14.13.90) (PA-ZE) = *Prunus armeniaca* |
| B9S0Z6 | 0 | 40.08 | 0 | 9-cis-epoxycarotenoid dioxygenase. putative = *Ricinus communis* |
| Q9M6E8 | 0 | 4.87 | 0 | 9-cis-epoxycarotenoid dioxygenase NCED1. chloroplastic (EC 1.13.11.51) (PvNCED1). NCED1 = *Phaseolus vulgaris* |
| A0SE37 | 0 | 4.95 | 0 | 9-cis-epoxycarotenoid dioxygenase 3. NCED3 = *Citrus clementina* |
| A0SE34 | 0 | 5.47 | 0 | 9-cis-epoxycarotenoid dioxygenase 5. NCED5 = *Citrus clementina* |
| B9SBK0 | 0 | 21.50 | 0 | Abscisic acid receptor PYR/PYL = *Ricinus communis* |
| B9ILA9 | 0 | 26.25 | 0 | Protein phpsphatase 2C (PP2C) = *Populus trichocarpa* |
| B9R8Q9 | 0 | 16.36 | 0 | Protein phpsphatase 2C (PP2C) = *Ricinus communis* |
| B9S5C5 | 0 | 16.84 | 0 | Protein phpsphatase 2C (PP2C) = *Ricinus communis* |
| B9S5U9 | 0 | 25.49 | 0 | Protein phpsphatase 2C (PP2C) = *Ricinus communis* |
| B9RIK1 | 0 | 3.78 | 0 | Protein phpsphatase 2C (PP2C) = *Ricinus communis* |
| B9RNW5 | 0 | 15.11 | 68.02 | SnRK2 putative = *Ricinus communis* |
| B9RVE0 | 0 | 15.54 | 10.10 | SnRK2 putative = *Ricinus communis* |
| B9T650 | 0 | 12.46 | 0 | SnRK2 putative = *Ricinus communis* |
| B9RPF8 | 0 | 95.23 | 11.20 | Abscisic acid-insensitive 5-like protein 6. ABF3= *Ricinus communis* |
| Q8RXD3 | 0 | 7.52 | 0 | E3 ubiquitin-protein ligase AIP2 (EC 6.3.2.-) (ABI3-interacting protein 2). AIP2 At5g20910 F22D1.80 |
| Q38913 | 25.02 | 115.28 | 403.93 | Extensin-1 (AtExt1) (AtExt4). EXT1 EXT4 At1g76930 F22K20.3 |
| Q43133 | 9.10 | 0 | 0 | Geranylgeranyl pyrophosphate synthase. chloroplastic/chromoplastic (GGPP synthase) (GGPS) = *Sinapis alba* |
| B9S473 | 12.75 | 0 | 0 | Abscisic acid receptor PYR/PYL = *Ricinus communis* |
| Q9M3V1 | 6.45 | 0 | 13.72 | Protein phpsphatase 2C (PP2C) (EC 3.1.3.16). pp2C1 = *Fagus sylvatica* |
| B9RNU7 | 5.54 | 0 | 0 | Protein phpsphatase 2C (PP2C) = *Ricinus communis* |
| Q38882 | 4.52 | 0 | 0 | Phospholipase D alpha 1 (AtPLDalpha1) (PLD alpha 1) (EC 3.1.4.4) (Choline phosphatase 1) (PLDalpha) (Phosphatidylcholine-hydrolyzing phospholipase D 1). PLDALPHA1 PLD1 At3g15730 MSJ11.13 |
| Q93VD6 | 80.35 | 0 | 0 | Abscisic acid response protein Asr1 = *Cucumis melo* |
| O04331 | 27.67 | 10.83 | 21.66 | Prohibitin (Prohibitin 3). Atphb3 At5g40770/K1B16.2 At5g40770 |
| Q9M3V1 | 6.45 | 0 | 13.72 | Protein phpsphatase 2C (PP2C) (EC 3.1.3.16). pp2C1 = *Fagus sylvatica* |
| B9RNW5 | 0 | 15.11 | 68.02 | SnRK2 putative = *Ricinus communis* |
| P18064 | 0 | 0 | 22.62 | Guanine nucleotide-binding protein alpha-1 subunit (GP-alpha-1). GPA1 At2g26300 T1D16.6 |
