# Supplementary material for: Transcriptomic Events Involved in Melon Mature-Fruit Abscission Comprise the Sequential Induction of Cell-Wall Degrading Genes Coupled to a Stimulation of Endo and Exocytosis
Source: PLoS One. 2013 Mar 6;8(3):e58363. doi: 10.1371/journal.pone.0058363 (PMC3590154; doi:10.1371/journal.pone.0058363)
Supplement: Table S14 — GA-, BR-, CK-, JA- and SA-related genes induced or repressed in fruit-AZ during melon MFA. Sequences were selected after establishing a P<0.01.The table shows the total read count in RPKMx1000 for each gene after normalization across the 3 samples: (a) AZ pre-cell separation (36 DPA), (b) AZ partial-cell separation (38 DPA), (c) almost complete-cell separation (40 DPA). (DOC) [file pone.0058363.s025.doc]

**Table S14** BR-, CK-, JA-, SA- and GA-related genes induced or repressed in fruit-AZ during melon MFA. Sequences were selected after establishing a P<0.01.The table shows the total read count in RPKMx1000 for each gene after normalization across the 3 samples: (a) AZ pre-cell separation (36 DPA), (b) AZ partial-cell separation (38 DPA), (c) almost complete-cell separation (40 DPA).

| **UniProt ID** | **36 DPA** | **38 DPA** | **40 DPA** | **Description** |
| --- | --- | --- | --- | --- |
| ***Brassinosteroid*** |  |  |  |  |
| B9RLU0 | 0 | 24.56 | 9.09 | Brassinosteroid LRR receptor kinase. putative: BRI1 = *Ricinus communis* |
| B9RUI5 | 0 | 13.84 | 4.42 | BRASSINOSTEROID INSENSITIVE 1-associated receptor kinase 1. putative. BAK1 = *Ricinus communis* |
| B9SC89 | 0 | 6.54 | 0 | BRASSINOSTEROID INSENSITIVE 1-associated receptor kinase 1. putative. BAK1 = *Ricinus communis* |
| Q9LR78 | 0 | 8.82 | 0 | Serine/threonine-protein phosphatase BSU1 (Bri1 suppressor protein 1). BSU1 At1g03445 At1g03450 F21B7.7 |
| Q9ZV88 | 0 | 19.48 | 0 | BES1/BZR1 homolog protein 4. BEH4 At1g78700 F9K20.26 |
| B9SGI1 | 7.36 | 12.61 | 0 | BRASSINAZOLE-RESISTANT 1 protein. putative. BZR1 = *Ricinus communis* |
| C0IRH2 | 0 | 46.60 | 0 | Xyloglucan endotransglucosylase/hydrolase 13 = *Actinidia deliciosa* |
| Q06BI5 | 26.71 | 0 | 0 | Xyloglucan endotransglucosylase/hydrolase 2. XTH2 = *Cucumis melo* |
| Q8GUQ5 | 0 | 0 | 1.93 | Brassinosteroid LRR receptor kinase (EC 2.7.11.1) (Altered brassinolide sensitivity 1) (Systemin receptor SR160) (tBRI1) . CURL3 *= Solanum lycopersicum* |
| ***Jasmonic acid*** |  |  |  |  |
| B9R7M7 | 0 | 7.07 | 0 | Cytochrome P450. putative (EC 4.2.1.92) = *Ricinus communis* |
| B9SK95 | 25.00 | 32.40 | 30.55 | 12-oxophytodienoate reductase opr. putative (EC 1.3.1.42) = *Ricinus communis* |
| B9T1J4 | 0 | 7.57 | 0 | Acyl-CoA dehydrogenase. putative (EC 1.3.3.6) = *Ricinus communis* |
| O65201 | 0 | 3.37 | 0 | Acyl-coenzyme A oxidase 2. peroxisomal (AOX 2) (EC 1.3.3.6) (Long-chain acyl-CoA oxidase) (AtCX2). ACX2 At5g65110 MQN23.4 |
| O65202 | 0 | 6.02 | 0 | Peroxisomal acyl-coenzyme A oxidase 1 (AOX 1) (EC 1.3.3.6) (Long-chain acyl-CoA oxidase) (AtCX1). ACX1 At4g16760 dl4405c FCAALL.119 |
| B9RKN5 | 11.97 | 58.47 | 12.98 | 3-hydroxyacyl-CoA dehyrogenase. putative (EC 5.1.2.3) = *Ricinus communis* |
| B9RWL7 | 26.81 | 172.46 | 24.63 | 3-ketoacyl-CoA thiolase B. putative (EC 2.3.1.16) = *Ricinus communis* |
| Q56WD9 | 0 | 56.27 | 14.43 | 3-ketoacyl-CoA thiolase 2. peroxisomal (EC 2.3.1.16) (Acetyl-CoA acyltransferase 2) (Beta-ketothiolase 2) (Peroxisomal 3-oxoacyl-CoA thiolase 2) (Peroxisome defective protein 1). PED1 KAT2 At2g33150 F25I18.11 |
| B0VXR3 | 0 | 6.32 | 0 | JAR1-like protein. JAR6 = *Nicotiana attenuata* |
| D8V3L7 | 0 | 31.69 | 11.73 | Plastid jasmonates ZIM-domain protein.JAZ putative = *Hevea brasiliensis* |
| B9S1E9 | 0 | 14.58 | 0 | Transcription factor AtMYC2. putative (EC 1.3.1.74) = *Ricinus communis* |
| Q9LTC4 | 0 | 22.22 | 0 | At3g23250 (MYB transcription factor 15) |
| Q38913 | 25.02 | 115.28 | 403.93 | Extensin-1 (AtExt1) (AtExt4). EXT1 EXT4 At1g76930 F22K20.3 |
| O22287 | 19.39 | 55.40 | 18.46 | At2g39770/T5I7.7 (CYT1 protein) (GDP-mannose pyrophosphorylase) (Putative GDP-mannose pyrophosphorylase). cyt1 GMP1 At2g39770 |
| B9S554 | 4.28 | 0 | 0 | 3-ketoacyl-CoA thiolase B. putative (EC 2.3.1.16) = *Ricinus communis* |
| B9ST81 | 74.34 | 61.95 | 14.86 | Jasmonate ZIM domain-containing protein. JAZ putative = *Ricinus communis* |
| O80575 | 14.68 | 4.40 | 0 | 6.7-dimethyl-8-ribityllumazine synthase. chloroplastic (DMRL synthase) (Lumazine synthase) (EC 2.5.1.9) |
| O04331 | 27.67 | 10.83 | 21.66 | Prohibitin (Prohibitin 3). Atphb3 At5g40770/K1B16.2 At5g40770 |
| Q9FNX8 | 0 | 0 | 1.79 | Lipoxygenase 4. chloroplastic (AtLOX4) (EC 1.13.11.12) (LOX3-like protein). LOX4 LOX3 At1g72520 F28P22.29 T10D10.1 |
| P43255 | 0 | 0 | 21.99 | COP9 signalosome complex subunit 8 (CSN complex subunit 8) (Constitutive photomorphogenesis protein 9) (Protein FUSCA 7). CSN8 COP9 FUS7 At4g14110 dl3095c |
| O49538 | 0 | 0 | 4.45 | At5g65790 (MYB transcription factor 68) (Myb - related protein) (Transcription factor-like protein). F6H11.100 At5g65790 |
| O04331 | 27.67 | 10.83 | 21.66 | Prohibitin (Prohibitin 3). Atphb3 At5g40770/K1B16.2 At5g40770 |
| B9R7M7 | 0 | 7.07 | 0 | Cytochrome P450. putative (EC 4.2.1.92) = *Ricinus communis* |
| B9T1J4 | 0 | 7.57 | 0 | Acyl-CoA dehydrogenase. putative (EC 1.3.3.6) = *Ricinus communis* |
| O65201 | 0 | 3.37 | 0 | Acyl-coenzyme A oxidase 2. peroxisomal (AOX 2) (EC 1.3.3.6) (Long-chain acyl-CoA oxidase) (AtCX2). ACX2 At5g65110 MQN23.4 |
| O65202 | 0 | 6.02 | 0 | Peroxisomal acyl-coenzyme A oxidase 1 (AOX 1) (EC 1.3.3.6) (Long-chain acyl-CoA oxidase) (AtCX1). ACX1 At4g16760 dl4405c FCAALL.119 |
| B9RKN5 | 11.97 | 58.47 | 12.89 | 3-hydroxyacyl-CoA dehyrogenase. putative (EC 5.1.2.3) = *Ricinus communis* |
| B9RWL7 | 26.81 | 172.46 | 24.63 | 3-ketoacyl-CoA thiolase B. putative (EC 2.3.1.16) = *Ricinus communis* |
| B9ST81 | 74.34 | 61.95 | 14.86 | Jasmonate ZIM domain-containing protein. JAZ putative = *Ricinus communis* |
| D8V3L7 | 0 | 31.69 | 11.73 | Plastid jasmonates ZIM-domain protein.JAZ putative = *Hevea brasiliensis* |
| B9S1E9 | 0 | 14.58 | 0 | Transcription factor AtMYC2. putative (EC 1.3.1.74) = *Ricinus communis* |
| Q9LTC4 | 0 | 22.22 | 0 | At3g23250 (MYB transcription factor 15) |
| O22287 | 19.39 | 55.40 | 18.46 | At2g39770/T5I7.7 (CYT1 protein) (GDP-mannose pyrophosphorylase) (Putative GDP-mannose pyrophosphorylase). cyt1 GMP1 At2g39770 |
| ***Salicylic acid*** |  |  |  |  |
| C5IJB4 | 10.14 | 0 | 0 | Phenylalanine ammonia-lyase. PAL = *Litchi chinensis* |
| O04331 | 27.67 | 10.83 | 21.66 | Prohibitin (Prohibitin 3). Atphb3 At5g40770/K1B16.2 At5g40770 |
| A5BPT8 | 0 | 114.83 | 0 | Phenylalanine ammonia-lyase. PAL = *Vitis vinifera* |
| A5YMH3 | 0 | 97.95 | 0 | Phenylalanine ammonia-lyase. PAL = *Astragalus penduliflorus* |
| B9S0K2 | 0 | 485.52 | 111.11 | Phenylalanine ammonia-lyase. PAL = *Ricinus communis* |
| O64963 | 0 | 85.54 | 0 | Phenylalanine ammonia-lyase. PAL1 = *Prunus avium* |
| B9S3I0 | 0 | 3.95 | 0 | Regulatory protein NPR1. putative = *Ricinus communis* |
| B9S7U9 | 37.26 | 18935.81 | 548.65 | STS14 protein. putative (PR-1) = *Ricinus communis* |
| Q38913 | 25.02 | 115.28 | 403.93 | Extensin-1 (AtExt1) (AtExt4). EXT1 EXT4 At1g76930 F22K20.3 |
| Q3E919 | 0 | 0 | 5.92 | At5g26920 |
| Q9M6E7 | 0 | 0 | 10.16 | UDP-glucose:salicylic acid glucosyltransferase. SA-GTase = Nicotiana tabacum |
| O49538 | 0 | 0 | 4.45 | At5g65790 (MYB transcription factor 68) (Myb - related protein). F6H11.100 At5g65790 |
| O04331 | 27.67 | 10.83 | 21.66 | Prohibitin (Prohibitin 3). Atphb3 At5g40770/K1B16.2 At5g40770 |
| Q38913 | 25.02 | 115.28 | 403.93 | Extensin-1 (AtExt1) (AtExt4). EXT1 EXT4 At1g76930 F22K20.3 |
| A5BPT8 | 0 | 114.83 | 0 | Phenylalanine ammonia-lyase. PAL = *Vitis vinifera* |
| A5YMH3 | 0 | 97.95 | 0 | Phenylalanine ammonia-lyase. PAL = *Astragalus penduliflorus* |
| B9S0K2 | 0 | 485.52 | 111.11 | Phenylalanine ammonia-lyase. PAL = *Ricinus communis* |
| O64963 | 0 | 85.54 | 0 | Phenylalanine ammonia-lyase. PAL1 = *Prunus avium* |
| P45732 | 0 | 133.79 | 30.30 | Phenylalanine ammonia-lyase. PAL17.1 = *Stylosanthes humilis* |
| B9S3I0 | 0 | 3.95 | 0 | Regulatory protein NPR1. putative = *Ricinus communis* |
| B9S7U9 | 37.26 | 18935.81 | 548.65 | STS14 protein. putative (PR-1) = *Ricinus communis* |
| ***Cytokine*** |  |  |  |  |
| B9RK75 | 0 | 13.86 | 0 | Gulonolactone oxidase. putative (EC 1.5.99.12) = *Ricinus communis* |
| Q0WR65 | 0 | 10.25 | 0 | Cytokinin oxidase-like protein. At3g63440 |
| D7TAZ7 | 0 | 1.92 | 0 | Histidine kinase 2/3/4 (cytokinin receptor). HK putative = *Vitis vinifera* |
| B9RMD2 | 0 | 2.98 | 0 | Histidine kinase 2/3/4 (cytokinin receptor). HK putative = *Ricinus communis* |
| B9RSW5 | 47.61 | 75.75 | 19.48 | Histidine-containing phosphotransfer protein. AHP putative = *Ricinus communis* |
| B9RMP7 | 0 | 3.42 | 0 | Two-component sensor histidine kinase bacteria. putative. two-component response regulator ARR-B family = *Ricinus communis* |
| D7TTG7 | 0 | 90.36 | 0 | Two-component sensor histidine kinase bacteria. putative. two-component response regulator ARR-B family = *Vitis vinifera* |
| Q8RUN2 | 12.51 | 0 | 0 | Cytokinin riboside 5'-monophosphate phosphoribohydrolase LOG1 (EC 3.2.2.n1) (Protein LONELY GUY 1). LOG1 At2g28305 T1B3.18 T3B23.2 |
| ***Gibberellin*** |  |  |  |  |
| B9RZI6 | 0 | 55.87 | 0 | Gibberellin 20 oxidase. putative (EC 1.14.11.11) = *Ricinus communis* |
| B9T711 | 0 | 2.93 | 0 | DELLA protein GAI. putative = *Ricinus communis* |
| B9SP75 | 11.49 | 19.15 | 6.02 | DELLA protein GAIP-B. putative = *Ricinus communis* |
| B9RIU5 | 3.25 | 24.39 | 7.58 | DELLA protein GAI. putative *= Ricinus communis* |
