# Supplementary material for: Transcriptomic Events Involved in Melon Mature-Fruit Abscission Comprise the Sequential Induction of Cell-Wall Degrading Genes Coupled to a Stimulation of Endo and Exocytosis
Source: PLoS One. 2013 Mar 6;8(3):e58363. doi: 10.1371/journal.pone.0058363 (PMC3590154; doi:10.1371/journal.pone.0058363)
Supplement: Table S15 — Transcription factor genes induced or repressed in fruit-AZ during melon MFA. Sequences were selected after establishing a P<0.01.The table shows the total read count in RPKMx1000 for each gene after normalization across the 3 samples: (a) AZ pre-cell separation (36 DPA), (b) AZ partial-cell separation (38 DPA), (c) almost complete-cell separation (40 DPA). (DOC) [file pone.0058363.s026.doc]

**Table S15** Transcription factor genes induced or repressed in fruit-AZ during melon MFA. Sequences were selected after establishing a P<0.01.The table shows the total read count in RPKMx1000 for each gene after normalization across the 3 samples: (a) AZ pre-cell separation (36 DPA), (b) AZ partial-cell separation (38 DPA), (c) almost complete-cell separation (40 DPA).

| **UniProt ID** | **36 DPA** | **38 DPA** | **40 DPA** | **Description** |
| --- | --- | --- | --- | --- |
| *Cluster A* |  |  |  |  |
| *Subcluster A1* |  |  |  |  |
| D7TNK3 | 28.65 | 0 | 0 | Nsd = *Vitis vinifera* |
| B9RMT1 | 4.56 | 0 | 0 | F-box and wd40 domain protein. putative = *Ricinus communis* |
| B9SZX0 | 49.10 | 0 | 0 | Ethylene-responsive transcription factor 1B. putative = *Ricinus communis* |
| D7T1G5 | 17.39 | 0 | 0 | Leucine-zipper transcription factor. BZIP domain class transcription = *Vitis vinifera* |
| D9ZJF0 | 22.22 | 0 | 0 | WRKY domain class transcription factor. (WRKY11) = *Malus domestica* |
| D7U8U5 | 24.39 | 0 | 0 | BHLH domain class transcription factor. Basic helix-loop-helix (bHLH) domain = *Vitis vinifera* |
| D9ZIP0 | 52.40 | 0 | 0 | BHLH domain class transcription factor. Basic helix-loop-helix (bHLH) domain. BHLH16 = *Malus domestica* |
| B3VTV7 | 12.46 | 0 | 0 | R2R3 MYB60 transcription factor. MYB60 = *Vitis vinifera* |
| B9RTF5 | 20.94 | 0 | 0 | Aux/IAA protein. IAA4 = *Ricinus communis* |
| D7TBU1 | 12.34 | 0 | 0 | Aux/IAA protein = *Vitis vinifera* |
| D9ZIT8 | 18.51 | 0 | 0 | Zinc finger(C2H2L28). C2H2L domain class transcription factor = *Malus domestica* |
| E0CRP8 | 7.47 | 0 | 0 | Homeobox protein = *Vitis vinifera* |
| Q5D1M2 | 2.74 | 0 | 0 | Class III HD-Zip protein 5 = *Populus trichocarpa* |
| D7TRM3 | 7.14 | 0 | 0 | Homeobox protein = *Vitis vinifera* |
| D7TQQ8 | 9.55 | 0 | 0 | E2F/DP family protein = *Vitis vinifera* |
| D7MC32 | 6.71 | 0 | 0 | Transcription factor. ARALYDRAFT_493171 = *Arabidopsis lyrata* |
| Q8LLR1 | 19.12 | 0 | 0 | MADS-box protein 3. MADS3 = *Vitis vinifera* |
| *Subcluster A2* |  |  |  |  |
| D9ZJB2 | 9.22 | 7.38 | 10 | SCL domain class transcription factor. SCL13 = *Malus domestica* |
| B9S948 | 55.87 | 40.46 | 0 | Ethylene-responsive transcription factor. putative = *Ricinus communis* |
| B9RET1 | 12.85 | 4.81 | 0 | Transcription regulator. putative = *Ricinus communis* |
| Q5S004 | 579.94 | 192.41 | 75.88 | Ethylene response factor 1. ERF1 = *Cucumis sativus* |
| Q6V5J8 | 79.28 | 45.30 | 22.65 | AP2 transcription factor/ethylene response element = *Brassica oleracea* |
| D5L0Z9 | 94.89 | 86.37 | 0 | AP2 domain class transcription factor. AP2D15 = *Malus domestica* |
| B9RHG8 | 13.88 | 6.17 | 0 | BHLH domain class transcription factor. Basic helix-loop-helix (bHLH) domain = *Ricinus communis* |
| B9SA09 | 53.90 | 7.09 | 0 | BHLH domain class transcription factor. Basic helix-loop-helix (bHLH) domain = *Ricinus communis* |
| D1MDP4 | 19.04 | 6.34 | 0 | MADS box protein. Flowering locus C. FLC = *Vitis vinifera* |
| Q4F8B3 | 22.57 | 19.92 | 0 | MADS box protein. MADS2 = *Prunus persica* |
| Q9AYR8 | 68.96 | 19.70 | 0 | MADS-box protein. ERAF17 = *Cucumis sativus* |
| Q9LHJ8 | 243.75 | 202.08 | 125.00 | Zinc finger A20 and AN1 domain-containing stress-associated protein 5 (AtSAP5). SAP5 At3g12630 T2E22.6 T2E22.105 |
| A9PH58 | 20.16 | 12.09 | 0 | Aux/IAA protein = *Populus trichocarpa* |
| *Subcluster A3* |  |  |  |  |
| B9T6G3 | 55.34 | 15.44 | 27.02 | AP2 domain transcription factor RAP2.3. putative = *Ricinus communis* |
| D9ZIT4 | 19.68 | 0 | 7.38 | Zinc finger (C2H2L24). C2H2L domain class transcription factor = *Malus domestica* |
| D7U0G0 | 13.69 | 0 | 5.70 | Aux/IAA protein = *Vitis vinifera* |
| D7UCZ6 | 792.07 | 178.21 | 458.74 | NAC-A/B (NAC-alpha/beta) domain (1); UBA domain (1) = *Vitis vinifera* |
| A5C5T1 | 12.01 | 0 | 6.00 | Homeobox protein = *Vitis vinifera* |
| D7TIC9 | 57.33 | 0 | 12.00 | Homeobox protein = *Vitis vinifera* |
| *Cluster B* |  |  |  |  |
| *Subcluster B1* |  |  |  |  |
| Q8LRL5 | 0 | 17.54 | 0 | NAC domain protein. Nam-like protein 10. NH10 = *Petunia hybrida* |
| B9SYE3 | 0 | 18.31 | 0 | CCAAT-binding transcription factor subunit A. putative = *Ricinus communis* |
| D7TKH6 | 0 | 35.25 | 0 | CCAAT = *Vitis vinifera* |
| Q00LP6 | 0 | 18.29 | 0 | GRAS2 = *Solanum lycopersicum* |
| B9SRA7 | 0 | 22.86 | 4.06 | ARID Transcription factor = *Ricinus communis* |
| D3Y3E9 | 0 | 53.64 | 0 | DRE transcription factor 1 = *Vitis pseudoreticulata* |
| B9S1I4 | 0 | 46.66 | 0 | Transcription factor IWS1. putative = *Ricinus communis* |
| D0E574 | 11.82 | 89.83 | 49.64 | NAP-like transcription factor = *Vitis vinifera* |
| B9RD55 | 0 | 1.43 | 0 | Ccr4-not transcription complex. putative = *Ricinus communis* |
| Q9ZV88 | 0 | 19.48 | 0 | BES1/BZR1 homolog protein 4. BEH4 At1g78700 F9K20.26 |
| B9SPF4 | 8.91 | 178.25 | 0 | Nut2. putative = *Ricinus communis* |
| D8VD38 | 0 | 1680.00 | 0 | Ethylene response factor 11. ERF11 = *Actinidia deliciosa* |
| Q2I2S8 | 230.88 | 821.10 | 62.69 | Ethylene-responsive element-binding protein. ERF-6 = *Medicago truncatula* |
| Q70AB2 | 0 | 5.29 | 0 | Ethylene transcription factor = *Fagus sylvatica* |
| D5L113 | 17.46 | 66.95 | 0 | AP2 domain class transcription factor. AP2D29 = *Malus domestica* |
| D7SVY0 | 0 | 43.12 | 0 | HSF family protein = *Vitis vinifera* |
| A5BUH0 | 45.19 | 85.68 | 58.38 | HSF family protein = *Vitis vinifera* |
| B9RP68 | 0 | 78.89 | 23.66 | BZIP domain class transcription. Transcription factor hy5. putative = *Ricinus communis* |
| B9RPF8 | 0 | 95.23 | 11.20 | BZIP domain class transcription = *Ricinus communis* |
| B9SU10 | 0 | 21.17 | 0 | BZIP domain class transcription. Transcription factor RF2a = *Ricinus communis* |
| D7SU43 | 0 | 27.84 | 0 | BZIP domain class transcription = *Vitis vinifera* |
| D7TIQ1 | 6.65 | 48.04 | 7.39 | BZIP domain class transcription = *Vitis vinifera* |
| D7TX81 | 0 | 24.16 | 0 | BZIP domain class transcription = *Vitis vinifera* |
| D7UCK3 | 0 | 46.51 | 10.07 | BZIP domain class transcription = *Vitis vinifera* |
| B2G284 | 0 | 9.95 | 0 | WRKY family transcription factor (WRKY7) = *Vitis thunbergii* |
| B9RNB2 | 11.56 | 19.97 | 0 | WRKY transcription factor = *Ricinus communis* |
| B9RXH5 | 0 | 15.51 | 2.87 | WRKY transcription factor = *Ricinus communis* |
| B9SVK5 | 0 | 28.75 | 0 | WRKY transcription factor = *Ricinus communis* |
| D9ZJF1 | 0 | 9.44 | 0 | WRKY transcription factor. WRKY12 = *Malus domestica* |
| D9ZJG1 | 0 | 25.10 | 0 | WRKY transcription factor. WRKY6 = *Malus domestica* |
| Q5IY47 | 0 | 11.19 | 0 | WRKY transcription factor. WRKY2 = *Vitis vinifera* |
| Q6R7N3 | 0 | 19.10 | 0 | WRKY transcription factor 30 = *Vitis aestivalis* |
| B9RBD1 | 20.63 | 214.28 | 20.63 | BHLH domain class transcription factor. Basic helix-loop-helix (bHLH) domain = *Ricinus communis* |
| B9RKM4 | 0 | 27.54 | 0 | BHLH domain class transcription factor. Basic helix-loop-helix (bHLH) domain = *Ricinus communis* |
| B9S1E9 | 0 | 14.58 | 0 | BHLH domain class transcription factor. Transcription factor AtMYC2 = *Ricinus communis* |
| D7T2X6 | 0 | 24.39 | 0 | BHLH domain class transcription factor. Basic helix-loop-helix (bHLH) domain = *Vitis vinifera* |
| E0CT50 | 0 | 263.00 | 14.06 | BHLH domain class transcription factor. Basic helix-loop-helix (bHLH) domain = *Vitis vinifera* |
| Q9M0B9 | 0 | 37.03 | 0 | BHLH transcription factor. At4g30410 |
| D9ZJ66 | 0 | 25.07 | 0 | MYB domain class transcription factor. MYB5 = *Malus domestica* |
| Q9LFL3 | 0 | 13.10 | 0 | AT5g16880/F2K13_30 (TOM (Target of myb1)-like protein). F2K13_30 At5g16880 |
| Q9LTC4 | 0 | 22.22 | 0 | MYB transcription factor 15. At3g23250 |
| D9ZJ82 | 130.24 | 188.74 | 81.67 | MYBR domain class transcription factor. MYBR3 = *Malus domestica* |
| D7U6A4 | 0 | 46.98 | 0 | MADS box protein = *Vitis vinifera* |
| D9ZIU5 | 0 | 20.83 | 0 | C2H2L domain class transcription factor. C2H2L9 = *Malus domestica* |
| B9SL96 | 0 | 36.01 | 5.54 | RING-type zinc finger protein. Makorin-1 = *Ricinus communis* |
| D7UCN8 | 0 | 10.07 | 9.06 | RING-type zinc finger protein = *Vitis vinifera* |
| B9SCR6 | 0 | 32.94 | 11.62 | Zinc finger protein = *Ricinus communis* |
| B9HUZ8 | 25.86 | 66.09 | 0 | Zinc finger = *Populus trichocarpa* |
| D7TTH5 | 0 | 8.15 | 0 | Zinc finger =*Vitis vinifera* |
| B9T724 | 0 | 21.35 | 0 | Zinc finger. GATA transcription factor. putative = *Ricinus communis* |
| B9RPQ5 | 0 | 236.64 | 25.44 | Zinc finger = *Ricinus communis* |
| A5BFH0 | 331.39 | 500.00 | 240.31 | Zinc finger =*Vitis vinifera* |
| D7TG75 | 12.34 | 46.29 | 28.80 | Zinc finger =*Vitis vinifera* |
| D9ZJA4 | 21.97 | 43.95 | 35.71 | NAC domain class transcription factor. NAC5 = *Malus domestica* |
| A5YWA9 | 65.57 | 73.22 | 26.22 | NAC domain protein. NAC = *Citrus sinensis* |
| B9RLW7 | 0 | 50.78 | 0 | NAC domain-containing protein 21/22 = *Ricinus communis* |
| B9T364 | 54.91 | 147.39 | 0 | NAC domain-containing protein = *Ricinus communis* |
| D7TXR6 | 200.00 | 220.83 | 137.50 | NAC-A/B (NAC-alpha/beta) domain = *Vitis Vinifera* |
| D9ZJ03 | 35.75 | 58.82 | 34.60 | HD domain class transcription factor. HD1 = *Malus domestica* |
| D9ZJ06 | 0 | 58.64 | 0 | HD domain class transcription factor. HD12 = *Malus domestica* |
| B9SDI4 | 0 | 43.69 | 4.41 | Bel1 homeotic protein = *Ricinus communis* |
| D7TM22 | 0 | 33.98 | 0 | Bel1 homeotic protein. putative = *Vitis vinifera* |
| D7SV02 | 0 | 45.66 | 0 | Bel1 homeotic protein. putative = *Vitis vinifera* |
| A5BGU8 | 0 | 205.61 | 0 | Homeobox protein = *Vitis vinifera* |
| A5C9F3 | 0 | 54.10 | 17.39 | Homeobox protein = *Vitis vinifera* |
| B9SVE1 | 0 | 173.28 | 18.05 | Homeobox protein = *Ricinus communis* |
| D7TNU6 | 26.14 | 146.59 | 0 | Homeobox protein = *Vitis vinifera* |
| D7TWR5 | 0 | 28.53 | 15.56 | Homeobox protein = *Vitis vinifera* |
| B9SEE4 | 0 | 55.34 | 24.45 | Transcription factor. putative = *Ricinus communis* |
| B9S5V4 | 0 | 16.23 | 0 | Transcription factor. putative = *Ricinus communis* |
| B9RCK4 | 3.93 | 10.83 | 0 | Transcription factor. putative = *Ricinus communis* |
| B9T2G9 | 0 | 16.62 | 0 | Transcription factor. putative = *Ricinus communis* |
| *Subcluster B2* |  |  |  |  |
| *Subcluster B3* |  |  |  |  |
| *Cluster C* |  |  |  |  |
| *Subcluster C1* |  |  |  |  |
| B9RZW3 | 0 | 0 | 7.28 | Tex protein-relatedtranscription accessory protein = *Ricinus communis* |
| D9ZJE1 | 0 | 0 | 5.29 | TLP domain class transcription factor. TLP4 = *Malus domestica* |
| Q3L0Q9 | 0 | 0 | 106.87 | Ethylene-responsive element binding protein ERF6 = *Gossypium hirsutum* |
| Q9FH54 | 0 | 0 | 8.06 | Ethylene-responsive transcription factor ERF114= ERF114 At5g61890 K22G18.1 |
| B9RNE6 | 0 | 0 | 18.51 | BZIP domain class transcription. Ocs element-binding factor = *Ricinus communis* |
| D9ZJE6 | 0 | 0 | 14.02 | WHY domain class transcription factor. WHY2 = *Malus domestica* |
| B9RIZ8 | 0 | 0 | 2.14 | BHLH domain class transcription factor. Basic helix-loop-helix (bHLH) domain = *Ricinus communis* |
| B9S8Y5 | 0 | 0 | 3.19 | R2r3-myb transcription factor = *Ricinus communis* |
| B9SYQ1 | 0 | 0 | 9.56 | R2r3-myb transcription factor = *Ricinus communis* |
| O49538 | 0 | 0 | 4.45 | MYB transcription factor 68. At5g65790 . F6H11.100 At5g65790 |
| A5AWY8 | 0 | 0 | 79.01 | Zinc finger protein. putative = *Vitis vinifera* |
| A5C6A5 | 0 | 0 | 70.55 | Zinc finger protein. putative = *Vitis vinifera* |
| B9T3B9 | 0 | 0 | 8.10 | Transcriptional activator. putative = *Ricinus communis* |
| D9ZJF5 | 6.84 | 6.84 | 26.39 | WRKY transcription factor. WRKY16 = *Malus domestica* |
| *Subcluster C2* |  |  |  |  |
| Q9SSY2 | 17.84 | 92.01 | 96.71 | Aux/IAA protein. CsIAA2 = *Cucumis sativus* |
| *Subcluster C3* |  |  |  |  |
