# Supplementary material for: Transcriptomic Events Involved in Melon Mature-Fruit Abscission Comprise the Sequential Induction of Cell-Wall Degrading Genes Coupled to a Stimulation of Endo and Exocytosis
Source: PLoS One. 2013 Mar 6;8(3):e58363. doi: 10.1371/journal.pone.0058363 (PMC3590154; doi:10.1371/journal.pone.0058363)
Supplement: Table S16 — PCR-primers used in this study. (DOC) [file pone.0058363.s027.doc]

**Table S16** PCR-primers used in this study.

| **Primer** | **Sequence** | **Gene (Uniprot ID)** |
| --- | --- | --- |
| EXT1-F | 5’-CCTTATTATGTATACAAATCACCT-3’ | EXT1-EXT4 (Q38913) |
| EXT1-R | 5’-AGGAGAGTGATAAATTGGTGGAGG-3’ |
| EXT3-F | 5’-GTGGCGACTCTAAGTTTGCCATCT-3’ | EXT3-EXT5 (Q9FS16) |
| EXT3-R | 5’-AGGAGGTGGTGGAGAGTGGTAAAC-3’ |
| PG2-F | 5’-GCTACCATTTATGTGCCTAAG-3’ | PG2 (ATIYVPK) |
| PG2-R | 5’-GGAAATGCAGTCATCACCAGT-3’ |
| EXP1-F | 5’-GCGGTTGAGCAACCGCCATT-3 | β-EXP1 (A1X8W4) |
| EXP1-R | 5’-AGCTGCAAAATAGTTCGAGTT-3’ |
| EXP7-F | 5’- GGATCAAGAACTGGTTGGCAAG-3’ | α-EXP7 |
| EXP7-R | 5’-GAATTGGGCACCACTATAAGT-3’ |
| EXP5-F | 5’-CCATGCCGTAAGCAAGGAGGA-3’’ | EXP5 |
| EXP5-R | 5’-GACACGGAAGTTCTTGCCGGTG-3’ |
| PE3-F | 5’-GTTCTTCAGGACTTGCAAAC-3’ | PE3 (Q43111) |
| PE3-R | 5’-ATACATCATAACATTCCACTT-3’ |
| PL-F | 5-TTGCAGAGCTTCAATTCTTCG-3 | PL |
| PL-R | 5’-ATTACGACCGAACCCAATGCC-3’ |
| RABA2B-F | 5’-ATGGCTCATAAGGTAGACCAC-3’ | RABA2B |
| RABA2B-R | 5’-CCTTCTGTTTGAGTCACCAGA-3’ |
| RABB1B-F | 5’-ATCATCATTGGTGATACTGGA-3’ | RABB1B (P92963) |
| RABB1B-R | 5’-AGCCCTTCGGTGTGCAAGATC-3’ |
| ARA5-F | 5’-CTGCTTATTGGAGATTCTGGT-3’ | RABD2A/ARA5 (B9MUT7) |
| ARA5-R | 5’-ACCAGACTTTTGGTTCACAGG-3’ |
| RABE1C-F | 5’-TATCTCATTAAGCTTTTGCTT-3’ | RABE1C/ARA3 (P28186) |
| RABE1C-R | 5’-ATCCATATCAGCCTTGTTTCC-3’ |
| ARA6-F | 5’-GTCCCAGACAGGGCTTCTGGG-3’ | RABF1/ARA6 (B9HUI6) |
| ARA6-R | 5’-CAACCGCTTCGCAATCTCCTC-3’ |
| RABH1B-F | 5’-GCAAGCCGGCAAACTTTCCTA.3’ | RABH1B (O80501) |
| RABH1B-R | 5’-TGTGGACTTCAAGTTAACATC-3’ |
| ACSa-F | 5’-GGATGGCGACTTGGGTGGTTTGT-3’ | ACS (B95BC0) |
| ACSa-R | 5’-TCAATATCATCATCACTGATATCTTC-3’ |
| ACSb-F | 5’- CCTTACTATCTTGATGAAGCATC-3’ | ACS ( Q9LN15) |
| ACSb-R | 5’- CCCTCTTGCTTGCAGAACTTCAC-3’ |
| ACOa-F | 5’-GGGGAGTGGGTGGACGTGCCTCC-3’ | ACO (C7U1K1) |
| ACOa-R | 5’-CGCCACCAGACTCGGCGCCGGAA-3’ |
| ACOb-F | 5’-CCCAACCTCATCACTCTTCTC-3’ | ACO (B9RYX6) |
| ACOb-R | 5’-ACAGCCCGATGAAGCACACTCTT-3’ |
| ACOc-F | 5’-GATCAAGTCCCCGGCCTGGAGTT-3’ | ACO (B9RKA0) |
| ACOc-R | 5’-ATAAAACGTAGCAATGGACAGCCT-3’ |
| ERF11-F | 5’-GGAGTCCGCCGGCGGCCGTGGG-3’ | ERF11 (D8VD38) |
| ERF11-R | 5’-ATTCAAAATGGCTTTACTTCCCCT-3’ |
| IAA2-F | 5’-ATGTCTCCACCATTACTTGATGTTG-3’ | IAA2 (Q9SSY2) |
| IAA2-R | 5’-TGTGTAGTTGGCTCTTTCACTTTCC-3’ |
| PR-1F | 5’-CAAGACTCACCTCGAGACTTCGTC-3’ | PR-1 (B9S7U9) |
| PR-1R | 5’-GGGTTCATAGTTGCAAATGATAAA-3’ |
| BRI-F | 5’-TTCAGCTTTGTCTACACAATCCTTA-3’ | BRI (B9RLU0) |
| BRI-R | 5’-CGCAAAAGAGGATTGCCGAGATACGA-3’ |
| SAP5-F | 5’-AATCCAGCGACGAACAATATG-3’ | SAP5 (Q9LHJ8) |
| SAP5-R | 5’-TACATCCAGAACAACGATTCA-3’ |
| ETR2-F | 5’-GTTGCTGACCAGGTGGCTGTTGCTCT-3’ | ETR2 (A8QYK9) |
| ETR2-R | 5’-ACCCCACCTTTGGTCATTCCGTCCCTG-3’ |
| ETR1-F | 5’-GTTGCTGACCAGGTGGCTGTTGCTCT-3’ | ETR1 (Q9SSY6) |
| ETR1-R | 5’-CATGAATTTTTGATGTATAGGGAAGCTT-3’ |
| ERS1-F | 5’-CAAATGAACAATTGGCCTGATGGCT-3’ | ERS1 (Q7GC82) |
| ERS1-R | 5’-TCGGCGTCCTCATTTCATGGTTCAT-3’ |
| ADC-F | 5’-TTGGCGCTTATAGCTAGGAAGCTTG-3’ | ADC (B3Y023) |
| ADC-R | 5’-CCAAGCATGTTTTATACTCACCCAT-3’ |
| NCED-F | 5’-CCACTCCCTAAAACCGCCGACCCA-3’ | NCED (B9S0Z6) |
| NCED-R | 5’-GTCTTCTGACATCGCCAAAAGCCG-3’ |
| SNRK2-F | 5’-AAGGGATACGACGGTGCGAAGGTTG-3’ | SNRK2 (B9RVE0) |
| SNRK2-R | 5’-ATTTCATCTCGTAATCATCGTAGA-3’ |
| ABF3-F | 5’-ATGGGAAAGCCATTAGGCAGCATG-3’ | ABF3 (B9RPF8) |
| ABF3-R | 5’-AGCAGCAGACTCCCTATTCTTGAT-3 |
| GH3-F | 5’-GGTCGCCCCTCTCCTGATACCT-3’ | GH3 (081829) |
| GH3-R | 5’-GACCTGGTTAAGAATTCAGAGAT-3’ |
| TIR1-F | 5’-CAGATGACAAATGCTGCCCTTGTA-3’ | TIR1 (B8Y9B4) |
| TIR1-R | 5’-ATCATTCTCTCGTTGATGATCTCCAC-3’ |
| KAT-F | 5-GGACTACCCATTCTTGGTGTCTTTA-3 | KAT (B9RWL7) |
| KAT-R | 5-GCCCATTCCCGTCCCTATGCACT-3’ |
| JAZ-F | 5’-TTGTTTCCTCAAGAGGCGGGATT-3’ | JAZ (D8V3L7) |
| JAZ-R | 5-CTATCTTTTCTCTTCTCCAAGAA-3 |
| MYC2-F | 5-AACGAGCTGAAATCGAAGCTCCA-3 | MYC2 (B9S1E9) |
| MYC2-R | 5’-CATCAAATCATTCACTACAGAAAC-3 |
| PAL-F | 5’-ACGGCAAAGAAATTGCATGAAATG-3’ | PAL (B9S0K2) |
| PAL-R | 5-TGGCAATTTCAGCACCTTTAAAGCC-3 |
| NPR1-F | 5’-GCATTAGATTCAGACGATGTTGAA-3’ | NPR1 (B9S3I0) |
| NPR1-R | 5’-ATAATCCTTTGGCCTTGTTAACCT-3’ |
| BAK1-F | 5’- CGGCTCAATACTTGCAAGCTTGGT-3’ | BAK1 (B9RUI5) |
| BAK1-R | 5’-ATCCCCTTTCAGAGAAGCAACCAT-3’ |
| BZRI-F | 5’-TCTCCAACCTTCAGCCTTGTAGCA-3’ | BZRI (Q9ZV88) |
| BZRI-R | 5’-CCCAAGTGTAAGTTCAAGATCATC-3’ |
| ARRB-F | 5’-GCATTGTCAGCCATTTCGAGCAAA-3’ | ARRB (D7TTG7) |
| ARRB-R | 5’-ATGAACAACATGTTGCCAAATATT-3’ |
| DELLA GAI-F | 5’-TTATATCGGATGCCAGACGAGAGC-3’ | DELLA GAI (B9RIU5) |
| DELLA GAI-R | 5’-TCCCATCCTGGCACGCCACTTCCC-3’ |
| GAL1-F | 5’-GGAGGAACTAACTTTGGCCGA-3’ | GAL (B9HL7) |
| GAL1-R | 5’-AGTCCTCAAAATGCAGGCCAAC-3’ |
| GAL2-F | 5’-GGCAACGAACCATTGGCTTTAGAC-3’ | GAL (D7TB77) |
| GAL2-R | 5’-CTTAACCAAAGAAATCCCTCCGGG-3’ |
| GAL3-F | 5’-CCTCGGTCTTGGCTAAGGCCA-3’ | GAL (Q5CCP8) |
| GAL3-R | 5’-AGTTCCCAATGGCGTTCCAAA-3’ |
| GAL4-F | 5’-TGTGAAGAAGCATTAGTCAGC-3’ | GAL (B9SWC7) |
| GAL4-R | 5’-TCTTCGAGATACCTACTGGTTC-3’ |
| MYBR3-F | 5’-GGACGGAGGAAGAACACAAGCTTT-3 | MYBR3 |
| MYBR3-R | 5’-GTGGTGATATCGAAGAGGCTGGA-3’ |
